# Efficacy of early cardiac rehabilitation after acute myocardial infarction: Randomized clinical trial protocol

**DOI:** 10.1371/journal.pone.0296345

**Published:** 2024-01-10

**Authors:** Caroline Schon, Amanda Felismino, Joceline de Sá, Renata Corte, Tatiana Ribeiro, Selma Bruno

**Affiliations:** 1 University Hospital Onofre Lopes, Federal University of Rio Grande do Norte, Natal, RN, Brazil; 2 Department of Physical Therapy, Federal University of Rio Grande do Norte, Natal, RN, Brazil; Kurume University School of Medicine, JAPAN

## Abstract

The acute myocardial infarction (AMI) present high mortality rate that may be reduced with cardiac rehabilitation. Despite its good establishment in outpatient care, few studies analyzed cardiac rehabilitation during hospitalization. Thus, this study aims to clarify the safety and efficacy of early cardiac rehabilitation after AMI. This will be a clinical, controlled, randomized trial with blind outcome evaluation and a superiority hypothesis. Twenty-four patients with AMI will be divided into two groups (1:1 allocation ratio). The intervention group will receive an individualized exercise-based cardiac rehabilitation protocol during hospitalization and a semi-supervised protocol after hospital discharge; the control group will receive conventional care. The primary outcomes will be the cardiac remodeling assessed by cardiac magnetic resonance imaging, functional capacity assessed by maximal oxygen consumption, and cardiac autonomic balance examined via heart rate variability. Secondary outcomes will include safety and the total exercise dose provided during the protocol. Statistical analysis will consider the intent-to-treat analysis.

**Trial registration. Trial registration number**: Brazilian Registry of Clinical Trials (ReBEC) (RBR- 9nyx8hb).

## Introduction

Acute myocardial infarction (AMI) represents a worrying clinical condition due to its high prevalence and mortality worldwide [1,2]. Evidence showed that myocardial reperfusion using percutaneous coronary intervention (PCI) and antiplatelet therapy [3]. However, AMI still causes many deaths, presenting about 13.6% of in-hospital mortality and 10% of mortality after one year [2,4]. Moreover, about 20% of survivors are susceptible to a second cardiac event within a year, or to clinical conditions of greater severity and lethality due to the progression of atherosclerosis [5]. Thus, interventions to enhance AMI control are currently under study, seeking an official treatment.

The exercise-based cardiac rehabilitation (CR) presents high evidence level (1A), and it is included in several guidelines because its cardioprotective effect controls cardiovascular diseases progression [4,6–8]. In this sense, exercise-based CR is important in secondary prevention because it reduces cardiovascular mortality and rehospitalization, improving quality of life and functional capacity [6,7].

However, the CR was underused in the last decade due to low adherence after hospital discharge [2]. The early CR in the acute phase post-AMI was associated with reduced ventricular remodeling and improved left ventricular ejection fraction [9,10], improved quality of life [11], increased distance covered (six- minute walk test), reduced depression symptoms [12] and heart rate (HR) recovery, and increased exercise duration for ischemia onset [13]. Moreover, previous INTERHEART reports showed that patients with AMI submitted to exercise-based CR indirectly controlled modifiable factors related to a second cardiac event [14].

Despite the evidence, few studies analyzed in-hospital CR in patients with AMI. Babu et al. [13] review showed that in-hospital CR includes varied procedures and exercise intensities that may improve the functional capacity of patients with heart failure. However, the adequate time to start CR in patients recently submitted to PCI needs to be clarified, especially regarding the acute physiological effects of exercise on hemodynamic and electrical safety, and their relationship with the efficient dose of in- hospital exercise-based CR.

Therefore, this study aims to clarify the safety and efficacy of early in-hospital exercise-based CR in patients with AMI submitted to PCI, considering cardiac morphology, autonomic variables, and functional capacity. This study may help clinical decision-making on adequate onset of in-hospital exercise-based CR with safe exercise doses.

## Methods: Participants, interventions, and outcomes

### Study setting

This is a clinical trial protocol, controlled, randomized, single-blind with two groups (1:1 allocation ratio) and a superiority hypothesis, following Standard Protocol Items: Recommendations for Interventional Trials (SPIRIT) guidelines [15]. This study will be conducted in partnership between the Department of Physical Therapy of the Federal University of Rio Grande do Norte (UFRN) and the University Hospital Onofre Lopes (HUOL) located in Brazil.

### Eligibility criteria

We will include patients of both sexes aged between 18 and 70 years admitted to the intensive care unit (ICU), diagnosed with AMI (electrocardiogram and lab tests), with or without elevation of ST-segment and non-complicated (Killip-Kimball classification of I or II) [16,17], who present a successful PCI (TIMI Coronary Grade Flow 2 or 3) [17], and conscious of providing free and informed consent. The exclusion criteria will include musculoskeletal alterations limiting the proposed exercises, or signs and symptoms of ischemia or cardiac decompensation (i.e., ejection fraction < 50% after AMI, or atrial or ventricular arrhythmias).

### Sampling/Recruitment

**Fig 1:**
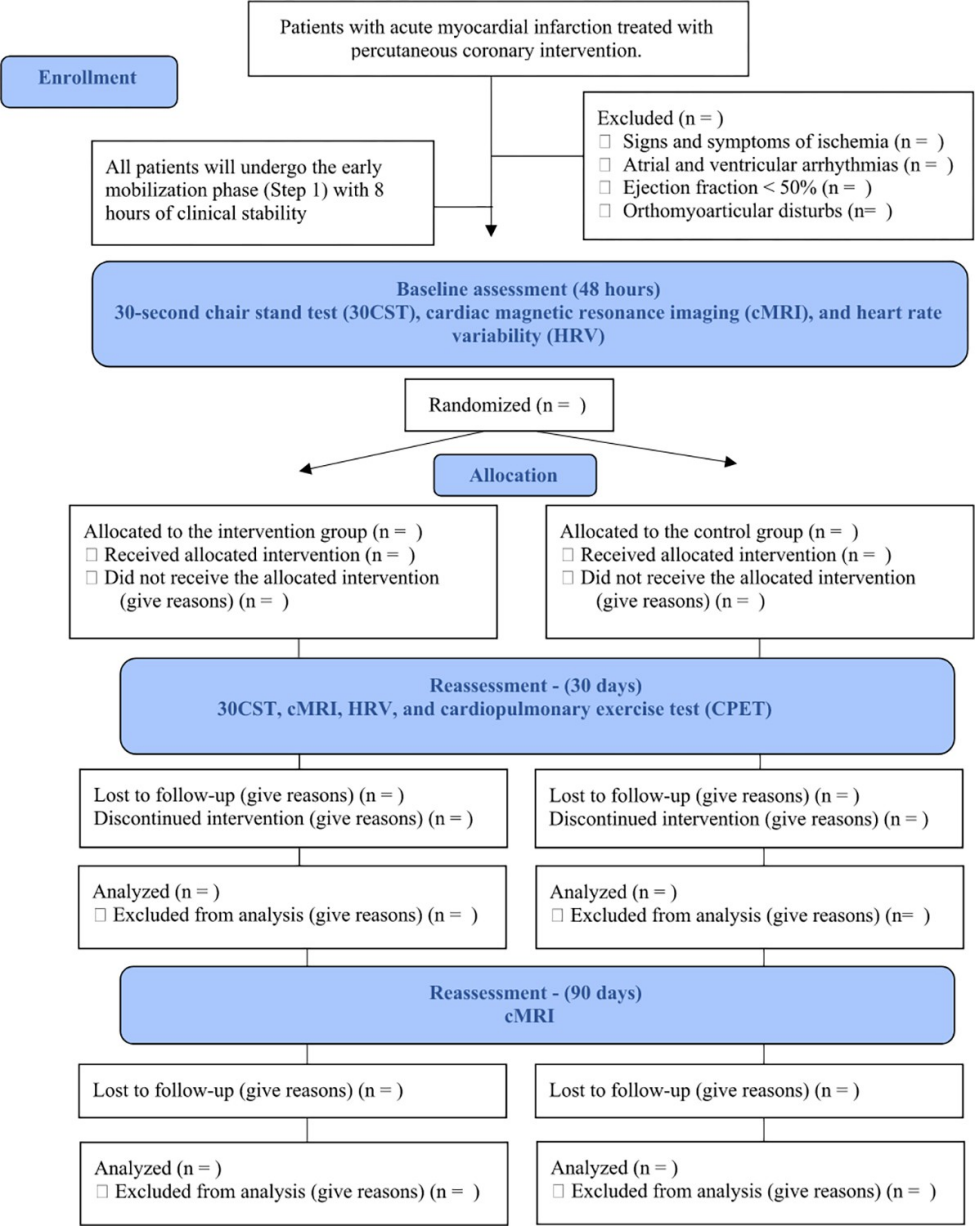
Flow diagram of the study protocol.

The population will comprise patients admitted to the ICU after the PCI procedure due to AMI, favoring the adhesion and involvement of the participants in the research. Recruitment and data collection began on November 23, 2022 and is expected to end on December 1, 2023. They will be followed up according to the flowchart based on CONSORT (Consolidated Standards of Reporting Trials) [18,19] (Fig 1). The sample size was calculated (OpenEpi software) using the mean difference of maximum oxygen consumption (VO_2max_ = 4.1 mL.kg^-1^.min^-1^), according to previous evidence that performed in-hospital exercise- based CR for VO2 analysis and a standard deviation of experimental and control groups (SD = 2.8 and 3.2, respectively) [20]. From these data, 14 patients (7 per group) were estimated according to a significance level of 5% and statistical power of 80%. The final sample size resulted in 24 patients (12 per group) after adding a 30% rate for possible dropouts. The sample calculation was also tested for the other primary variables of the study and verified that the total of 24 participants had equal power to verify differences in the other variables.

### Randomization/ Allocation/ Blinding

**Fig 2:**
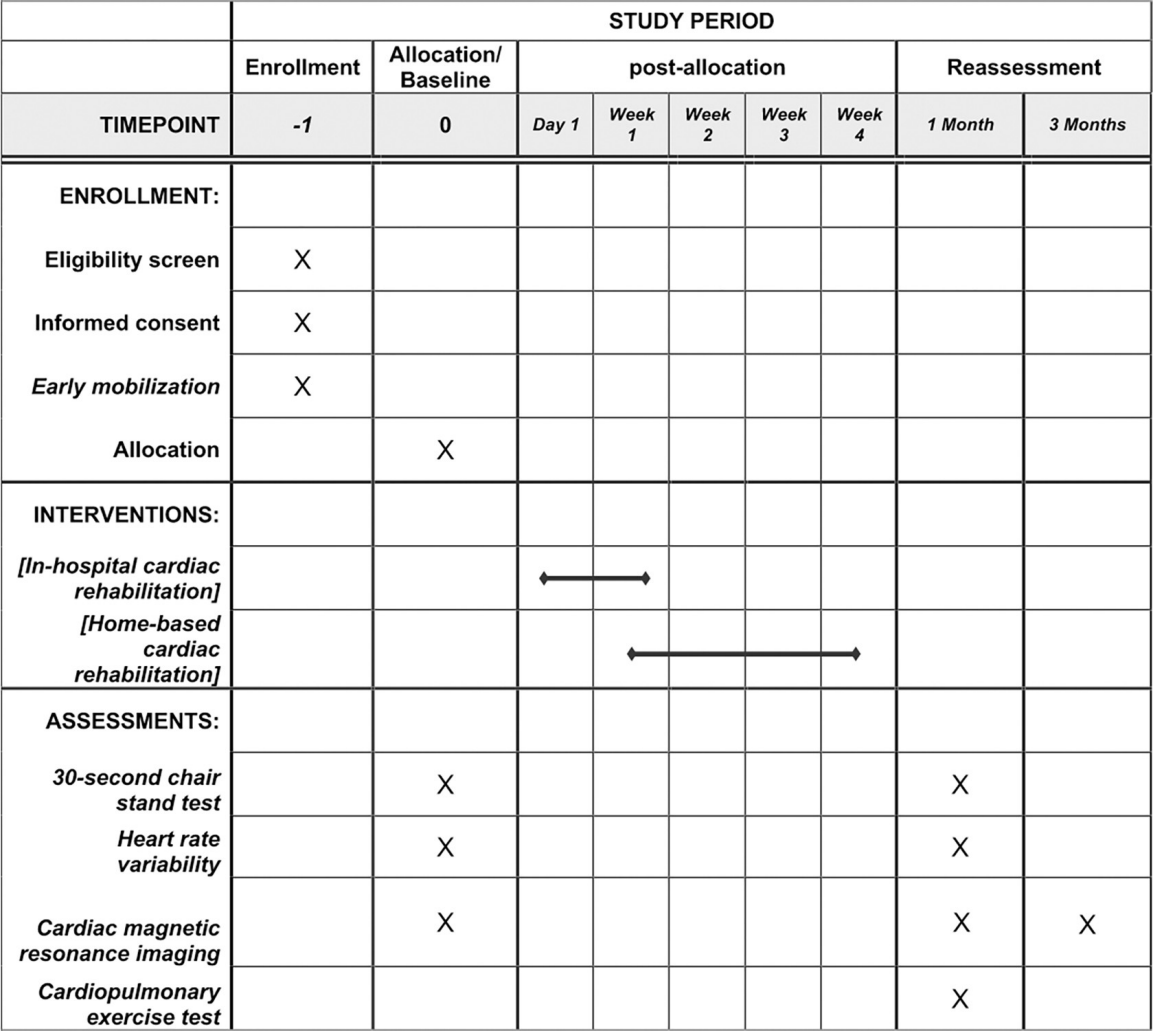
SPIRIT Schedule of enrolment, intervention, and assessments of the outcomes.

One researcher not involved in collecting or evaluating the results will perform the randomization (www.randomizer.org). Numbers will be randomly generated for two groups and concealed in sequentially numbered opaque envelopes. The outcome evaluators will be blind to the allocation, and data analysis will be performed with a coded spreadsheet. However, the researchers responsible for conducting the intervention will not be blinded. Fig 2 shows study timeline based on the SPIRIT guideline [15]. Allocation secrecy should be broken only in case of discontinuity of more than two consecutive sessions due to adverse events (AE).

### Interventions

The exercise-based CR protocol will follow all prescription parameters according to Frequency, Intensity, Time, Type, Volume, and Progression (FITT-VP), the American College of Sports Medicine [21], and the Consensus on Exercise Reporting Template (CERT) recommendation [19] to safety criteria. Patients will be informed about the procedures, possible risks and benefits of all study stages, and evaluated before and after the protocol. The physical therapists will receive equipment and protocol training to conduct all assessments and interventions standardly. Sessions will be recorded to verify adherence, and incentive commands will be given based on the clinical stability of each patient.

Initially, clinical and anthropometric data will be collected. Patients will be submitted to the early mobilization (Step 1: STEPS 1, 2, and 3) after eight hours of clinical stability (i.e., absence of signs and symptoms of ischemic), even under intravenous drugs and compressive dressing support. They will be evaluated and randomized after 48 hours of clinical stability [21]. Both groups will receive optimized medical treatment and permission to perform self-managed walking, besides training for HR self-monitoring and perceived exertion (Borg scale) [22]. After ICU discharge, the control group will be accompanied daily by the physical therapist, who will assess the clinical status and encourage the self-managed walking.

Patients from the intervention group will receive the exercise-based in-hospital CR in ICU (Step 1) and in the clinical ward (Step 2) (Tables 1 and 2). A semi- supervised protocol will be performed after hospital discharge (Step 3). The protocol will be individually conducted by a physical therapist twice a day during hospitalization, which varies from four to five days [23]; therefore, in-hospital sessions will range from eight to ten sessions. The intensity of aerobic training will be light, considering the metabolic equivalent of the task (METs) between 2 and 3 METs [24] based on perceived exertion (Borg scale between 9 and 12) [22].

**Table 1:** Exercise-based cardiac rehabilitation protocol–Step 1 (In-hospital phase in the intensive care unit).

STEPS	Clinical assessment	Type of exercise	Time	Intensity	Dose
**1**	Absence of signs and symptoms of cardiac ischemia > 8 hours (ECG, ischemic markers, or precordial pain) or cardiac decompensation.	Aerobic: lower limb cycle ergometer in bed. Upper and lower muscle endurance. (Shoulder flexion and abduction, quadriceps block, and triple flexion).	3 to 5 min 1 x 10 repetitions	Borg 9 to 10 (2 METs)	10 MET- min per session
**2**	Absence or low dose of inotropes, vasodilators, and intravenous antiplatelet administration.	Aerobic: lower limb cycle ergometer on the armchair. Upper and lower muscle endurance. (Shoulder flexion and abduction, quadriceps block, and triple flexion).	6 to 10 min 2 x 10 repetitions	Borg 9 to 10 (2 METs)	20 MET- min per session
**3**	Clinical stability according to STEPS 1 and 2.	Aerobic: lower limb cycle ergometer in armchair + walking (10 meters). Muscular endurance: 30-second chair-stand test.	9 to 15 min	Borg 9 to 10 (2 METs)	30 MET- min per session
**4**	Clinical stability according to STEPS 1 and 2.	Aerobic: lower limb cycle ergometer on armchair + walking (75 to 100 meters). Muscular endurance: sit to stand exercise.	9 to 15 min 2 x 60% test repetitions	Borg 9 to 10 (2 METs)	30 MET- min per session

min = minutes; The frequency of the STEPS is twice a day following the established order. The progression of the exercises focuses on the exercise time, following the order of the STEPS. The aim of the protocol is to achieve an accumulated 90 MET-min until ICU discharge.

**Table 2:** Exercise-based cardiac rehabilitation protocol—Step 2 (In-hospital phase in the ward).

STEPS	Clinical assessment	Type of exercise	Time	Intensity	Dose
5	Clinical stability according to STEPS 1 and 2.	Aerobic: walking + stair training. Muscular endurance: sit to stand exercise.	9 to 15 min 2 x 60% test repetitions	Borg 11 to 12 (3 METs)	45 MET- min per session
6	Clinical stability according to STEPS 1 and 2.	Aerobic: walking + stair training. Muscular endurance: Sit to stand exercise.	9 to 15 min 2 x 60% test repetitions	Borg 11 to 12 (3 METs)	45 MET- min per session
7	Clinical stability according to STEPS 1 and 2.	Aerobic: walking + stair training. Muscular endurance: sit to stand exercise.	12 to 20 min 3 x 60% test repetitions	Borg 11 to 12 (3 METs)	60 MET- min per session
8	Clinical stability according to STEPS 1 and 2.	Aerobic: walking + stair training. Muscular Endurance: sit to stand exercise.	12 to 20 min 3 x 60% test repetitions	Borg 11 to 12 (3 METs)	60 MET- min per session

min = minutes; The frequency of the STEPS is twice a day following the established order. The progression of the exercises focuses on time and intensity variables, following the order of the STEPS. The aim of the protocol is to guarantee 210 MET-min until discharge from the ward and 300 MET-min per week until hospital discharge, by the sum of the execution dose spent in ICU and ward.

The type of exercise will comprise resistance training associated with aerobic training (cycle ergometer in step 1 and ergometric bicycle in step 2). The resistance training will comprise two or three series corresponding to 60% of the maximum repetitions achieved in the 30-second chair-stand test (30CST) test without additional weight. The time of aerobic training will range from 3 to 20 minutes. The volume or dose of aerobic training will be assessed by the following formula: time x intensity (METs) x frequency of accumulated sessions and the total number of sessions x time, resulting in the training dose per session and week [25].

The step progression will be performed through out sessions according to a standardized sequence, considering the aerobic training time and series from resistance training [25]. However, the protocol prescription will respect the tolerance of the patient under the time recommended for each STEP and the intensity limit of the exercise.

Before hospital discharge, patients from the intervention group will receive a booklet with exercise guidance to perform continuous and semi-supervised training (i.e., home-based CR) (step 3) (Table 3). Patients will be instructed to perform aerobic training (walking) five times a week, maintaining the perceived exertion (Borg scale) between 9 and 12 to guarantee light to moderate intensity. The type of exercise will only be aerobic to facilitate adherence. The exercise time reached at the end of the in-hospital CR will be considered for initial prescription, and the progression will be based on the exercise time, increasing by five minutes weekly.

**Table 3:** Exercise-based cardiac rehabilitation protocol—Step 3 (Semi-supervised home phase).

STEPS	Clinical assessment	Type of exercise	Frequency	Time	Intensity	Dose
9	Clinical stability according to STEPS 1 and 2.	Aerobic: walking	5 per week	20 to 25 min	Borg 11 to 12 (3 METs)	300 MET-min per week
10	Clinical stability according to STEPS 1 and 2.	Aerobic: walking	5 per week	25 to 30 min	Borg 11 to 12 (3 METs)	375 MET-min per week
11	Clinical stability according to STEPS 1 and 2.	Aerobic: walking	5 per week	30 to 35 min	Borg 11 to 12 (3 METs)	450 MET-min per week
12	Clinical stability according to STEPS 1 and 2.	Aerobic: walking	5 per week	35 to 40 min	Borg 11 to 12 (3 METs)	525 MET-min per week

min = minutes; In stage 3, the frequency of the STEPS is weekly, and the progression of the exercises focuses on the time, increasing by 5 min per week. The weekly exercise dose will be calculated by multiplying the minimum time x frequency x intensity (METs).

A physical therapist will call the patients weekly to assess possible AE and guide them to maintain or discontinue the exercises. Patients will receive a textbook to record time spent exercising daily and weekly, which will be returned to the researcher upon reassessment (30 days after hospital discharge). This data will be used to estimate the volume of training performed.

A physical therapist from the hospital institution will conduct the in-hospital and semi-supervised exercise-based CR follow-up, seeking its maintenance. The session may be suspended if the patient presents AE (e.g., perceived exertion [Borg scale] > 13, reduced systolic blood pressure > 10mmHg, significant ventricular or atrial arrhythmias, signs or symptoms of exercise intolerance [i.e., dyspnea or angina], or electrocardiographic changes suggestive to ischemia). In addition, the protocol will be discontinued if the patient report AE in more than two sessions to ensure safety. In this case, a medical reassessment will be solicited, and the AE will be recorded in each medical report and follow-up form.

### Outcomes

The primary outcomes assessed will be cardiac remodeling (magnetic resonance imaging; cMRI), functional capacity using the VO2max in the cardiopulmonary exercise test (CPET), and cardiac autonomic balance using the heart rate variability (HRV); they will be expressed as mean and standard deviations. cMRI and HRV will be assessed during hospitalization about 48 hours after the PCI and 30 days after hospital discharge. CPET will be performed only on the reassessment day (30 days). The cMRI will be assessed during will also be reassessed after 90 days.

Secondary outcomes will include safety (AE ratio and the number of sessions performed during the study); the dose of aerobic exercise provided in each stage of the exercise-based CR, calculated by multiplying the frequency, time, and intensity of the aerobic exercise; and submaximal functional capacity (30CST), which will be performed during the initial assessment (48 hours of clinical stability) and at reassessment (30 days after hospital discharge).

## Methods: Data collection, management, monitoring and analysis

### Measurements, instruments and monitoring

The research group have a partnership with cardiologists and radiologists specialized in cardiovascular imaging at University Hospital Onofre Lopes. Also, the physical therapists enrolled in performing HRV and 30CST have theoretical and practical training. Strategies to ensure the adherence of patients will include the distribution of guidelines, a textbook to record training (intervention group), and phone calls to remind the date of reassessment.

The monitoring of the collected data will be done by a researcher who will not be part of the interventions or predictions, there will be no data monitoring committee (DMC) or sponsor influence in this study and any abnormal behavior of the results will be informed to the researcher in charge and he will be responsible for the decision to Terminate the study if serious adverse events occur.

### Functional capacity

The 30CST will assess functional capacity using a 45 cm-high chair without armrests [26,27]. The patient will be instructed to sit and stand up from the chair as fast as possible for 30 seconds. Both groups (intervention and control) will perform the test upon ICU discharge (48 hours after the PCI procedure) and at reassessment (30 days after hospital discharge). The results from the first test will be used to prescribe the resistance training during the intervention.

Patients will also perform the CPET at reassessment (30 days after hospital discharge) using a treadmill (Centurion 300, Micromed, Brazil) under an incremental load protocol (i.e., ramp protocol) to induce exhaustion between 8 and 12 minutes, according to ErgoPC Elite test (Micromed, Brazil) [28]. Before the test, HR will be verified using a resting electrocardiogram in 12 leads and electrocardiographic tracing (Digital ECG, Micromed, Brazil); peripheral oxygen saturation (Nonin 2500 oximeter) will also be monitored [29]. All patients will be instructed on the procedures and the Borg scale use to monitor perceived exertion [22].

All procedures will be performed under medical supervision, and patients will be authorized to lean on the front support bars of the treadmill if needed. The metabolic gas analyzer Cortex Metamax 3B (Germany) and the Metalyzer 3B software will capture, analyze, and interpret the expired gases. The variables of interest will be VO2peak, carbon dioxide production (VCO2), respiratory quotient (VCO2/VO2), test time, and oxygen pulse (VO2/HR).

### Cardiac remodeling

The cMRI will be assessed upon ICU discharge (48 hours after the PCI procedure) and at reassessment with 30 days and 90 days after hospital discharge to verify the extent of the infarcted area (cicatricial extension) and the transmural index. The morphology and cardiac function assessment (e.g., final systolic and diastolic volumes and the ventricular ejection fraction) will follow the Steady-State Free Precession (SSFP) with cMRI and the Late gadolinium enhancement techniques [30].

### Heart rate variability

HRV will be assessed in both groups at ICU discharge (48 hours after the PCI procedure) and reassessment (30 days after hospital discharge) using a chest strap-type heart rate monitor (Polar model H10). Data will be analyzed by Kubios HRV software. Linear and non-linear methods will analyze HRV. The variables of interest include the domain of time, such as the standard deviation of NN intervals (SDNN) recorded in a time interval and expressed in milliseconds (ms), and the percentage of successive RR intervals that differ by more than 50 ms (pNN50), while the domain of frequency will comprise low (LF; 0.04 to 0.15 Hz) and high frequency (HF; 0.15 to 0.4 Hz) and their ratio (LF/HF). Last, non-linear methods will enable Poincare graphical analysis and SD1, SD2, and SD2/SD1 variables [31].

### Safety

The safety of protocol comprises the presence of AE related to exercise-based CR, including diastolic blood pressure > 110 mmHg, drop in systolic blood pressure > 10 mmHg, chest pain symptoms or dyspnea associated with Borg score > 13, or electrocardiographic changes (i.e., atrial or ventricular arrhythmias) during or after sessions. The session will be interrupted in case of AE to guarantee clinical stability. The main researcher will monitor the AE, and patients who present more than two AE during sessions will be discontinued from the intervention. Moreover, the study will be discontinued if patients present high rates of AE.

### Ethics and dissemination

This clinical study was approved by the Research Ethics Committee of the university hospital. All study procedures will be under the norms established by the research ethics committee. Patients will be briefed about the research before providing their informed consent form by the lead researcher. Only the main researcher will tabulate numerically coded data to ensure confidentiality and will have access to the final study dataset in addition will be responsible for communicating any important adjustments during the application of this protocol. The findings of the study will be disclosed by the presentation of data at regional cardiology scientific events and submitted for publication in international journals. At the end of the research, all participants, regardless of the group, followed guidelines based on their individual functional tests to maintain or improve their physical fitness.

### Statistical analysis

Variables will be analyzed using SPSS (version 22.0) and Graph Pad Prism (version 7.0). The Shapiro-Wilk will verify data normality. Parametric data will be expressed as mean and standard deviation, whereas non-parametric data will be expressed in percentiles. Repeated-measures mixed ANOVA will verify intra-group comparisons of functional variables (30CST, HRV, and cardiac remodeling) considering the time factor. Mixed ANOVA and post hoc Bonferroni test will verify inter-group analyses considering VO2max, 30CST, HRV, and cardiac remodeling. Statistical significance will be set at p < 0.05. The study will analyze by intention to treat, analyzing groups according to randomization. For missing datas, data imputation will be performed, imputing the initial evaluation data of that participant.

## Discussion

The exercise-based CR is a well-established therapy for patients with ischemic heart disease [6,7]. However, worldwide societies in cardiovascular science do not provide formal guidance for this intervention in patients with AMI [4,32–34]. In addition, most evidence on this theme is from studies conducted before myocardial revascularization using PCI; therefore, these studies do not reflect the current therapy for this population. Nevertheless, this study will be conducted only in patients submitted to successful PCI.

Thus, this study will contextualize early exercise-based CR in patients submitted to PCI, and the first to describe the dose of exercise performed during hospitalization, the effects on morphology and cardiac automatism, and the functional capacity after exercise- based CR. Monitoring the exercise dose during exercise-based CR was set as the primary target to improve the quality of this intervention [25].

Furthermore, this study will offer a semi-supervised home-based CR for patients with AMI treated with PCI, seeking to maximize access and adherence. A Cochrane review on this theme observed that the effectiveness of home-based and in-hospital CR was similar. In addition, home-based programs presented high adherence and increased completion. However, the standardization of exercise-based CR performed at home still needs improvement [35]. This study will clarify the safety and effectiveness of this type of exercise-based CR.

## Supporting information

S1 Appendix Model consent form to participants. (PDF)

S1 File Reporting checklist for protocol of a clinical trial. (PDF)

S1 Protocol Protocol in Portuguese approved by the research ethics committee. (PDF)

S2 Protocol Protocol in English approved by the research ethics committee. (PDF)
