# Correction: Efficacy of early cardiac rehabilitation after acute myocardial infarction: Randomized clinical trial protocol

**DOI:** 10.1371/journal.pone.0300855

**Published:** 2024-03-14

**Authors:** Caroline Schon, Amanda Felismino, Joceline de Sá, Renata Corte, Tatiana Ribeiro, Selma Bruno

There is an error in the Funding statement. The correct Funding statement is as follows: This work has been financially supported by the Coordination for Personal Improvement of Higher Education (CAPES).
