# Supplementary material for: Efficacy of early cardiac rehabilitation after acute myocardial infarction: Randomized clinical trial protocol
Source: PLoS One. 2024 Jan 10;19(1):e0296345. doi: 10.1371/journal.pone.0296345 (PMC10781044; doi:10.1371/journal.pone.0296345)
Supplement: S1 Appendix — (PDF) [file pone.0296345.s001.pdf]

APÊNDICE D – TERMO DE CONSENTIMENTO LIVRE E ESCLARECIDO

TERMO DE CONSENTIMENTO LIVRE E ESCLARECIDO

O Sr. (a) está sendo convidado (a) como voluntário (a) a participar da pesquisa **"EFICÁCIA DA REABILITAÇÃO CARDÍACA PRECOCE APÓS INFARTO AGUDO DO MIOCÁRDIO: ENSAIO CLÍNICO RANDOMIZADO"**. Este estudo irá analisar os resultados da reabilitação cardíaca, utilizando-se da revisão do seu prontuário e de dois momentos de avaliações com exames físicos e de imagem. Esta pesquisa é importante para se entender o efeito da reabilitação cardíaca precoce sobre sua capacidade física e a função do seu coração de forma que os profissionais possam estabelecer formas mais eficazes de abordagem durante este processo.

Para este estudo adotaremos os seguintes procedimentos: Inicialmente serão analisados seus dados através de seu prontuário; Na primeira fase, o Sr.(a) participante será convidado a realizar um protocolo de reabilitação intra-hospitalar com sessões diárias de cerca 3 a 20 minutos e avaliação por meio de teste físico através do movimento de sentar e levantar de uma cadeira padronizada por 30 segundos e fará um exame de imagem do tipo ressonância magnética do coração, exame de imagem que dura cerca de 20 minutos, durante sua estadia no hospital. Na segunda etapa, após alta do hospital, você receberá um cartilha com orientações de exercícios para realizar em seu domicílio com sessões diárias de cerca de 20 a 40 minutos e após 30 dias do seu procedimento, você será novamente convidado a uma consulta ambulatorial no hospital para repetição do teste de sentar e levantar e realizar um teste na esteira ergométrica com duração média de 8 a 12 minutos e por fim realizará conforme marcação prévia um novo exame de imagem do seu coração através da ressonância cardíaca com a mesma duração do primeiro exame, cerca de 20 minutos.

Esta pesquisa poderá trazer benefícios a sua saúde física, emocional e capacidade de realizar suas atividades da vida diária através de uma prescrição individualizada dos exercícios físicos mais indicados e seguros a seremrealizados durante sua rotina, podendo inclusive diminuir o tempo da sua internação hospitalar e lhe dando mais segurança para sua alta hospitalar e qualidade de vida. Além disso, você receberá os laudos médicos a cerca do teste de esteira e ressonância magnética do seu coração, que poderá contribuir para um tratamento clínico mais eficaz junto ao seu cardiologista. Por fim, a sua participação nesta pesquisa ajudará no entendimento da melhor estratégia de reabilitação com exercícios após o infarto, facilitando o tratamento de diversos outros pacientes com este mesmo caso e favorecendo a publicação destes resultados para profissionais no mundo inteiro.

Rubrica do Participante/Responsável legal:	Rubrica do Pesquisador:

Esta pesquisa pode apresentar como possíveis riscos: A perda ou divulgação de seus dados pessoais; Cansaço, mudança na pressão arterial, aumento da frequência de batimento do seu coração e sinais de tontura após o exercício ou teste físico. Os possíveis riscos serão evitados ao máximo, pois estaremos monitorando continuamente os seus sinais vitais e interromperemos o teste ou o treinamento para que não haja nenhum risco ao participante, além de fornecer suporte clínico nas unidades de internação do hospital. Além disso, os seus dados pessoais serão protegidos através de um bom manuseio dos dados com uso de computador exclusivo para a pesquisa e seguiremos as normas da Lei Geral de Proteção dos Dados (LGPD), lei nº 13.709.

Para participar deste estudo você não terá nenhum custo, nem receberá qualquer vantagem financeira. Você será esclarecido (a) sobre o estudo em qualquer aspecto que desejar e estará livre para participar ou recusar-se a participar, podendo deixar de participar ou retirar seu consentimento em qualquer fase ou momento da pesquisa, sem nenhum prejuízo para você. Em caso de algum problema que você possa ter relacionado com a pesquisa, ou mesmo alguma dúvida, você terá direito a assistência gratuita que será prestada pela pesquisadora M.a. Caroline Ferreira Schon, telefone (84) 99900-0359. Se você sofrer algum dano comprovadamente decorrente dessa pesquisa, você receberá a assistência necessária para seu reparo. Seus dados serão confidenciais e divulgados apenas em congressos ou publicações científicas, não havendo divulgação de nenhuma informação que possa lhe identificar. Esses dados serão guardados pelo pesquisador por um período de 5 anos.

Qualquer dúvida sobre a ética dessa pesquisa você deverá entrar em contato com o comitê de ética em pesquisa do Hospital Universitário Onofre Lopes, telefono: 3342-5003, endereço: Av. Nilo Peçanha, 620- Petrópolis-Espaço João Machado-1ºandar-Prédio Administrativo-CEP 59.012-300-Natal/RN, e-mail. Este documento foi impresso em duas vias. Uma ficará com você e a outra com o pesquisador responsável M.a. Caroline Ferreira Schon.

Consentimento Livre e Esclarecido

Após ter sido esclarecido sobre os objetivos, importância e o modo como os dados serão coletados nessa pesquisa, além de conhecer os riscos, desconfortos e benefícios que ela trará para mim e ter ficado ciente de todos os meus direitos, concordo em participar da pesquisa (**EFICÁCIA DA REABILITAÇÃO CARDÍACA PRECOCE APÓS INFARTO AGUDO DO MIOCÁRDIO: ENSAIO CLÍNICO RANDOMIZADO)** e autorizo a divulgação das informações por mim fornecidas em congressos e/ou publicações científicas desde que nenhum dado possa me identificar.

Rubrica do Participante/Responsável legal:	Rubrica do Pesquisador:

Natal,__________de__________________de 2023.

____

Impressão datiloscópica do participante

Assinatura do participante da pesquisa

Como pesquisador responsável pelo estudo **(EFICÁCIA DA REABILITAÇÃO CARDÍACA PRECOCE APÓS INFARTO AGUDO DO MIOCÁRDIO: ENSAIO CLÍNICO RANDOMIZADO)**, declaro que assumo a inteira responsabilidade de cumprir fielmente os procedimentos metodologicamente e direitos que foram esclarecidos e assegurados ao participante desse estudo, assim como manter sigilo e confidencialidade sobre a identidade do mesmo.

Declaro ainda estar ciente que na inobservância do compromisso ora assumido estarei infringindo as normas e diretrizes propostas pela Resolução 466/12 do Conselho Nacional de Saúde – CNS, que regulamenta as pesquisas envolvendo o ser humano.

Natal,__________de__________________de 2023.

____

M.a. Caroline Ferreira Schon

Pesquisadora Responsável

Rubrica do Participante/Responsável legal:	Rubrica do Pesquisador:
