# Supplementary material for: Efficacy of early cardiac rehabilitation after acute myocardial infarction: Randomized clinical trial protocol
Source: PLoS One. 2024 Jan 10;19(1):e0296345. doi: 10.1371/journal.pone.0296345 (PMC10781044; doi:10.1371/journal.pone.0296345)

FEDERAL UNIVERSITY OF RIO GRANDE DO NORTE

POST GRADUATE PHYSICAL THERAPY PROGRAM

# EFFECTIVENESS OF EARLY CARDIAC REHABILITATION AFTER ACUTE MYOCARDIAL INFARCTION: RANDOMIZED CLINICAL TRIAL

NATAL – RN

2022

FEDERAL UNIVERSITY OF RIO GRANDE DO NORTE

POST GRADUATE PHYSICAL THERAPY PROGRAM

CAROLINE FERREIRA SCHON

# EFFECTIVENESS OF EARLY CARDIAC REHABILITATION AFTER ACUTE MYOCARDIAL INFARCTION: RANDOMIZED CLINICAL TRIAL

The project intended for evaluation by the Research Ethics Committee (CEP) of the Onofre Lopes University Hospital (HUOL).

**Concentration Area**: Evaluation and Intervention in Physiotherapy.
**Research Area:** Assessment and intervention in the Cardiovascular and Respiratory Systems.
**Professor Tutor:** PhD Selma Sousa Bruno

NATAL
-RN
2022

# SUMMARY


1. **INTRODUCTION** ........................................ 7

2. **OBJECTIVES** ........................................ 15

2.1 General objectives ........................................ 15

2.2 Specific objetives ........................................ 15

3. **MATERIAL AND METOD** ........................................ 16

3.1 Study design ........................................ 16

3.2 Study settings ........................................ 18

3.3 Participants ........................................ 18

3.4 Sampling and Recruitment ........................................ 19

3.5 Randomization and blinding. ........................................ 19

3.6 Early cardiac rehabilitation program ........................................ 20

3.5 Data collection procedures ........................................ 21

3.51 Anamnesis and clinical-physical evaluation ........................................ 22

3.52 Physical fitness analysis ........................................ 22

1. Cardiopulmonary exercise test (CPET) ........................................ 22
2. 30 second chair sit-to-stand test (30CST) ........................................ 25

3.53 Cardiac morphology and function analysis ........................................ 26

1. Cardiac morphology ........................................ 26
2. Heart rate variability (HRV) ........................................ 27

3.54. Analysis of aerobic exercise dose in cardiac rehabilitation ........................................ 27

3.55. Cardiohemodinamic safy of early cardiac rehabilitation ........................................ 27

3.6 Statistic strategy ........................................ 28

3.7 Ethical aspects ........................................ 28

4. **RISKS AND BENEFITS** ........................................ 29

5 **RESULTS AND IMPACTS EXPECTED** ........................................ 30

6 **TIMELINE** ........................................ 31

7 **BUDGET ESTIMATED**.......................................................................................... 33

8 **REFERENCES**.................................................................................................. 35

**APPENDICES**

**APPENDIX A** – Clinical evaluation form...............................................................................40
**APPENDIX B** – Cardiac rehabilitation protocol based on in-hospital exercise......................43
**APPENDIX C** – Prescription and follow-up form for early CR Step 3 .................................46
**APPENDIX D** – Informed consent term.................................................................................. 47
**APPENDIX E** – Term of institutional authorization for the use of patients documents......... 50
**APPENDIX F** – Guidance booklet for physical exercises ........................................................ 52

**ANNEXES**

**ANNEX A** – Perception of effort scale (BORG) .....................................................................54

# ABSTRACT

Exercise-based cardiac rehabilitation (CR) is a non-drug procedure already clinically incorporated with grade AI recommendation for post-acute myocardial infarction (AMI) patients in the outpatient phase. However, such evidence derives from studies in later phases after the cardiac event. CR in the in-hospital phase and early to the event has grown in the last decade, but its efficacy and safety during the procedure need to be clarified with more robust methodological designs. Thus, this study aims to analyze the effectiveness of a CR protocol based on physical exercises on physical fitness, morphology, and cardiac function, in addition to the cardiohemodynamic safety of inpatients with recent AMI. A controlled and randomized clinical trial will be carried out at the University Hospital Onofre Lopes (HUOL) with a CR protocol based on early physical exercise (>8 hours of clinical stability) of patients with AMI with success in percutaneous coronary intervention (PCI). After randomization, patients will be grouped into GI and CG (intervention and control group). GI will be submitted to a standard low-intensity CR protocol and CG to usual care and mobilization guidelines. Subjects in both groups will undergo the same functional assessments, maximal and submaximal (cardiopulmonary and 30s sit-to-stand tests), cardiac nuclear magnetic resonance, and analysis of heart rate variability. Safety scores and cardiohemodynamic events during rehabilitation will be analyzed. It is expected to observe, at the end of the study, a positive effect of in-hospital CR on cardiac structure and function and its clinical repercussions on the increase of physical fitness, allowing a greater understanding of exercise prescription variables and their physiological responses to predict more therapeutic strategies. efficient and early interventions during the in-hospital rehabilitation process in patients with AMI.

**Keywords:** Early cardiac rehabilitation; physical performance; exercise dose; acute myocardial infarction.

## LISTA DE ABREVIATURAS

**AACVPR** American Association of Cardiovascular and Pulmonary Rehabilitation

**CF** Cardiorespiratory fitness

**ACSM** American College of Sports in Medicine

**AHA** American Heart Association

**CVD** Cardiovascular Disease

**COPD** Chronic Obstructive Pulmonary Disease

**EF** Ejection Fraction

**HUOL** Hospital Universitário Onofre Lopes

**ICP** Percutaneus Coronary Intervention

**MIA** Myocardial Infarctation Acude

**MET** Metabolic Equivalent of Task

**CR** Cardiac Rehabilitation

**RCR** Reverse Cardiac Remodeling

**NRM** Nuclear Resonance Magnetic

**MR** Myocardial Revascularization

**ACS** Acute Coronary Syndrome

**CPET** Cardiopulmonary Exercise Test

**CST** CST Chair Sit-Stand Test

**6mWT** 6 Minute Walk Test

**HRV** Heart Rate Variability

# INTRODUCTION

## 1.0 Acute Coronary Syndrome (ACS) and Myocardial Infarction Acute (MIA)

Acute coronary syndrome (ACS) occurs due to a sudden reduction or absence of blood flow in the coronary arteries, resulting, therefore, in a perfusion deficit of the cardiac tissue. The clinical presentation of the syndrome can be broad and vary from the typical symptom of acute chest discomfort to electrical or hemodynamic instabilities that can progress to cardiorespiratory arrest (1,2). The treatment should be immediate, and it is recommended in this clinical condition, to perform reperfusion therapy by primary percutaneous coronary intervention (PCI), In case of non-availability, fibrinolytic therapy should be indicated (1,3) cardiohemodynamic events during rehabilitation will be analyzed. It is expected to observe, at the end of the study, a positive effect of in-hospital CR on cardiac structure and function and its clinical repercussions on the increase of physical fitness, allowing a greater understanding of exercise prescription variables and their physiological responses to predict more therapeutic strategies efficient and early interventions during the in-hospital rehabilitation process in patients with AMI.

The death of cardiomyocytes secondary to the process of inefficient perfusion by the affected coronary arteries can be defined by the term acute myocardial infarction (AMI). Myocardial necrosis can be verified through specific exams, the most recommended being the assessment of high sensitivity cardiac troponin (hs-cTn) which defines AMI with its elevation above the 99th percentile of the reference values (3).

Although there are variations between European countries, cardiac ischemia is responsible for approximately 1.8 million deaths annually (4). The most recent 2021 American Heart Association (AHA) Update shows a prevalence of 49.2% (126.9 million) of cardiovascular disease (including ischemic heart disease) in adults (5). Current studies demonstrate a reduction in acute and long-term mortality after AMI and correlate this fact to the greater use of reperfusion therapy by PCI, despite this fact, mortality after a continuous cardiac ischemic event generates substantial deaths with in-hospital mortality of about 13.6% and 1-year mortality of about 10% (3.5).

## 2.0 Cardiac Rehabilitation (CR)

Mortality after long-term AMI can also be positively influenced by another therapy that should be included in the management of patients with ischemic heart disease: cardiac rehabilitation (CR). CR is recommended with a high level of evidence (A) and a strong class of recommendation (I) based on extensive systematic reviews on the topic and duly recommended by clinical guides (3,6–8). CR promotes a reduction in cardiovascular and all-cause mortality and improves quality of life and functional capacity in this profile of patients (6,7). In a recent review on the subject, Cochrane showed a review of 14,486 patients with coronary disease who participated in CR and showed a significant reduction in cardiovascular mortality and hospitalization when compared to the control group (non-RC participants) (9). Clinical outcomes followed in follow-up studies are primarily associated with attenuation and better control of cardiovascular disease (CVD) risk factors by lifestyle change, including a more physically active lifestyle for individuals (10,11).

Cardiac rehabilitation is defined by World Clinical Guides as a multifaceted intervention process that aims to promote the integral recovery (physical and functional improvement as an anchor of this recovery) of patients affected by acute cardiac events or chronic cardiovascular diseases. The formal CR program should be considered an essential phase for all eligible patients, with only a few cases being recommended only home exercise guidance (9,12–21).

CR programming is traditionally subdivided into stages that obey a temporal, spatial logic and the level of clinical support that the patient needs so that the first offer of CR happens in the hospital phase, which aims at early mobilization and rehabilitation with exercise exercises. low intensity, guidance to the patient about their disease and modifiable risk factors, in addition to strategies for adopting a healthy lifestyle with the aim of providing hospital discharge with the best possible physical and mental condition. After hospital discharge, the patient must proceed to continue the extra-hospital or outpatient phases that occur immediately after discharge and is a prolonged stage that continues the process of increasing physical capacity and comes from maintaining active habits as a lifestyle.

Despite the wide scientific-clinical recommendation, there is still a low number of referrals and participation of patients in formal CR after hospital discharge, which points to a non-pharmacological treatment that is still underused, considering the importance of the relationship between clinical need and control of cardiovascular disease x low procedure cost. Data from the American Heart Association (AHA) update warns that only 24.4% of patients are eligible to participate in CR, with an average of 47 days to start rehabilitation. Furthermore, of those who participated in CR, only 26.9% of patients completed the program. Of these, approximately 93% are patients who underwent early cardiac rehabilitation while still in the hospital. These data show that greater investment is needed in the structuring and viability of cardiac rehabilitation sites for better control of cardiovascular disease. In addition, it also shows the need for continuity between the hospital and outpatient phases in order to optimize a continuum between control of future cardiac events.

In view of the previous findings, an issue that may be quite relevant is the absence of early initiation of the rehabilitation process in the hospital phase, which may be a key point in changing the culture of undervaluing CR both on the part of the team of cardiovascular professionals as well as by the patients themselves. The latest scope review on the subject also found that most of the body of current scientific evidence is based on experimental studies from the pre-PCI revascularization era, which does not reflect the current clinical context, in addition, the clinical guidelines of cardiovascular societies do not provide specific recommendations for the hospital phase of CR (22). Finally, there is a gap in scientific knowledge about the safe time to start CR, its benefits, safety, and prescription parameters in the in-hospital context.

## 3.0 Physical fitness after Cardiac Rehabilitation

The state of sedentary lifestyle is already pointed out in the scientific literature as a risk factor crucial for primary and secondary cardiac events (23). In correlation with this finding, other studies demonstrate the level of cardiorespiratory fitness (CRF), a component of physical fitness, as the greatest predictor of mortality compared to any other cardiovascular risk factor. Therefore, ACR has been evaluated in several studies that seek to verify the benefits of CR, due to its

notorious modification during treatment. Previous work has shown an average gain of 11 to 36% in ACR after traditional outpatient CR, with greater gains in patients who had lower ACR levels at the beginning of the program (24). Thus, there is sufficient evidence to prove that improvements in ACR can explain most of the physical, psychological, and functional benefits, as well as the clinical outcomes of mortality and hospitalization after carrying out a formal CR program (10,25–27).

Among the physiological effects promoted by the physical component of CR that may justify the ACR gains and their repercussions for the general health status after the program are: Attenuation of the severity of ischemia induced by exertion with improvement of angina at rest by raising ischemic thresholds and improvement of endothelial dysfunction, increase in peak oxygen consumption and maximal aerobic power with improvement in skeletal muscle oxidative capacity, reduction in neurohumoral exacerbation, favorable action on the lipid profile, especially in cases of hypertriglyceridemia, decreased levels of HDL-cholesterol and changes in LDL-cholesterol subfractions, hypotensive effect of exercise with reduction of systolic and diastolic pressure levels (9).

In the in-hospital phase of CR, some clinical trials also demonstrated improvement in relation to physical fitness components through indirect or estimated measures of the ACR, such as a significant increase in the distance covered in the six-minute walk test (6MWT) (28, 29). Despite this, many hospital services do not have adequate physical structure to carry out the 6MWT, which requires a flat and continuous corridor of 30 meters and more than one evaluator to perform the test. Other submaximal exercise tests have been used for functional assessment and estimation of physical fitness components. A less expensive possibility in relation to the number of evaluators and physical space is the 30-second sit-to-stand test. The 30s sit-to-stand test has already been validated for the elderly population and patients with Chronic Obstructive Pulmonary Disease (COPD), including established clinically relevant differences for follow-up effects with exercise intervention (30,31). No clinical trials, to date, have evaluated the validity and safety of this functional test with patients hospitalized after AMI.

The assessment of the effects of CR is traditionally verified through direct measurement of the ACR through a cardiopulmonary stress test, obtaining the direct measurement of the peak oxygen consumption (VO2 peak), a method recommended by several international guidelines (32,33 ). Only one CR hospital-phase clinical trial (29) performed the cardiopulmonary test to

assess peak VO2, 30 days after CR in the profile of patients after coronary artery bypass grafting (CABG). There is, however, a great lack of direct analysis of ACR in post-AMI clinical patients undergoing PCI.

## 4.0 Heart morfology after cardiac rehabilitation

Despite the well-documented effects of cardiac rehabilitation in patients with AMI with and without heart failure (HF), there is still uncertainty about this effect on ventricular remodeling. Ventricular remodeling is a broad term used to describe genetic, molecular, neurohumoral, cellular, and interstitial alterations that can culminate in changes in the geometry, mass, and function of the ventricle. In AMI, depending on the magnitude, this event can determine the emergence of acute or chronic cardiac dysfunction such as heart failure. The term reverse cardiac remodeling (RCR) has been used to define the partial reversal of this condition as a response to drug treatment, use of implantable resynchronizers, or more uncertainly, aerobic physical exercise, which have brought about improvement in survival and quality of life (34).

Aerobic physical training has still conflicting results in the scientific literature and it is not clear how it can interfere with ventricular remodeling and contribute to reverse ventricular remodeling. Some studies reported attenuation of ventricular remodeling with physical training (35,36), while others worsened the progression of remodeling (37,38) and others, no relation with remodeling (39,40). Despite the divergent results, it is important to pay attention to the start time of cardiac rehabilitation programs that were not uniform in the studies and the clinical severity after AMI, as well as the level of dysfunction that can be verified by the left ventricular ejection fraction (LVEF).

Observing these possible factors interfering with these results, a systematic review with meta-analysis verified the influence of different times of onset of CR in post-AMI patients with reduced LVEF and observed that better changes in ventricular remodeling and cardiorespiratory capacity with early onset of CR, between 6h and 7 days (acute phase) without an increase in adverse events (41). Despite this, the studies included in the previous review addressed continued CR with a broad outpatient phase, thus, to our knowledge, there are no studies evaluating cardiac morphology immediately after the in-hospital phase as a way to assess the effectiveness of this phase of rehabilitation.

## 5.0 Cardiac autonomic function after cardiac rehabilitation

Heart rate variability (HRV) is an easy and non-invasive method to assess the integrity and function of the autonomic nervous system on heart rhythm, in addition to determining risks related to cardiac and non-cardiac diseases (42). HRV has been extensively described after AMI in assessing the prognosis after the event. One of the initial studies describing the relationship between HRV after AMI verified that the reduction of HRV in patients admitted to the critical care unit after AMI increased in-hospital mortality (43). Its prognostic value was later independently confirmed for predicting mortality (44). Despite the great scientific description of its prognostic value, few studies have been directed toward the search for therapeutic effects on HRV. A recent systematic review investigated the effect of physical exercise on HRV after AMI only in the outpatient phase of CR and showed that the results are still divergent, out of eleven clinical trials, five did not show any results on HRV, but six trials showed favorable results. in relation to better autonomic control after exercise-based CR (45). We still do not know the effect of CR on HRV in the in-hospital phase of CR

## 6.0 Cardiac rehabilitation dose and safety aspects

Even with the recognition of the effects and clinical studies of cardiac rehabilitation exercises being dose-dependent, there are important aspects to be considered in terms of dose versus safety in the procedure. The Kanazawa study, 2020, obtained a dose-response trend for in-hospital CR and clinical outcomes such as the risk of revascularization and hospital readmission for all causes, obtaining a significant reduction of these steps in the group of patients who underwent the a from 8 CR sessions during a hospital stay (46). Despite this, no clinical trial located the numerical dose prescribed to patients, matching the prescription parameters and providing a specific targeting of the dose of exercises performed during the in-hospital phase of CR and, therefore, the need to safely guarantee the benefits to be achieved. health. The lack of standardization of dose x cardiac safety in the procedure weakens the adoption of early and safe strategies for maintenance or even the adoption of CR procedures after the cardiac event in an early hospital phase.

Another recent study by Keteyian et al., 2018, retrospectively analyzing a large sample of patients who underwent CR over 5 years, showed important findings. Patients who increased their training workload by 1 MET at the end of CR had a 38% lower adjusted risk and a 42% lower chance of hospitalization and all-cause mortality. This fact points to the need for monitoring the exercise prescription variables during CR as a way to ensure the greatest possible ACR gain for each patient, considering its impact on survival (47). This message should also be incorporated during the initial in-hospital CR prescription.

Numerous evidence therefore demonstrate the potential effect of ACR on clinical outcomes. The increase in ACR and its protective effect is directly related to the dose of aerobic exercise prescribed during CR. The dose of aerobic exercise can be defined as the total energy expended in exercise during a week of training and mathematically described through the analysis of training prescription parameters, such as frequency, intensity, and time. The position on the progression of the exercise dose in CR by the AACVPR (American Association of Cardiovascular and Pulmonary Rehabilitation) together with the ACSM (American College of Sports Medicine) in 2018, demonstrates that the dose value of aerobic exercise between 500-1000 METxmin/week generate a significant reduction in premature mortality and cardiovascular diseases (48).

In view of the findings regarding the theme of the CR results on functional capacity and its impact on the morbidity and mortality of patients, there was a lack of studies that focused on dosing the aerobic exercise prescribed during in-hospital CR and determining the efficacy and safety of this dose. In addition, knowing that the higher the level of cardiorespiratory fitness at the end of CR, the better the survival rate, it is notably important to study the impact of exercise dosage in the search for optimization of CR results, since there are still patients who are little or no responders in relation to ACR gain after the program.

This study becomes necessary, therefore, considering the importance of determining the ideal dose of aerobic training during in-hospital CR in the search for optimizing the results in RCA and its impact on the clinical prognosis of patients compared to the conventional CR process. Serving as a model for calculating the dose of aerobic training that is easy to include in the clinical routine of in-hospital CR sectors. It is also important to verify the cardiac morphological and functional repercussions of early CR, as well as the clinical impact of this change on the physical and functional aspects of the patient. Finally, it will establish the level of cardiohemodynamic

safety of this therapy in an early phase.

Thus, the general objective of this study will be to describe the dose of aerobic exercise offered during early CR and its effect on physical fitness, morphology cardiac function, and safety aspects in patients after AMI.

# STUDY OBJECTIVES

## Primary objective

To evaluate the efficacy and safety of early CR after AMI and its effect on physical fitness, morphology, and cardiac function.

## Secondary objectives

- To assess the variation in functional capacity by measuring cardiorespiratory fitness after in-hospital CR, using maximum VO2 (maximum oxygen consumption) as a result measure;
- To analyze the dose of aerobic training provided to patients in in-hospital CR and its impact on the outcome of functional capacity;
- Relate the standard method of measuring VO2, the cardiopulmonary test, to a submaximal evaluation method, the 30s sit-to-stand test, evaluating its validity and reproducibility;
- Observe the influence of early CR on the morphological parameters of ventricular remodeling through nuclear magnetic resonance (NMR);
- Investigate heart rate variability after early cardiac rehabilitation.
- Identify the rate of adverse events and the safety level of the protocol evaluated in this clinical trial;

# MATERIAL AND METHOD

## Study Design

This is a clinical trial protocol, controlled, randomized, single-blind with two groups, an allocation ratio of 1:1, and a hypothesis of superiority that will be carried out with patients admitted to the adult Intensive Care Unit (ICU) of the University Hospital Onofre Lopes (HUOL) with the clinical diagnosis of acute myocardial infarction. Figure 1 represents the follow-up flowchart of the study according to CONSORT (Consolidated Standards of Reporting Trials) (49). Figure 2 presents the flowchart with the main stages of the study.

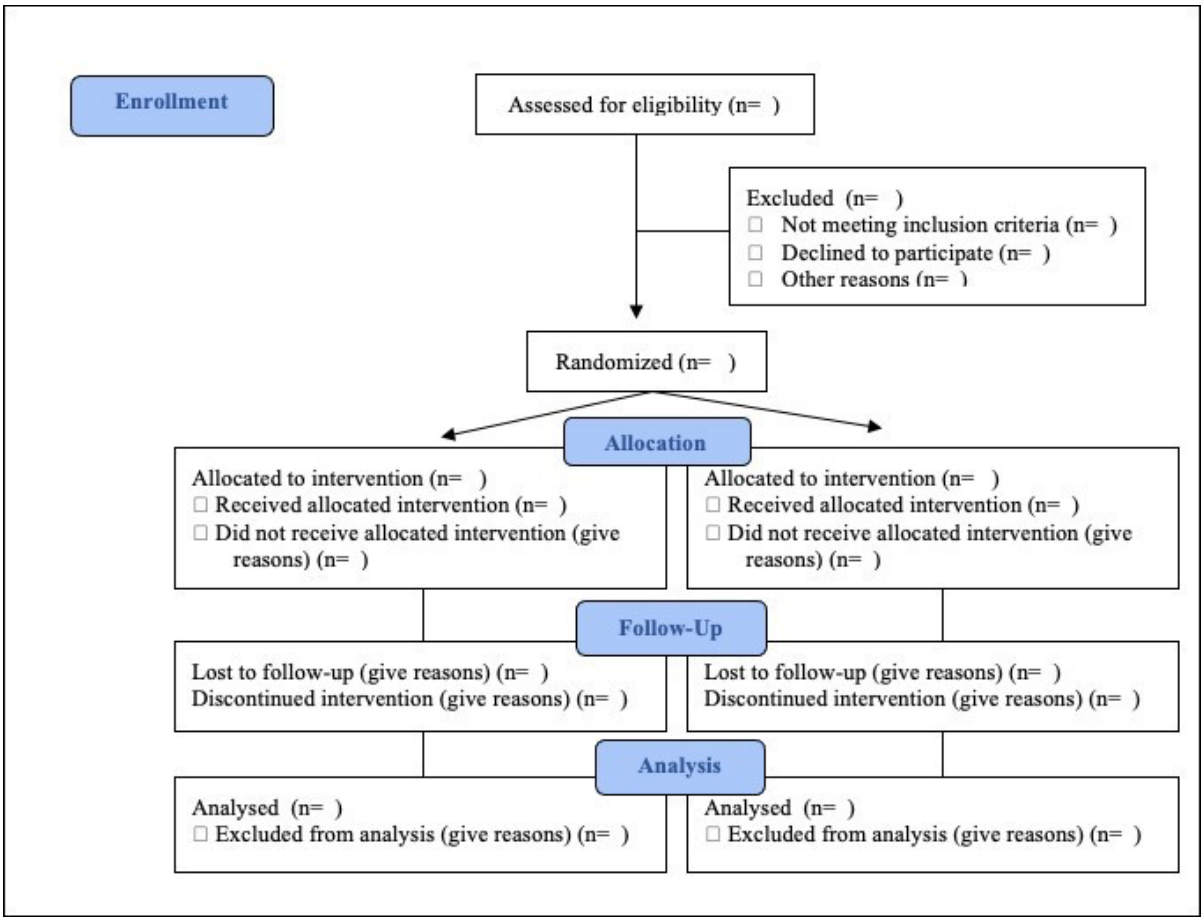


**Fig. 1** – Study flowchart according to the Consolidated Standards of Reporting Trials (CONSORT).

## Study design

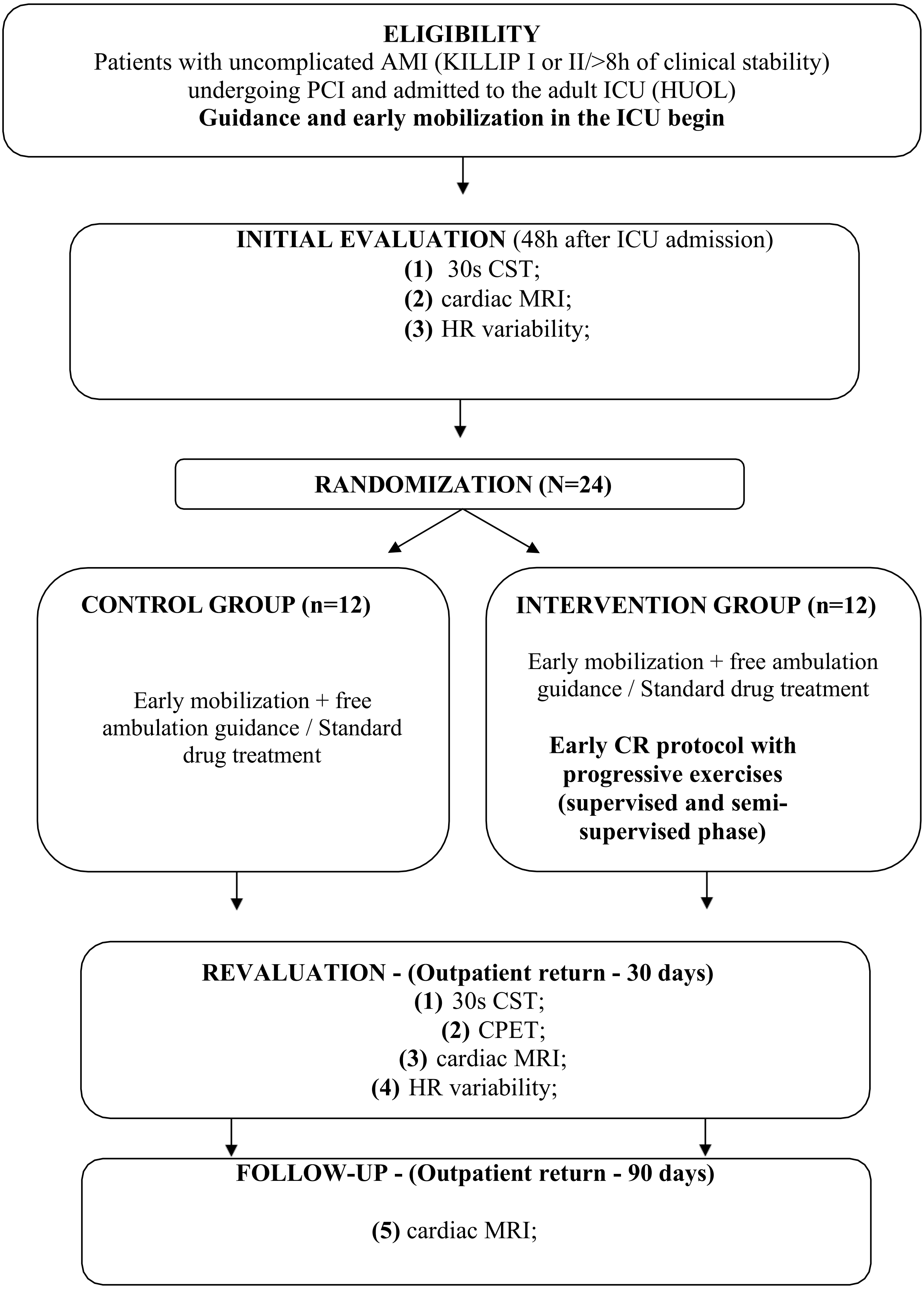


**Fig. 2** – Flowchart with specification of the evaluations and interventions carried out.

**Styding setting**

This study will be developed at the University Hospital Onofre Lopes (HUOL), in the adult Intensive Care Unit (ICU), and in the clinical wards, located in the city of Natal/Rio Grande do Norte.

**Participants**

The patients included in the study will be patients admitted to the adult ICU at HUOL with a confirmed clinical diagnosis of AMI (ECG and cardiac enzymes) with or without ST elevation and uncomplicated (Killip I or II), undergoing percutaneous coronary intervention (PCI) with success (Timi flow 2 or 3), aged between 18 years and 70 years and of both sexes, classified as low risk (EF>50%, absence of ventricular dysrhythmias, absence of clinical depression and ischemic signs or symptoms after the procedure) according to the American Association of Cardiovascular and Pulmonary Rehabilitation (AACPR) risk classification (50) and ability to provide informed consent. Exclusion criteria determined include the presence of musculoskeletal disorders that prevent the performance of exercises, signs, and symptoms of cardiac ischemia or decompensation, atrial or ventricular arrhythmias, and diagnosed pulmonary diseases (e.g., pulmonary hypertension, COPD, among others).

## Sampling and Recruitment

Participants will be recruited from the adult intensive care unit (ICU) shortly after admission for a percutaneous coronary intervention (PCI) procedure. The sample size was calculated using the OpenEpi software through the mean difference found in a previous study (29) with in-hospital CR for analysis of maximum oxygen consumption (VO2). A number of 7 participants per group was verified with a total of 14 for the sample considering a significance level of 5%, and a statistical power of 80%. Adding a 30% rate for possible losses during the study, we chose to recruit 12 participants per group for a total sample size of 24 patients. The patients included in the study will be patients admitted to the adult ICU at HUOL with a confirmed clinical diagnosis of AMI (ECG and cardiac enzymes) with or without ST elevation and uncomplicated (Killip I or II), undergoing percutaneous coronary intervention (PCI) with success (Timi flow 2 or 3), aged between 18 years and 70 years and of both sexes, classified as low risk (EF>50%, absence of ventricular dysrhythmias, absence of clinical depression and ischemic signs or symptoms after the procedure) according to the American Association of Cardiovascular and Pulmonary Rehabilitation (AACPR) risk classification (50) and ability to provide informed consent. Exclusion criteria determined include the presence of musculoskeletal disorders that prevent the performance of exercises, signs, and symptoms of cardiac ischemia or decompensation, atrial or ventricular arrhythmias, and diagnosed pulmonary diseases (e.g., pulmonary hypertension, COPD, among others).

## Randomization and blinding

The randomization sequence will be generated by software available online at www.randomizer.org by a study collaborator not involved in the processes of collecting or evaluating the results. The software will generate a sequence of uniformly random numbers for 2 groups. The sequence generated by the program will be placed in opaque envelopes and numbered sequentially. The opaque and sealed envelopes will only be under the domain of the researcher responsible for the randomization. Only the collaborators in charge of administering the intervention protocol will be aware of each participant's allocation group. Furthermore, all

outcome assessors will remain blinded during the study to participant allocation.

## Early cardiac rehabilitation program

The early cardiac rehabilitation (CR) program based on physical exercise will be carried out in 3 steps (Step 1 - Functional mobilization; Step 2 - Early cardiac rehabilitation; Step 3 - Continued cardiac rehabilitation).

In step 1 of early mobilization, all patients in the control and intervention groups, after 8 hours of clinical stability (absence of ischemic signs and symptoms) will perform mobility increment exercises to ensure functional mobility until discharge from the ICU, as per step 1 of the in-hospital CR protocol in (Appendix B). After ensuring functional mobility, patients will be randomized to control or intervention groups. Both groups will perform steps 1 to STEP 3 of the early CR protocol, witch the physical activity is a free-demand ambulation in addition to standardized drug treatment, however, only the intervention group will proceed to STEP 4 of steps 1 and steps 2 and 3 from early and continued cardiac rehabilitation.

In step 2, patients will follow early exercise-based cardiac rehabilitation performed following all prescription parameters (FITT-VP) and safety criteria recommended by ACSM, 2018 (33). The institutional protocol **(Appendix B)** will be performed with a **frequency** of 2 sessions per day, during the entire hospitalization, with an average of 8 to 10 total sessions; Low **intensity** for aerobic training with METs estimated by the subjective perception of effort of BORG between 9 to 12 reaching between 2 to 3 METs and for resistance training with 2 to 3 series of 60% of the maximum repetitions reached in the 30s sit-to-stand test; **Type** of exercises involved will be aerobic training using the cycle ergometer associated with resistance training using the body's own weight with functional sit-to-stand training; Aerobic training **time** ranging from 3 to 20 minutes. The **Volume or Dose** of aerobic training will be measured by calculating the training dose per session and the total dose applied during hospitalization, providing a value in time x intensity (METs) x frequency of accumulated sessions and total number of sessions x time. The structured **progression** in the protocol focuses on the time of aerobic exercise and the number of series of resistance training. The exercise sessions will follow the protocol presented, however, present individualized prescriptions based on tolerance time during aerobic training with a cycle ergometer and resistance training from the maximum number of repetitions reached in the 30s TSL evaluated

individually before randomization.

Before hospital discharge, patients in the intervention group will receive an exercise guidance booklet (Appendix C) to perform step 3 of semi-supervised and continued cardiac rehabilitation with the exercise time reached at the end of in-hospital rehabilitation, frequency of 5 x per week and progression of 5 minutes each week within the first month after hospital discharge, according to tolerance and self-monitoring of intensity through perceived exertion using the Borg scale, maintained between 9 to 12, to ensure light exercise to moderate intensity. In this phase, patients in the intervention group will be monitored weekly by telephone and will receive a diary (Appendix C) to record the time of daily and weekly activity that they must deliver when investigating when they return for reassessment 30 days after hospital discharge.

All patients will be monitored before, during, and after each supervised care session with a frequency meter and pulse oximeter (Model Nonin Onyx 9500, Nonin Medical, USA and Polar H10), which will allow the assessment of heart rate and O2 saturation. Blood pressure will be evaluated before and after the session and during the ICU stay the electrocardiogram will be monitored throughout the exercise. Patients will be instructed to interrupt the exercise in case of malaise (dyspnea, excessive sweating, cyanosis, use of accessory muscles, vertigo, retching), chest pain, or borg greater than 13 for both dyspnea and muscle fatigue.

## Data collection procedures

The participants will be evaluated at the following times: The first evaluation will be performed after 48 hours of admission to the ICU. The second assessment will take place from the 30th day after hospital discharge during the outpatient reassessment and the third assessment will take place at 90 days after hospital discharge to repeat MRI. The evaluative tests performed in each of the stages are described in the timeline described in Figure 3.

Anamnesis and clinical-physical evaluation

Initially, an interview will be held with the volunteer, where the clinical-physical evaluation form will be filled out (Appendix A), containing data regarding the identification of participants, personal and family pathological history, complaints, lifestyle habits, medications used, previous procedures, admission exams (Biomarkers, Electrocardiogram-ECG, Transthoracic echocardiogram-TEE, and Heart Catheterization- HEART CATH); In addition to physical assessment with data collection of vital signs and anthropometric data.

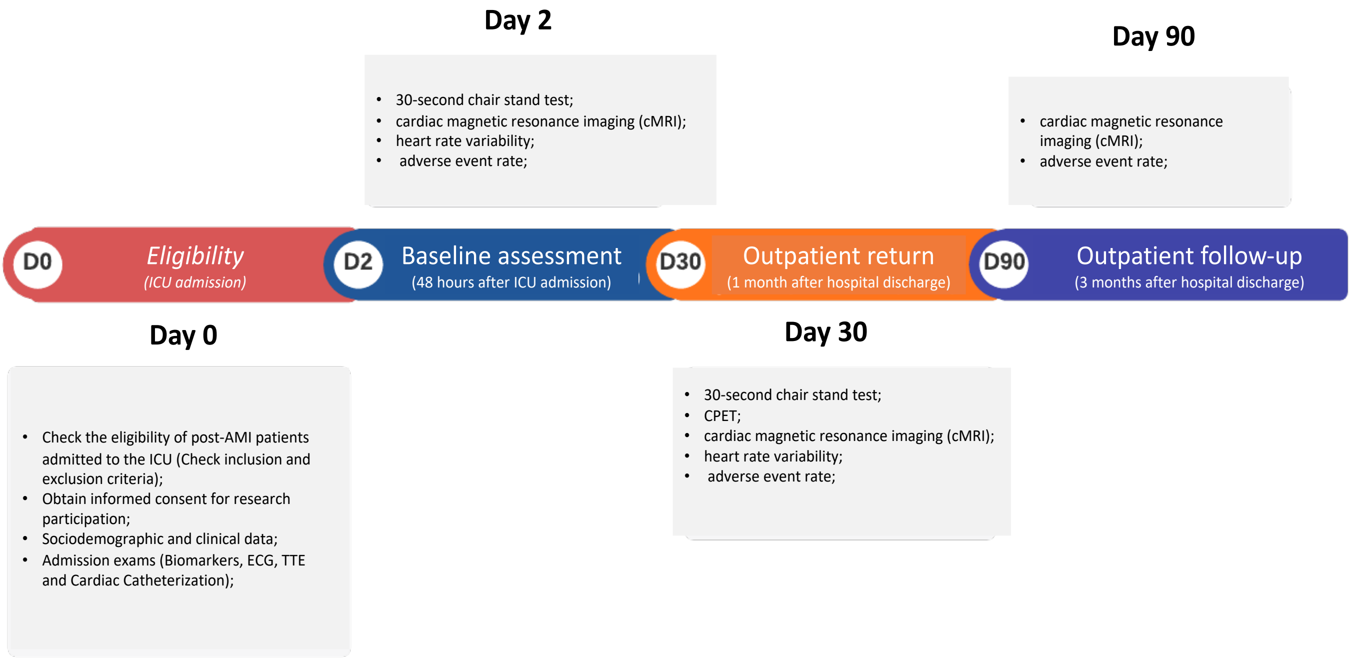


**Fig. 3** – Timeline of assessments

Physical Fitness analysis

*Cardiopulmonary exercise test (CPET)*

The maximum incremental test of the type Cardiopulmonary Exertion Test (CPET) or Ergospirometric Test will be used to measure functional capacity through ACR (Analysed by VO2

value) after CR. Patients will perform CPET on a treadmill (Centurion 300 model, Micromed, Brazil) conducted under the incremental load protocol previously developed by Weber33 so that exhaustion occurs between 8 and 12 minutes through the ErgoPC Elite test system (Micromed, Brazil), and all will perform the same protocol (Ramp). As part of the test, patients will be monitored by a 12-lead electrocardiogram at rest (Digital ECG, Micromed, Brazil), (SpO2) will be monitored using a Nonin 2500 oximeter. subjective perception of exertion monitored, using the Borg scale (51) as an instrument. All procedures will be performed under medical supervision and patients will be authorized to perform the test while leaning on the front support bars of the treadmill.

The metabolic gas analyzer Cortex Metamax 3B (Germany) and the Metalyzer 3B software will be used to capture, analyze, and interpret expired gases. The system consists of breath-by-breath monitoring, using a silicone mask with a 45ml dead space, where a volume turbine is coupled to measure minute volume (VE), and a gas line connected to an oxygen and oxygen cell. carbon dioxide for reading VO2, and VCO2, respectively. The system allows the transmission of data to the software, allowing the real-time monitoring of VO2 measurements relative to body mass (VO2/Kg), minute ventilation (VE), respiratory exchange rate, the ventilatory equivalent of carbon dioxide ( VE/VCO2) and oxygen (VE/VO2), and the oxygen pulse. The gas analyzer will be calibrated daily. The silicone mask with the turbine and gas line will be attached to the volunteer before the start of the test, and a period of two minutes will be respected allowing adaptation to the mask, avoiding capture of hyperventilation measures. Then the participant will remain in an orthostatic position at rest for two minutes to measure the baseline VO2 and respect the VE, R, and VO2 suitable for starting the test. The ideal VE at rest to start the exercise is between 8 and 15L/min, the R between 0.75 and 0.85, and the VO2RESO close to 3.5mL/kg/min, corresponding to 1 MET.

Participants will be instructed to perform the test until such time as they feel unable to continue. Throughout the test, cardiorespiratory and metabolic measurements will be recorded. The 12-lead electrocardiogram will be continuously monitored and will provide heart rate values. Blood pressure will be measured using a mercury column sphygmomanometer at two-minute intervals, peripheral oxygen saturation (SpO2) will be continuously monitored through the Nonin 2500 oximeter, and subjective exertion will be collected at two-minute intervals and at exhaustion (2). Criteria for stopping testing will be strictly followed according to the ATS 2003

standardization (52). The CPETs will be performed at the Cardiac Rehabilitation Unit of the Hospital Universitário Onofre Lopes (CORE-HUOL) (Figure 4) which has controlled room temperature and equipment and medications needed to provide emergency care. The silicone mask with the turbine and gas line will be attached to the volunteer before the start of the test, and a period of two minutes will be respected allowing adaptation to the mask, avoiding capture of hyperventilation measures. Then the participant will remain in an orthostatic position at rest for two minutes to measure the baseline VO2 and respect the VE, R, and VO2 suitable for starting the test. The ideal VE at rest to start the exercise is between 8 and 15L/min, the R between 0.75 and 0.85, and the VO2RESO close to 3.5mL/kg/min, corresponding to 1 MET.

After the end of the test, the patient remains monitored for five minutes to check the metabolic and cardiorespiratory behavior during recovery, with three minutes of active recovery and two minutes of passive recovery. After this period, new measurements of BP, SpO2, and Borg were performed.

If some of the tests need to be interrupted due to patient discomfort (fatigue, dyspnea, dizziness), the patient will receive the necessary care for his recovery. On the days when the maximum effort tests are carried out, patients will be assisted by a cardiologist in a hospital environment and, if necessary, appropriate support will be offered to the patient, such as a 5 to 10-minute rest, monitoring of vital signs and even referral to a sector of the hospital with oxygen therapy and greater therapeutic support, according to the clinical evaluation carried out by the physician present.

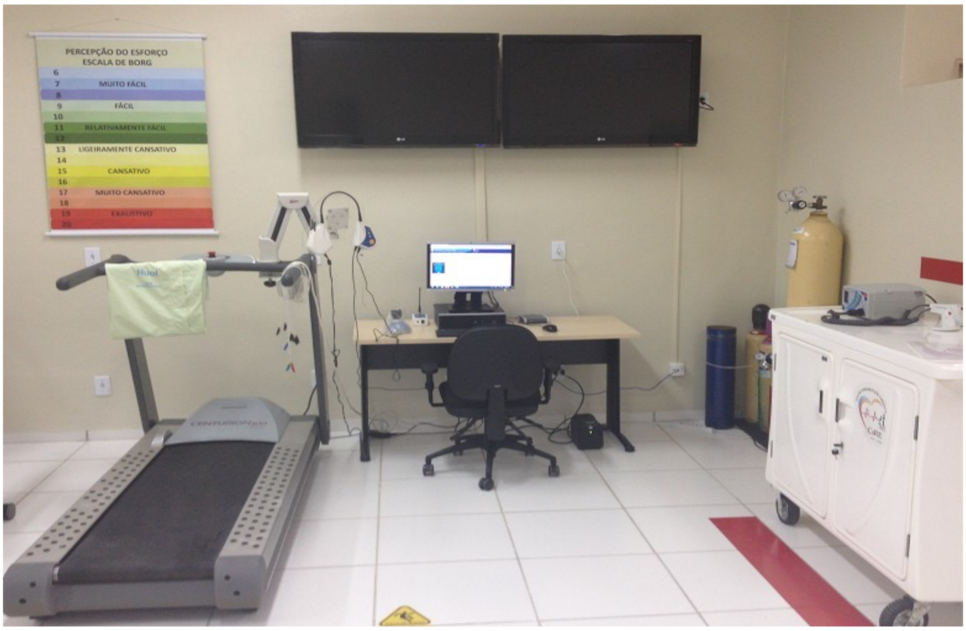


**Fig. 4 – Cardiact rehabilitation unit – CPET** (CORE-HUOL).

*30 second chair sit-to-stand test - 30CST*

The 30-second chair sit-to-stand test is a submaximal test indicated and validated for more debilitated individuals such as the elderly or hospitalized patients (30,31). In order to carry out the test, we will use, in accordance with previous studies and standardized recommendations, a rigid chair, with a standard height of 45cm and without armrests. The patient will be instructed to perform as quickly as possible in a time of 30 seconds the movement of sitting and standing up from the chair, which will be accounted for by the evaluator. The 30CST will be performed in 2 moments: At ICU discharge and 30 days after hospital discharge in both study groups.

The assessment of the intra- and inter-examiner reliability of the 30s TSL in post-AMI patients will be carried out from the recording of participants performing the TSL30s and evaluators previously selected to score the filmed tests. The total sample of 24 participants will be evaluated. The test will be administered by the principal investigator who will be blinded to the allocation of participants to study groups. Ten evaluators will be recruited from the hospital's team of physiotherapists from the ICU units and wards. All raters will be previously trained by the principal investigator before the assessments. After performing and filming the tests at ICU discharge (First assessment), the filmed tests will be presented to the evaluators who will score

each test. After 30 days, the same filmed tests will be presented again to the same evaluators for reassessment.

*Cardiac morphology and function analysis*

*Cardiac morphology*

Cardiac nuclear magnetic resonance (NMR) can be used for several important evaluations after acute myocardial infarction. MRI allows the diagnosis of myocardial infarction in its acute or chronic condition, provides several prognostic indicators that can impact the risk stratification of patients, in addition to allowing the identification of complications after AMI, such as aneurysm, pericarditis, and thrombi among others (53). The evaluation of the extent of scarring after AMI is a valuable predictor of therapeutic success, being used as a common endpoint in clinical trials. The extent of infarction is inversely related to clinical prognosis and is a more sensitive predictor than mass and ventricular ejection fraction of ventricular tachycardia, ultimately predicting all-cause mortality and ejection fraction-independent survival (53).

In this study, standard protocols will be used to assess cardiac morphology and function, such as end-systolic and diastolic volumes and ventricular ejection fraction evaluated through the CINE SSFP sequence, in addition to the delayed enhancement sequence with gadolinium (53). The exam will be performed at baseline, still during hospital stay, around the fourth to sixth day after the AMI and in two follow-up moments, the first one from 4 weeks after the event, with a tolerance of up to 2 weeks for image and the second from 3 months after the event with a tolerance of two weeks.

The technique used to evaluate the scar extension will be the delayed enhancement with the administration of gadolinium chelate. The patient will receive the gadolinium chelate between 10 and 20 minutes before the exam. In the presence of scar tissue, there is an increase in the extracellular space in relation to the intracellular environment due to the rupture of the cardiomyocyte membranes, with greater gadolinium impregnation in this region, generating hyper-enhancement in the image (53). Scar size assessment will use the following semi-quantitative method: Visual scoring of 17 standardized areas, on a 5-point scale, where 0= no hyperenhancing, 1= 1-25%, 2= 26 – 50%, 3=51

– 75% and 4= 76 – 100% hyperenhancing. The total scar score is given by adding the

scores for each segment and dividing by 17 (53).

*Heart Rate Variability – HRV*

Heart rate variability (HRV) is an easy and non-invasive method to assess the integrity and functioning of the autonomic nervous system on heart rhythm. HRV can be observed by R-R intervals and its reduction is usually related to aging and worse health conditions (42,44,45).

In this research, the HRV will be measured before the randomization of the groups and at the outpatient return 30 days after hospital discharge using a Polar chest strap type heart rate monitor, model H10. The data provided by the heart rate monitor will be analyzed using Kubios HRV software. Variables of interest include those in the time domain: SDNN - standard deviation of all normal RR intervals recorded in a time interval, expressed in ms; pNN50 - represents the percentage of adjacent RR intervals with a duration difference greater than 50 ms. In addition to these variables, those in the frequency domain will be analyzed: High frequency component (HF) ranging from 0.15 to 0.4 Hz; Low frequency (LF) component, ranging from 0.04 to 0.15 Hz and the LF/HF ratio (45).

Analysis of aerobic exercise dose in cardiac rehabilitation

Despite the standardized goal for aerobic exercise dose, each patient will have an individual tolerance limit for the progression of exercise dose, because of this, each patient will have their exercise dose monitored each CR week. The calculation method for aerobic exercise dose will be performed by multiplying the value of the estimated training intensity in (MET-task metabolic rate) estimated by the borg, the session time, and the frequency of sessions during each week, determining the dose in METxmin-Week (48).

Cardiohemodinamic safy of early cardiac rehabilitation

The safety of the institutional protocol tested in this trial will occur through notifications

of adverse events during and after the CR sessions provided to patients in the intervention group. We will report adverse events session by session, listing the main ones: arrhythmias, chest pain, borg>13, drop in systolic blood pressure (SBP) above 10 mmHg, and cardiorespiratory arrest. Other events not previously listed will also be recorded in the patient follow-up and evaluation form as per Appendix A.

**Statistic Strategy**

The variables of interest will be analyzed using the software (Graph Pad Prism version 7.0) and initially evaluated for the normal distribution of data using the Shapiro-Wilk test considering n<30. In the case of data with normal distribution, they will be analyzed for the mean and standard deviation. Data without normal distribution will be analyzed for median and percentiles. Comparison analyses of the physical functional variables of the 30CST, MRI morphological variables, and HRV cardiac functional variables of the PRE-CR and POST-CR times of each group will be performed using the Wilcoxon test if the data were non-parametric and the paired t-test if the data were parametric. Comparison between the control and intervention groups for CPET will be performed using the independent t-test or the Mann-Whitney test, depending on the normality of the data. The reliability and validity of the 30CST will be analyzed using Spearman's correlation and the intra-examiner reliability coefficient. For all hypothesis tests, a significance of 5% probability (P<0.05) of error will be assigned.

**Ethical Aspects**

This study will be submitted to the Ethics and Research Committee of the University Hospital Onofre Lopes, as determined by Resolution nº 466/12 of the National Health Council. In addition, all participants will be guaranteed the provision of a free and informed consent form (Appendix D), the term of institutional authorization for the use of patient documents (Appendix E), and ensured, throughout the study, the maintenance of secrecy and anonymity of participants.

## RISKS AND BENEFITS

The risks that the patients involved in the research may present are those inherent to physical activity such as dyspnea, dizziness, fatigue, exhaustion, hypotension, muscle strains, or joint pain. However, the risks will be minimized through the following measures: the proposed exercise will occur within the safety criteria established by the American College of Sports Medicine; For cases of more serious intercurrences such as atrial and ventricular arrhythmias, cardiac decompensation or even cardiorespiratory arrest, the sector has material for cardiorespiratory resuscitation, as well as training of all professionals from the ICU and cardiology ward to manage possible intercurrences.

Regarding the semi-supervised phase, patients will only perform low-intensity exercises with which they will already be adapted during hospitalization, in addition to the large evaluation filter to which they will be submitted to be chosen through the risk stratification of the American Association of Cardiovascular and Pulmonary Rehabilitation (AACPR) (50). Finally, only low-risk patients will be eligible and will undergo semi-supervised follow-up after hospital discharge as recommended by the latest 2020 Brazilian Cardiovascular Rehabilitation Guideline (54).

Among the benefits of this research for the participants, we will have the availability of information about the disease, evaluation, and professional advice on functional capacity and cardiorespiratory fitness, with the supply of an individualized functional exercise prescription booklet after the CPET at the end of the study (Appendix F) with instructions regarding the performance and dose of the exercise, which will lead to a greater stimulus to efficient self-care, enabling better control of your disease, reducing the risk of hospital admissions and morbidity and mortality.

## RESULTS AND IMPACTS EXPECTED

It is expected to observe through this research a new model of prescription of aerobic exercise in early cardiac rehabilitation based on the target dose of exercise. In addition, it is expected to verify the magnitude of the clinical impact of carrying out this prescription model through the analysis of cardiorespiratory fitness, measures of submaximal functional capacity through the chair sit-to-stand test, and the morphological and functional parameters of the heart.

This research has an impact on the process of in-hospital cardiac rehabilitation and early follow-up since it outlines a new therapeutic strategy that could make the CR process more efficient in changing the functional capacity of patients and, therefore, in the clinical prognosis of these patients. Finally, it provides a model for calculating the intensity and dose of aerobic exercise, favoring its practical applicability in hospitalization units.

This work may also stimulate new lines of research since the analysis of the ideal aerobic exercise dose within CR still needs to be extensively studied in order to obtain a firm consensus about the therapeutic window to be used with patients with heart disease. It is also expected that this project will present all the results at a national and international level, as well as publish articles in journals with a good impact factor (>1), Qualis A1-A2. Finally, it is understood that the study will contribute to greater importance of the application of this type of rehabilitation within Cardiovascular Physiotherapy.

# TIMELINE

| Activity | Months (2022) | | | | | | | | | | | |
|---|---|---|---|---|---|---|---|---|---|---|---|---|
| | Jan | Feb | Mar | Apr | May | Jun | Jul | Aug | Sep | Oct | Nov | Dec |
| Literature review | X | X | X | X | X | X | X | X | X | X | X | X |
| Submission to CEP-HUOL | | | | | X | | | | | | | |
| Pilot study | | | | | | | X | | | | | |
| Data collect | | | | | | | X | X | X | X | X | X |
| Project qualification | | | | | | | | | | | | X |

| **Activity** | **Months (2023)** | | | | | | | | | | | |
|---|---|---|---|---|---|---|---|---|---|---|---|---|
| | **Jan** | **Feb** | **Mar** | **Apr** | **May** | **Jun** | **Jul** | **Aug** | **Sep** | **Oct** | **Nov** | **Dec** |
| **Literature review** | **X** | **X** | **X** | **X** | **X** | **X** | **X** | **X** | **X** | **X** | **X** | **X** |
| **Discussion of the results found** | **X** | **X** | **X** | **X** | | | | | | | | |
| **Article submission** | | | | | **X** | **X** | **X** | | | | | |
| **PhD defense** | | | | | | | | **X** | | | | |

# BUDGET ESTIMATED (formulary according to the local ethical committee)

| **Permanent Material** | | | |
|---|---|---|---|
| Item | Unit price | Quantity | Total **(R$)** |
| Treadmil (Centurion 300) | 995,00 | 1 | 995,00 |
| Stethoscopy (Littmam) | 500,00 | 1 | 500,00 |
| Pulse Oxymeter (Nonin Onyx- 9500) | 300,00 | 1 | 300,00 |
| Chest strap heart frequency meter (H10 – POLAR) | 500,00 | 2 | 1.000,00 |
| Notebo"k 14" DELL inspiron 14-2630 preto core I3 | 2.400,00 | 1 | 2.400,00 |
| **Subtotal** | | | **5.195,00** |

| **Material de consumo**<br>**No-permanent** | | | |
|---|---|---|---|
| Item | Unit price | Quantity | Total **(R$)** |
| Toner Printer | 45,00 | 1 | 90,00 |
| paper A4 | 15,00 | 3 | 45,00 |
| Pencil (box) | 10,00 1cx | 1cx | 10,00 |
| Plastic folder | 5,00 | 3 | 15,00 |
| Eletcrodes for ECG (Meditrace) | 40,00 cx | 10 | 400,00 |
| Paper envelopes | 73,00 cx | 1 | 73,00 |
| **Subtotal** | | | **633,00** |

Resources spent on consumables and permanent materials amount to R$ 5,828.00. Part of these expenses, referring to permanent materials, are institutional assets and are available for research. The rest of the amount will be entirely the responsibility of the researcher. In addition to permanent and consumable materials, the study will require the use of the sector's telephone (CORE-HUOL) to make calls to patients in the semi-supervised rehabilitation phase and call for reassessment.

# REFERENCES


1. 2020 ESC Guidelines for the management of acute coronary syndromes in patients presenting without persistent ST-segment elevation | European Heart Journal | Oxford Academic [Internet]. [citado 29 de abril de 2021]. Disponível em: https://academic.oup.com/eurheartj/article/42/14/1289/5898842
2. Meneghelo RS, Araújo CGS, Stein R, Mastrocolla LE, Albuquerque PF, Serra SM. III Diretrizes da Sociedade Brasileira de Cardiologia sobre teste ergométrico. Arq Bras Cardiol. 2010;95(5):1–26.
3. Ibanez B, James S, Agewall S, Antunes MJ, Bucciarelli-Ducci C, Bueno H, et al. 2017 ESC Guidelines for the management of acute myocardial infarction in patients presenting with ST-segment elevation. Eur Heart J. 7 de janeiro de 2018;39(2):119–77.
4. Townsend N, Wilson L, Bhatnagar P, Wickramasinghe K, Rayner M, Nichols M. Cardiovascular disease in Europe: epidemiological update 2016. Eur Heart J. 7 de novembro de 2016;37(42):3232–45.
5. Virani Salim S., Alonso Alvaro, Aparicio Hugo J., Benjamin Emelia J., Bittencourt Marcio S., Callaway Clifton W., et al. Heart Disease and Stroke Statistics—2021 Update. Circulation. 23 de fevereiro de 2021;143(8):e254–743.
6. Anderson L, Taylor RS. Cardiac rehabilitation for people with heart disease: an overview of Cochrane systematic reviews. Cochrane Database Syst Rev. 12 de dezembro de 2014;(12):CD011273.
7. L A, Dr T, N O, Ad Z, K R, N M, et al. Exercise-based cardiac rehabilitation for coronary heart disease [Internet]. Vol. 2016, The Cochrane database of systematic reviews. Cochrane Database Syst Rev; 2016 [citado 14 de outubro de 2020]. Disponível em: https://pubmed.ncbi.nlm.nih.gov/26730878/
8. Amsterdam EA, Wenger NK, Brindis RG, Casey DE, Ganiats TG, Holmes DR, et al. 2014 AHA/ACC guideline for the management of patients with non-ST-elevation acute coronary syndromes: executive summary: a report of the American College of Cardiology/American Heart Association Task Force on Practice Guidelines. Circulation. 23 de dezembro de 2014;130(25):2354–94.
9. Anderson L, Oldridge N, Thompson DR, Zwisler A-D, Rees K, Martin N, et al. Exercise-Based Cardiac Rehabilitation for Coronary Heart Disease: Cochrane Systematic Review and Meta-Analysis. J Am Coll Cardiol. 5 de janeiro de 2016;67(1):1–12.
10. De Schutter A, Kachur S, Lavie CJ, Menezes A, Shum KK, Bangalore S, et al. Cardiac rehabilitation fitness changes and subsequent survival. Eur Heart J Qual Care Clin Outcomes. 1º de julho de 2018;4(3):173–9.
11. Borghi-Silva A, Mendes RG, Trimer R, Cipriano G. Current trends in reducing cardiovascular disease risk factors from around the world: focus on cardiac rehabilitation in Brazil. Prog Cardiovasc Dis. abril de 2014;56(5):536–42.
12. Rauch B, Davos CH, Doherty P, Saure D, Metzendorf M-I, Salzwedel A, et al. The prognostic effect of cardiac rehabilitation in the era of acute revascularisation and statin therapy: A systematic review and meta-analysis of randomized and non-randomized studies - The Cardiac Rehabilitation Outcome Study (CROS). Eur J Prev Cardiol. 2016;23(18):1914–39.
13. van Halewijn G, Deckers J, Tay HY, van Domburg R, Kotseva K, Wood D. Lessons

from contemporary trials of cardiovascular prevention and rehabilitation: A systematic review and meta-analysis. Int J Cardiol. 1º de abril de 2017;232:294–303.
14. Sandesara PB, Lambert CT, Gordon NF, Fletcher GF, Franklin BA, Wenger NK, et al. Cardiac rehabilitation and risk reduction: time to "rebrand and reinvigorate". J Am Coll Cardiol. 3 de fevereiro de 2015;65(4):389–95.
15. Lavie CJ, Arena R, Franklin BA. Cardiac Rehabilitation and Healthy Life-Style Interventions: Rectifying Program Deficiencies to Improve Patient Outcomes. J Am Coll Cardiol. 5 de janeiro de 2016;67(1):13–5.
16. Menezes AR, Lavie CJ, Milani RV, Forman DE, King M, Williams MA. Cardiac rehabilitation in the United States. Prog Cardiovasc Dis. abril de 2014;56(5):522–9.
17. Menezes AR, Lavie CJ, Forman DE, Arena R, Milani RV, Franklin BA. Cardiac rehabilitation in the elderly. Prog Cardiovasc Dis. outubro de 2014;57(2):152–9.
18. Grace SL, Bennett S, Ardern CI, Clark AM. Cardiac rehabilitation series: Canada. Prog Cardiovasc Dis. abril de 2014;56(5):530–5.
19. Humphrey R, Guazzi M, Niebauer J. Cardiac rehabilitation in Europe. Prog Cardiovasc Dis. abril de 2014;56(5):551–6.
20. Madan K, Babu AS, Contractor A, Sawhney JPS, Prabhakaran D, Gupta R. Cardiac rehabilitation in India. Prog Cardiovasc Dis. abril de 2014;56(5):543–50.
21. O'Connor GT, Buring JE, Yusuf S, Goldhaber SZ, Olmstead EM, Paffenbarger RS, et al. An overview of randomized trials of rehabilitation with exercise after myocardial infarction. Circulation. agosto de 1989;80(2):234–44.
22. Munir H, Fromowitz J, Goldfarb M. Early mobilization post-myocardial infarction: A scoping review. PLoS ONE [Internet]. 17 de agosto de 2020 [citado 30 de abril de 2021];15(8). Disponível em: https://www.ncbi.nlm.nih.gov/pmc/articles/PMC7430744/
23. Lavie CJ, Thomas RJ, Squires RW, Allison TG, Milani RV. Exercise training and cardiac rehabilitation in primary and secondary prevention of coronary heart disease. Mayo Clin Proc. abril de 2009;84(4):373–83.
24. Leon AS, Franklin BA, Costa F, Balady GJ, Berra KA, Stewart KJ, et al. Cardiac rehabilitation and secondary prevention of coronary heart disease: an American Heart Association scientific statement from the Council on Clinical Cardiology (Subcommittee on Exercise, Cardiac Rehabilitation, and Prevention) and the Council on Nutrition, Physical Activity, and Metabolism (Subcommittee on Physical Activity), in collaboration with the American association of Cardiovascular and Pulmonary Rehabilitation. Circulation. 25 de janeiro de 2005;111(3):369–76.
25. Martin B-J, Arena R, Haykowsky M, Hauer T, Austford LD, Knudtson M, et al. Cardiovascular fitness and mortality after contemporary cardiac rehabilitation. Mayo Clin Proc. maio de 2013;88(5):455–63.
26. Nogueira IDB, Servantes DM, Nogueira PA de MS, Pelcerman A, Salvetti XM, Salles F, et al. Correlação entre qualidade de vida e capacidade funcional na insuficiência cardíaca. Arq Bras Cardiol. agosto de 2010;95(2):238–43.
27. Smith PJ, Sherwood A, Mabe S, Watkins L, Hinderliter A, Blumenthal JA. Physical activity and psychosocial function following cardiac rehabilitation: One-year follow-up of the ENHANCED study. Gen Hosp Psychiatry. 2017;49:32–6.
28. Peixoto TCA, Begot I, Bolzan DW, Machado L, Reis MS, Papa V, et al. Early exercise-based rehabilitation improves health-related quality of life and functional capacity after acute myocardial infarction: a randomized controlled trial. Can J Cardiol. março de 2015;31(3):308–13.
29. Zanini M, Nery RM, de Lima JB, Buhler RP, da Silveira AD, Stein R. Effects of Different Rehabilitation Protocols in Inpatient Cardiac Rehabilitation After Coronary Artery

Bypass Graft Surgery: A RANDOMIZED CLINICAL TRIAL. J Cardiopulm Rehabil Prev. novembro de 2019;39(6):E19–25.
30. McAllister LS, Palombaro KM. Modified 30-Second Sit-to-Stand Test: Reliability and Validity in Older Adults Unable to Complete Traditional Sit-to-Stand Testing. J Geriatr Phys Ther 2001. setembro de 2020;43(3):153–8.
31. Zanini A, Crisafulli E, D'Andria M, Gregorini C, Cherubino F, Zampogna E, et al. Minimum Clinically Important Difference in 30-s Sit-to-Stand Test After Pulmonary Rehabilitation in Subjects With COPD. Respir Care. 1º de outubro de 2019;64(10):1261–9.
32. Arena Ross, Sietsema Kathy E. Cardiopulmonary Exercise Testing in the Clinical Evaluation of Patients With Heart and Lung Disease. Circulation. 15 de fevereiro de 2011;123(6):668–80.
33. American College of Sports Medicine. ACSM's Guidelines for Exercise Testing and Prescription. Vol. 10º. 2018.
34. Bellenger NG, Rajappan K, Rahman SL, Lahiri A, Raval U, Webster J, et al. Effects of carvedilol on left ventricular remodelling in chronic stable heart failure: a cardiovascular magnetic resonance study. Heart Br Card Soc. julho de 2004;90(7):760–4.
35. Giallauria F, Cirillo P, Lucci R, Pacileo M, De Lorenzo A, D'Agostino M, et al. Left ventricular remodelling in patients with moderate systolic dysfunction after myocardial infarction: favourable effects of exercise training and predictive role of N-terminal pro-brain natriuretic peptide. Eur J Cardiovasc Prev Rehabil Off J Eur Soc Cardiol Work Groups Epidemiol Prev Card Rehabil Exerc Physiol. fevereiro de 2008;15(1):113–8.
36. Wisløff U, Støylen A, Loennechen JP, Bruvold M, Rognmo Ø, Haram PM, et al. Superior cardiovascular effect of aerobic interval training versus moderate continuous training in heart failure patients: a randomized study. Circulation. 19 de junho de 2007;115(24):3086–94.
37. Jugdutt BI, Michorowski BL, Kappagoda CT. Exercise training after anterior Q wave myocardial infarction: importance of regional left ventricular function and topography. J Am Coll Cardiol. agosto de 1988;12(2):362–72.
38. Kubo N, Ohmura N, Nakada I, Yasu T, Katsuki T, Fujii M, et al. Exercise at ventilatory threshold aggravates left ventricular remodeling in patients with extensive anterior acute myocardial infarction. Am Heart J. janeiro de 2004;147(1):113–20.
39. Adachi H, Koike A, Obayashi T, Umezawa S, Aonuma K, Inada M, et al. Does appropriate endurance exercise training improve cardiac function in patients with prior myocardial infarction? Eur Heart J. outubro de 1996;17(10):1511–21.
40. Otsuka Y, Takaki H, Okano Y, Satoh T, Aihara N, Matsumoto T, et al. Exercise training without ventricular remodeling in patients with moderate to severe left ventricular dysfunction early after acute myocardial infarction. Int J Cardiol. fevereiro de 2003;87(2–3):237–44.
41. Zhang Y-M, Lu Y, Tang Y, Yang D, Wu H-F, Bian Z-P, et al. The effects of different initiation time of exercise training on left ventricular remodeling and cardiopulmonary rehabilitation in patients with left ventricular dysfunction after myocardial infarction. Disabil Rehabil. 2016;38(3):268–76.
42. Brateanu A. Heart rate variability after myocardial infarction: what we know and what we still need to find out. Curr Med Res Opin. 2015;31(10):1855–60.
43. Wolf MM, Varigos GA, Hunt D, Sloman JG. Sinus arrhythmia in acute myocardial infarction. Med J Aust. 15 de julho de 1978;2(2):52–3.
44. Heart rate variability: standards of measurement, physiological interpretation and clinical use. Task Force of the European Society of Cardiology and the North American Society of Pacing and Electrophysiology. Circulation. 1º de março de 1996;93(5):1043–65.

45. Figueiredo T de G, de Souza HCM, Neves VR, do Rêgo Barros AEV, Dornelas de Andrade A de F, Brandão DC. Effects of physical exercise on the autonomic nervous system in patients with coronary artery disease: a systematic review. Expert Rev Cardiovasc Ther. novembro de 2020;18(11):749–59.
46. Kanazawa N, Iijima H, Fushimi K. In-hospital cardiac rehabilitation and clinical outcomes in patients with acute myocardial infarction after percutaneous coronary intervention: a retrospective cohort study. BMJ Open. 1º de setembro de 2020;10(9):e039096.
47. Keteyian SJ, Kerrigan DJ, Lewis B, Ehrman JK, Brawner CA. Exercise training workloads in cardiac rehabilitation are associated with clinical outcomes in patients with heart failure. Am Heart J. 10 de julho de 2018;204:76–82.
48. Squires RW, Kaminsky LA, Porcari JP, Ruff JE, Savage PD, Williams MA. Progression of Exercise Training in Early Outpatient Cardiac Rehabilitation: AN OFFICIAL STATEMENT FROM THE AMERICAN ASSOCIATION OF CARDIOVASCULAR AND PULMONARY REHABILITATION. J Cardiopulm Rehabil Prev. 2018;38(3):139–46.
49. Schulz KF, Altman DG, Moher D, the CONSORT Group. CONSORT 2010 Statement: updated guidelines for reporting parallel group randomised trials. BMC Med. 24 de março de 2010;8(1):18.
50. Williams MA. Exercise testing in cardiac rehabilitation. Exercise prescription and beyond. Cardiol Clin. agosto de 2001;19(3):415–31.
51. Borg GA. Psychophysical bases of perceived exertion. Med Sci Sports Exerc. 1982;14(5):377–81.
52. American Thoracic Society, American College of Chest Physicians. ATS/ACCP Statement on cardiopulmonary exercise testing. Am J Respir Crit Care Med. 15 de janeiro de 2003;167(2):211–77.
53. Rajiah P, Desai MY, Kwon D, Flamm SD. MR imaging of myocardial infarction. Radiogr Rev Publ Radiol Soc N Am Inc. outubro de 2013;33(5):1383–412.
54. Carvalho T de, Milani M, Ferraz AS, Silveira AD da, Herdy AH, Hossri CAC, et al. Diretriz Brasileira de Reabilitação Cardiovascular – 2020. Arq Bras Cardiol. 22 de maio de 2020;114(5):943–87.

# APPENDICES

## APPENDIX A - CLINICAL EVALUATION FORM

**FEDERAL UNIVERSITY OF RIO GRANDE DO NORTE**
**UNIVERSITY HOSPITAL ONOFRE LOPES**
**Physiotherapeutic Assessment**

Valuation Date:____/____/____ Revaluation:____/____/____

**IDENTIFICATION AND SOCIODEMOGRAPHIC**

**Name:**______________________________ **Medical Record Number:** ______________________________
**Date of birth:**____/____/____ **Age:**____________ **Telephone:**______________________________
**Address:**______________________________________________________________
**Sex:** □ M □ F **Weight:**____**Height**:___**Marital status:** □ Married □Single □Divorced □ Widower
**Education**: □ Illiterate □ 1st degree □ 2st degree □ 3st degree □ Complete □ Incomplete
**Profession:**______________________________________________________________

**CLINICAL DATA**

**Cardiovascular risk factors:** □ SAH □ DM □ Dyslipidemia □ Sedentary □ smoker □ Family history □ Overweight

**Cardiovascular medication POS AMI:** □ Beta blocker
□ ACE inhibitors
□ Diuretics
□ Statins
□ Antiplatelet agents
□ Hypoglycemic agents
□ Others

**STEMI:** □ Yes □ No Localization: □ Anterior □ Lower □ Side

**Angioplasty:** □ Stent Culprit artery:____________________________Killip:________Timi:__________________
Time to reperfusion:________________________CPK (peak):_______Troponin (peak):_________

**PHYSICAL EXAMINATION PER SESSION**

**Session 1:** Session start: HR:________bpm RF:______bpm $SpO_2$:_______% BP:_______mmHg
End of session: HR:_______bpm RF:______bpm $SpO_2$:_______% BP:_______mmHg
Security: □ Angina □ Arrhythmia______________□ Borg>=13 □ Fall of SBP>=10mmHg
□ CPA □ Others:______________ Vasoactive drug (Dose) □_____Devices:_________
__CR summary:____________________________________________________________

**Session 2:** Session start: HR:________bpm RF:______bpm $SpO_2$:_______% BP:________mmHg
End of session: HR:_______bpm RF:______bpm $SpO_2$:_______% BP:_______mmHg
Security: □ Angina □ Arrhythmia______________□ Borg>=13 □ Fall of SBP >=10mmHg
□ CPA □ Others:______________Vasoactive drug (Dose) □_____Devices:__________
__CR summary:____________________________________________________________

**Session 3**: Session start: HR:_________bpm RF:______bpm $SpO_2$:_______% BP:________mmHg
End of session: HR:_______bpm RF:______bpm $SpO_2$:_______% BP:________mmHg
Security: □ Angina □ Arrhythmia______________□ Borg>=13 □ Fall of SBP >=10mmHg
□ CPA □ Others:______________Vasoactive drug (Dose) □_____Devices:__________
__CR summary:____________________________________________________________

**Session 4:** Session start: HR:_________bpm RF:______bpm $SpO_2$:_______% BP:________mmHg
End of session: HR:_______bpm RF:______bpm $SpO_2$:_______% BP:________mmHg
Security: □ Angina □ Arrhythmia______________□ Borg>=13 □ Fall of SBP >=10mmHg
□ CPA □ Others:______________Vasoactive drug (Dose) □_____Devices:___________
__CR summary:_____________________________________________________________

**Session 5:** Session start: HR:_________bpm RF:______bpm $SpO_2$:_______% BP:________mmHg
End of session: HR:_______bpm RF:______bpm $SpO_2$:_______% BP:________mmHg
Security: □ Angina □ Arrhythmia______________□ Borg>=13 □ Fall of SBP >=10mmHg
□ CPA □ Others:______________Vasoactive drug (Dose) □_____Devices:___________
__CR summary:_____________________________________________________________

**Session 6:** Session start: HR:_________bpm RF:______bpm $SpO_2$:_______% BP:________mmHg
End of session: HR:_______bpm RF:______bpm $SpO_2$:_______% BP:________mmHg
Security: □ Angina □ Arrhythmia______________□ Borg>=13 □ Fall of SBP >=10mmHg
□ CPA □ Others:______________Vasoactive drug (Dose) □_____Devices:___________
__CR summary:_____________________________________________________________

**Session 7:** Session start: HR:_________bpm RF:______bpm $SpO_2$:_______% BP:________mmHg
End of session: HR:_______bpm RF:______ bpm $SpO_2$:______% BP:________mmHg
Security: □ Angina □ Arrhythmia______________□ Borg>=13 □ Fall of SBP >=10mmHg
□ CPA □ Others:______________Vasoactive drug (Dose) □_____Devices:___________
__CR summary:_____________________________________________________________

**Session 8:** Session start: HR:_________bpm RF:______bpm $SpO_2$:_______% BP:________mmHg
End of session: HR:_______bpm RF:______bpm $SpO_2$:_______% BP:________mmHg
Security: □ Angina □ Arrhythmia______________□ Borg>=13 □ Fall of SBP >=10mmHg
□ CPA □ Others:______________Vasoactive drug (Dose) □_____Devices:___________
__CR summary:_____________________________________________________________

**Surplus sessions:** Any adverse events? Which?__________________________________________

| COMPLEMENTARY EXAMS | |
|---|---|
| **Electrodiogram** (1): ______________________________________________<br>□ Regular rhythm □Atrial fibrillation □Pacemaker □RBBB □LBBB □ Others _________<br>**Echocardiogram** (1): EF_______PAP_________Others:_______________________ | Date:___/ /___<br><br>Date:___/ /___ |
| **X-RAY** (1):______________________________________________________ | Date:___/ /___ |
| **Cardiac catheterization** (1):___________________________________________<br>_____________________________________________________________ | Date:___/ /___ |
| **Cardiac resonance** (1):_______________________________________________ | Date:___/ /___ |
| **Cardiac resonance** (2):_______________________________________________ | Date:___/ /___ |
| **Heart rate variability** (1):____________________________________________ | Date:___/ /___ |
| **Heart rate variability** (2):____________________________________________ | Date:___/ /___ |

| PHYSICAL TESTS | |
|---|---|
| **30CST** (1): Result:________Adverse event:____________________________<br>Before the test: HR:_____bpm RF:____bpm $SpO_2$:_____% BP:________mmHg<br>End of test: HR:______bpm RF:____bpm $SpO_2$:_____% BP:________mmHg | Date:___/___/___ |
| **30CST** (1 + 30min): Result:________Adverse event:_____________________<br>Before the test: HR:___bpm RF:____bpm $SpO_2$:____% BP:________mmHg<br>End of test: HR:______bpm RF:____bpm $SpO_2$:____% BP:________mmHg | |
| **30CST** (2): Result:________Adverse event:____________________________<br>Before the test: HR:_____bpm RF:____bpm $SpO_2$:_____% BP:________mmHg<br>End of test: HR:______bpm RF:___bpm $SpO_2$:_____% BP:________mmHg | Date:___/___/___ |
| **30CST** (2 + 30min): Result:________Adverse event:_____________________<br>Before the test: HR:_____bpm RF:____bpm $SpO_2$:_____% BP:________mmHg<br>End of test: HR:______bpm RF:____bpm $SpO_2$:_____% BP:________mmHg | |
| **CPET (2)**: $VO_{2\ Peak}$:_____Test time: _________________________________<br>Derived variables:__________________________________________________ | Date:___/___/___ |

## APPENDIX B – CARDIAC REHABILITATION PROTOCOL BASED ON IN-HOSPITAL EXERCISE.

**Early CR Protocol – Step 1**

| STEPS | Clinical Assessment | Type of exercise | Time | Intensity | Dose |
|---|---|---|---|---|---|
| **0** | KILIP IV/III OU TIMI 0-1 | Passive sitting in bed | 2 hours | --- | --- |
| **1** | Absence of signs and symptoms of cardiac ischemia > 8 hours (ECG, ischemic markers, or precordial pain) or cardiac decompensation. | Aerobic: lower limb cycle ergometer in bed.<br><br>Upper and lower muscle endurance. (Shoulder flexion and abduction, quadriceps block, and triple flexion). | 3-5 min<br><br>1 x 10 | Borg 9-10 (2 METs) | 10MET -min |
| **2** | Absence or low dose of inotropes, vasodilators, and intravenous antiplatelet administration. | Aerobic: lower limb cycle ergometer on the armchair.<br><br>Upper and lower muscle endurance. (Shoulder flexion and abduction, quadriceps block, and triple flexion). | 6-10min2<br><br>x 10 | Borg 9-10 (2 METs) | 20MET -min |
| **3** | Maintenance of the clinical picture and conditions specified above. | Aerobic: lower limb cycle ergometer in armchair + walking (10meters). Muscular endurance: 30-second chair-stand test. | 9-15min | Borg 9-10 (2 METs) | 30MET -min |

| | | | | | |
|---|---|---|---|---|---|
| | | | | | |
| **4** | Maintenance of the clinical picture and conditions specified above. | Aerobic: lower limb cycle ergometer on armchair + walking (75 to 100 meters).<br><br>Muscular endurance: sitto stand exercise. | 9-15min<br><br>2 x 60% test repetitions | Borg 9-10 (2 METs) | 30MET-min |

Subtitle: The frequency of the STEPS is twice a day following the established order. The progression of the exercises focuses on the exercise time, following the order of the STEPS. The aim ofthe protocol is to achieve an accumulated 90 MET-min until ICU discharge.

## Early CR Protocol – Step 2

| STEPS | Avaliação Clínica | Tipo de exercício | Tempo | Intensidade | Dose |
|---|---|---|---|---|---|
| **5** | Maintenance of the clinical picture and conditions specified above. | Aerobic: Walking + stair training<br><br>Muscular endurance: sitto stand exercise. | 9-15min<br><br>2 x 60% test repetitions | Borg 11-12 (3 METs) | 45MET-min |
| **6** | Maintenance of the clinical picture and conditions specified above. | Aerobic: Walking + stair training<br><br>Muscular endurance: sitto stand exercise. | 9-15min<br><br>2 x 60% test repetitions | Borg 11-12 (3 METs) | 45MET-min |

| | | | | | |
|---|---|---|---|---|---|
| **7** | Maintenance of the clinical picture and conditions specified above. | Aerobic: Walking + stair training<br><br>Muscular endurance: sitto stand exercise. | 12-20min<br><br>3 x 60% test repetitions | Borg 11-12 (3 METs) | 60MET -min |
| **8** | Maintenance of the clinical picture and conditions specified above. | Aerobic: Walking + stair training<br><br>Muscular endurance: sitto stand exercise. | 12-20min<br><br>3 x 60% test repetitions | Borg 11-12 (3 METs) | 60MET -min |

Subtitle: The frequency of the STEPS is twice a day following the established order. The progression of the exercises focuses on time and intensity variables, following the order of the STEPS. The aim of the protocol is to guarantee 210 MET-min until discharge from the ward and 300 MET-min until hospital discharge, by the sum of the execution dose spent in ICU and ward.

**APPENDIX C - PRESCRIPTION AND FOLLOW-UP FORM FOR EARLY CR Step 3**

**Early CR Protocol – Step 3**

| STEPS | Type of exercise | Frequency | Time | Intensity | Dose | Weekly diary |
|---|---|---|---|---|---|---|
| **1** | Aerobic: Walking | 5 x week | 20-25min | Borg 11-12 (3 METs) | 300 MET.min-sem | F:___ T:________ BORG:____ Dose:______ |
| **2** | Aerobic: Walking | 5 x week | 25-30min | Borg 11-12 (3 METs) | 375 MET.min-sem | F:___ T:________ BORG:____ Dose:______ |
| **3** | Aerobic: Walking | 5 x week | 30-35min | Borg 11-12 (3 METs) | 450 MET.min-sem | F:___ T:________ BORG:____ Dose:_____ |
| **4** | Aerobic: Walking | 5 x week | 35-40min | Borg 11-12 (3 METs) | 525 MET.min-sem | F:___ T:________ BORG:____ Dose:_____ |

Subtitle: In step 3, the STEPS are equivalent to the training week.

# APPENDIX D – INFORMED CONSENT TERM

## INFORMED CONSENT TERM

You are being invited as a volunteer to participate in the research "EFFECTIVENESS OF EARLY CARDIAC REHABILITATION AFTER ACUTE MYOCARDIAL INFARCTION:

RANDOMIZED CLINICAL TRIAL". This study will analyze the results of cardiac rehabilitation, using the review of your medical records and two moments of evaluations with physical and imaging exams. This research is important to understand the effect of early cardiac rehabilitation on your physical capacity and heart function so that professionals can establish more effective approaches during this process.

For this study, we will adopt the following procedures: Initially, your data will be analyzed through your medical records; In the first phase, the participant will be invited to carry out an in-hospital rehabilitation protocol with daily sessions of about 3 to 20 minutes and evaluation through physical test through the movement of sitting and standing up from a chair standardized by 30 seconds and will have an MRI-type image of the heart, which lasts about 20 minutes, during your hospital stay. In the second stage, after discharge from the hospital, you will receive a booklet with exercise guidelines to perform at home with daily sessions of about 20 to 40 minutes, and 30 days after your procedure, you will again be invited to an outpatient consultation at the hospital to repeat the sit and stand test and perform a test on the treadmill with an average duration of 8 to 12 minutes and finally, according to prior appointment, a new image exam of your heart through cardiac resonance with the same duration as the first exam, about 20 minutes.

This research may bring benefits to your physical and emotional health and ability to carry out your activities of daily living through an individualized prescription of the most suitable and safe physical exercises to be performed during your routine, and may even reduce the time of your hospital stay and give you more security for your hospital discharge and quality of life. In addition, you will receive medical reports about the treadmill test and magnetic resonance imaging of your heart, which may contribute to a more effective clinical treatment with your cardiologist. Finally, your participation in this research will help in understanding the best rehabilitation strategy with exercises after infarction, facilitating the treatment of several other patients with the same case and favoring the publication of these results to professionals worldwide.



| Rubrica do Participante/Responsável legal: | Rubrica do Pesquisador: |
|---|---|
| | |

This survey may present possible risks: The loss or disclosure of your personal data; Tiredness, change in blood pressure, an increase in your heart rate, and signs of dizziness after exercise or physical testing. Possible risks will be avoided as much as possible, as we will be continuously monitoring your vital signs and will stop the test or training so that there is no risk to the participant, in addition to providing clinical support in the hospital's inpatient units. In addition, your personal data will be protected through good handling of the data using a computer exclusively for research and we will follow the rules of the General Data Protection Law number 13.709.

You will incur no cost to participate in this study, nor will you receive any financial benefit. You will be informed about the study in any aspect you wish and you will be free to participate or refuse to participate, being able to stop participating or withdraw your consent at any stage or moment of the research, without any prejudice to you. In case of any problem that you may have related to the research or even any doubt, you will be entitled to free assistance that will be provided by the researcher M.a. Caroline Ferreira Schon, phone (*55 84) 99900-0359. If you suffer any damage demonstrably resulting from this research, you will receive the necessary assistance for its repair. Your data will be confidential and disclosed only in congresses or scientific publications, with no disclosure of any information that could identify you. These data will be kept by the researcher for a period of 5 years.

If you have any questions about the ethics of this research, you should contact the research ethics committee of the Hospital Universitário Onofre Lopes, telephone: 3342-5003, address: Av. Nilo Peçanha, 620- Petrópolis-Espaço João Machado-1ºandar-Prédio Administrativo - CEP 59.012-300-Natal/RN, e-mail. This document has been printed in duplicate. One will stay with you and the other with the researcher in charge MS Caroline Ferreira Schon.

## INFORMED CONSENT

After being clarified about the objectives, importance, and way in which the data will be collected in this research, in addition to knowing the risks, discomforts, and benefits that it will bring to me and having become aware of all my rights, I agree to participate in the research ( EFFECTIVENESS OF EARLY CARDIAC REHABILITATION AFTER ACUTE MYOCARDIAL INFARCTION: CLINICAL TRIAL RANDOMIZED) and I authorize the disclosure of information provided by me in congresses and/or scientific publications as long as no data can identify me.

| Rubrica do Participante/Responsável legal: | Rubrica do Pesquisador: |
| --- | --- |
| | |

City, __/___/___.

______________________________________________

**Signature of participant**

As the researcher responsible for the study (EFFICIENCY OF EARLY CARDIAC REHABILITATION AFTER ACUTE MYOCARDIAL INFARCTION: CLINICAL TRIAL RANDOMIZED), I declare that I assume full responsibility for faithfully complying with the methodological procedures and rights that were clarified and assured to the participant in this study, as well as maintaining secrecy and confidentiality about the identity of the same.

I also declare that I am aware that in failing to comply with the commitment assumed herein, I will be infringing the rules and guidelines proposed by Resolution 466/12 of the National Health Council – NHC-Brazil, which regulates research involving human beings.

City, __/___/___.

______________________________________________

**MS Caroline Ferreira Schon**

Researcher Responsible

| Rubrica do Participante/Responsável legal: | Rubrica do Pesquisador: |
| --- | --- |
| | |

EMPRESA BRASILEIRA DE SERVIÇOS HOSPITALARES
HOSPITAL UNIVERSITÁRIO ONOFRE LOPES
GERÊNCIA DE ENSINO E PESQUISA

# TERM OF INSTITUTIONAL AUTHORIZATION FOR THE USE OF PATIENTS DOCUMENTS

**Ilmo. Prof. Dr. Carlos Alberto Almeida de Araújo**

**Manager of research and teaching of HUOL/EBSERH**

The Physiotherapy Department of the Federal University of Rio Grande do Norte will carry out research under the guidance of Prof. Dr. Selma Sousa Bruno entitled "EFFECTIVENESS OF EARLY CARDIAC REHABILITATION AFTER ACUTE MYOCARDIAL INFARCTION: RANDOMIZED CLINICAL TRIAL". This study addresses an analysis of the results of early cardiac rehabilitation (CR), using the review of medical records and assessments during the beginning of CR and within 90 days after the patient's hospital discharge. This research is important to understand the effect of early CR so that professionals can establish more effective approaches during CR.

Therefore, we request that you have valuable collaboration, in the sense of authorizing the access and use of the medical records and test results by the researcher in charge MS. Caroline Ferreira Schon, and professor Dr. Selma Sousa Bruno. We emphasize that the data collected will be kept confidential and used only for this study, minimizing the risk of patient exposure. Appropriate precautions will be taken to ensure that documents are not damaged; that is, the researchers undertake to handle the documents in a reserved environment intended for this and not to remove them from the place of origin, not to photograph or make copies of any nature. Even with the care taken by the researchers with the patients, and owners of the documents provided, in case of damage resulting from the research, they will be duly compensated by the researchers. This research presents the following benefits: the supply of a medical report about the exercise test that can help in better clinical control with your cardiologist and you will receive a personalized booklet with guidelines about the best and safest exercises to be performed during your routine according to the result of the test.

The data will be stored in a safe place at UFRN under the responsibility of the Research Coordinator Dr. Selma Sousa Bruno and the dissemination of results will be done in a way that does not identify the volunteers. The research will not incur expenses neither for this Institution - University Hospital Onofre Lopes/UFRN, nor for the research subjects.

However, if any unforeseen expenses arise, related to the participation of HUOL or the subjects, we undertake to reimburse such expenses, upon request. The Centre's participation is voluntary, which means that you may withdraw your consent at any time, without bringing any damage or penalty to the Institution/HUOL/UFRN or patients who own the documents. You will have a copy of this Term, prepared in two pages, and any questions you may have about this research, you can ask Ms. Caroline Ferreira Schon, the researcher responsible for this research, telephone: (84) 999000359, e-mail: caroline.schon@ebserh.gov.br or Prof. Dr. Selma Sousa Bruno, supervisor of this project in the Department of Physiotherapy; telephone: (*55 84) (999817854), or e-mail (Selma.bruno@ufrn.br).

Doubts regarding the ethics of this research may be asked to the Research Ethics Committee of HUOL, located at Av. Nilo Peçanha, 620, Petrópolis - Administrative Building - 1st floor – Espaço João Machado, Telephone (*55 84) 3342-5003.

I also declare that I am aware that in failing to comply with the commitment assumed herein, I will be infringing the rules and guidelines proposed by Resolution 466/12 of the National Health Council – NHC-Brazil, which regulates research involving human beings.

Counting on the understanding of this admnistration, we thank you in advance.

City,_____de__________2022.

**MA Caroline Ferreira Schon**

Researcher Responsible

( x ) We agree with the request ( ) We do not agree with the request

**Prof. Dr. Carlos Alberto Almeida de Araújo**

**Manager of research and teaching of HUOL/EBSERH**

## APPENDIX F - GUIDANCE BOOKLET FOR PHYSICAL EXERCISES

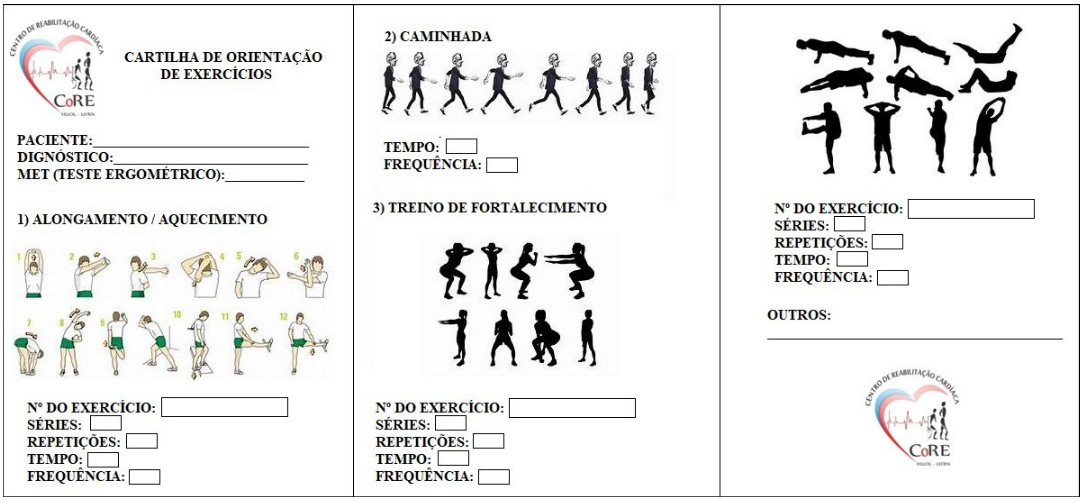


2/2

# ANNEXES

**ANNEX A - PERCEPTION OF EFFORT SCALE (BORG)**

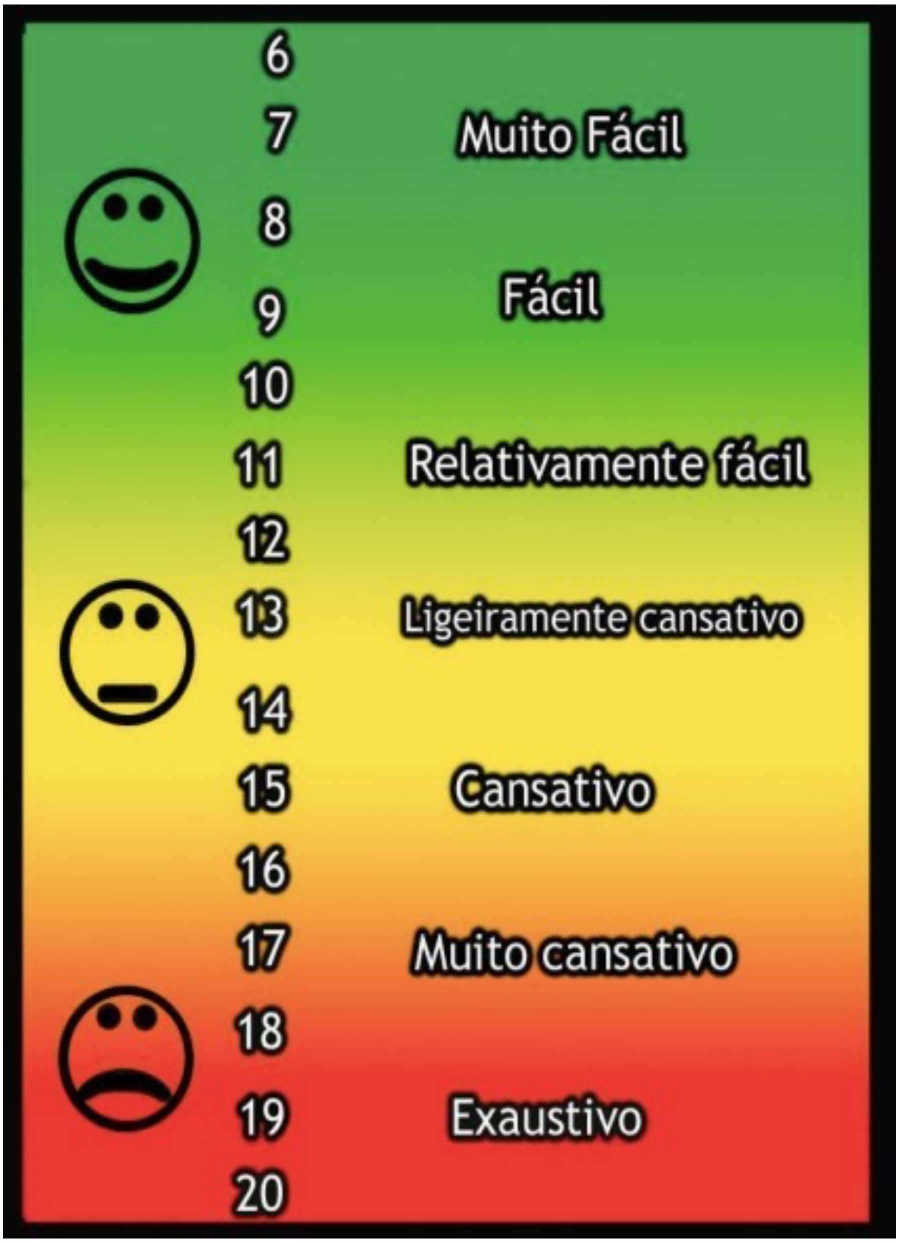

Supplement: S1 Protocol — (PDF) [file pone.0296345.s003.pdf]
