# Supplementary material for: Efficacy of early cardiac rehabilitation after acute myocardial infarction: Randomized clinical trial protocol
Source: PLoS One. 2024 Jan 10;19(1):e0296345. doi: 10.1371/journal.pone.0296345 (PMC10781044; doi:10.1371/journal.pone.0296345)

UNIVERSIDADE FEDERAL DO RIO GRANDE DO NORTE
PROGRAMA DE PÓS-GRADUAÇÃO EM FISIOTERAPIA

EFICÁCIA DA REABILITAÇÃO CARDÍACA PRECOCE APÓS INFARTO AGUDO DO MIOCÁRDIO: ENSAIO CLÍNICO RANDOMIZADO.

NATAL – RN
2022

UNIVERSIDADE FEDERAL DO RIO GRANDE DO NORTE
PROGRAMA DE PÓS-GRADUAÇÃO EM FISIOTERAPIA

CAROLINE FERREIRA SCHON

EFICÁCIA DA REABILITAÇÃO CARDÍACA PRECOCE APÓS INFARTO AGUDO DO MIOCÁRDIO: ENSAIO CLÍNICO RANDOMIZADO.

Projeto destinado à avaliação pela comissão de ética em pesquisa (CEP) do Hospital Universitário Onofre Lopes (HUOL).

Área de concentração: Avaliação e Intervenção em Fisioterapia.
Área de pesquisa: Avaliação e intervenção nos Sistemas Cardiovascular e Respiratório.
Orientadora: Prof[a]. Dra. Selma Sousa Bruno

NATAL – RN
2022

SUMÁRIO

1. **INTRODUÇÃO** 7

2. **OBJETIVOS** 15

2.1 Objetivo geral 15

2.2 Obetivos específicos 15

3. **MATERIAIS E MÉTODO** 16

3.1 Delineamento do estudo 16

3.2 Local da estudo 18

3.3 Participantes do estudo 18

3.4 Amostragem e recrutamento 19

3.5 Randomização e cegamento. 19

3.6 Protocolo de reabilitação cardíaca precoce baseada em exercício 20

3.5 Procedimentos de coleta de dados 21

3.51 Anamnese e avaliação clínico-física 22

3.52 Análise de Aptidão Física 22

1. Teste de Esforço Cardiopulmonar (TECP) 23
2. Teste de Senta-levanta de 30s (TSL) 25

3.53 Análise de Morfologia e Função Cardíaca 26

1. Ressonância nuclear magnética (RNM) 26
2. Variabilidade da frequência cardíaca (VFC) 27

3.54. Análise da Dose de Exercício Aeróbico na RC 28

3.55. Análise de Segurança Cardiohemodinâmica 28

3.6 Estratégia Estatística 28

3.7 Aspectos Éticos 29

4. **RISCOS E BENEFÍCIOS** 30

5 **RESULTADOS ESPERADOS** 31

6 **CRONOGRAMA** 32

7 **ORÇAMENTO** 34

8 **REFERÊNCIAS** 36

APÊNDICES

APÊNDICE A - Ficha de Avaliação Clínica 41
APÊNDICE B - Protocolo de RC Intra-hospitalar 44
APÊNDICE C - Ficha de Prescrição e Acompanhamento da RC Precoce 47
APÊNDICE D - Termo de Consentimento Livre e Esclarecido 48
APÊNDICE E -Termo de autorização institucional para uso de documentos dos pacientes 51
APÊNDICE F - Cartilha de Orientações para Exercícios Físicos 53

ANEXOS

ANEXO I - Escala de Percepção de Esforço (BORG) 55

RESUMO

A reabilitação cardíaca (RC) baseada em exercícios é um procedimento não medicamentoso já incorporado clinicamente com recomendação grau AI para pacientes pós infarto agudo do miocárdio (IAM) na fase ambulatorial. Entretanto, tais evidências derivam de estudos em fases mais tardia após o evento cardíaco. A RC na fase hospitalar e precoce ao evento têm crescido na última década, mas sua eficácia e segurança durante o procedimento precisam ser esclarecidas com desenhos metodológicos mais robustos. Assim, o objetivo deste estudo é analisar a eficácia de um protocolo de RC baseada em exercícios físicos sobre a aptidão física, morfologia e função cardíaca, além da segurança cardiohemodinâmica de pacientes internos com IAM recente. Será realizado um ensaio clínico controlado e randomizado, no Hospital Universitário Onofre Lopes (HUOL) com protocolo de RC baseada em exercício físico precoce (>8 horas de estabilidade clínica) de pacientes com IAM com sucesso na intervenção coronariana percutânea (ICP). Após aleatorização, pacientes serão agrupados em GI e GC (intervenção e controle), sendo o GI submetido a protocolo de baixa intensidade de RC padrão e o GC a cuidados e orientações usuais de mobilização. Os sujeitos em ambos os grupos serão submetidos as mesmas avalições funcionais, máxima e submáxima (testes cardiopulmonar e de senta-levanta de 30s), ressonância nuclear magnética cardíaca e a analisada a variabilidade da frequência cardíaca. Serão analisados escores de segurança e eventos cardiohemodinâmicos durante a reabilitação. Espera-se observar ao final do estudo efeito positivo da RC intra-hospitalar sobre a estrutura e função cardíaca e sua repercussão clínica no incremento da aptidão física, permitindo maior entendimento sobre as variáveis da prescrição do exercício e suas respostas fisiológicas para prever estratégias terapêuticas mais eficientes e precoces durante o processo de reabilitação intra-hospitalar em pacientes com IAM.

Palavras-chave: Reabilitação cardíaca precoce; Aptidão física; dose de exercício, Infarto agudo do miocárdio.

LISTA DE ABREVIATURAS

AACVPR *American Association of Cardiovascular and Pulmonary Rehabilitation*

ACR Aptidão Cardiorrespiratória

ACSM *American College of Sports in Medicine*

AHA *American Heart Association*

DCV Doença Cardiovascular

DPOC Doença Pulmonar Obstrutiva Crônica

FE Fração de Ejeção

HUOL Hospital Universitário Onofre Lopes

ICP Intervenção Coronária Percutânea

IAM Infarto Agudo do Miocárdio

MET Equivalente Metabólico da Tarefa

RC Reabilitação Cardíaca

RCR Remodelamento Cardíaco Reverso

RNM Ressonância Nuclear Magnética

RVM Revascularização do Miocárdio

SCA Síndrome Coronariana Aguda

TECP Teste de Esforço Cardiopulmonar

TSL Teste de Senta-Levanta

TC6m Teste de Caminhada de Seis Minutos

VFC Variabilidade da Frequência Cardíaca

INTRODUÇÃO

1.0 Síndrome Coronariana Aguda e Infarto Agudo do Miocárdio (IAM)

A síndrome coronariana aguda (SCA) ocorre por uma redução ou ausência súbita de fluxo sanguíneo nas artérias coronárias, acarretando, portanto, em déficit de perfusão do tecido cardíaco. A apresentação clínica da síndrome pode ser ampla e variar desde o sintoma típico de desconforto torácico agudo a instabilidades elétricas ou hemodinâmicas que podem evoluir para a parada cardiorrespiratória (1,2). O tratamento deve ser imediato e é preconizado nesta condição clínica, realizar a terapia de reperfusão por intervenção coronária percutânea (ICP) primária, no caso da não disponibilidade desta, a terapia fibrinolítica deverá estar indicada (1,3).

A morte dos cardiomiócitos secundária ao processo de ineficiência da perfusão pelas coronárias afetadas pode ser definida pelo termo infarto agudo do miocárdio (IAM). A necrose miocárdica pode ser verificada através de exames específicos sendo o mais recomendado a avaliação da troponina cardíaca de alta sensibilidade (hs-cTn) que define o IAM com sua elevação acima do percentil 99 dos valores de referência (3).

Embora existam variações entre os países europeus, a isquemia cardíaca é responsável por cerca de 1,8 milhões de mortes anuais (4). O mais recente Update da American Heart Association (AHA) de 2021 mostra uma prevalência de 49,2% (126,9 milhões) de doenças cardiovasculares (incluindo a doença cardíaca isquêmica) em adultos (5). Estudos atuais demonstram redução da mortalidade aguda e em longo prazo após IAM e correlacionam esse fato a maior utilização da terapia de reperfusão por ICP, apesar deste dado, a mortalidade após um evento isquêmico cardíaco contínua gerando óbitos substanciais com mortalidade intra-hospitalar de cerca de 13,6% e mortalidade em 1 ano de cerca de 10% (3,5).

2.0 Reabilitação Cardíaca (RC)

A mortalidade após IAM a longo prazo também pode ser influenciada positivamente por outra terapêutica que deve estar inclusa no manejo do paciente com cardiopatia isquêmica: A reabilitação cardíaca (RC). A RC é recomendada com alto nível de evidência (A) e forte classe de recomendação (I) baseada nas extensas revisões sistemáticas sobre o tema e devidamente recomendada pelos guias clínicos (3,6–8). A RC promove redução na mortalidade cardiovascular e por todas as causas, melhora a qualidade de vida e a capacidade funcional nesse perfil de pacientes (6,7). Em recente revisão sobre o tema , a Cochranne mostrou revisão de 14.486 pacientes com doença coronariana que participaram da RC, e mostrou significativa redução da mortalidade cardiovascular e hospitalização quando comparado a grupo controle (nãoparticipantes de RC) (9). Os desfechos clínicos acompanhados nos estudos de follow-up são associados principalmente à atenuação e melhor controle dos fatores de risco da doença cardiovascular (DCV) pela mudança do estilo de vida, incluindo um estilo de vida para indivíduosmais fisicamente ativo (10,11).

A reabilitação cardíaca é definida pelos guias clínicos mundiais como um processo de intervenção multifacetada que objetiva promover a recuperação integral (melhora físico-funcional como âncora dessa recuperação) de pacientes acometidos por eventos cardíacos agudos oudoenças cardiovasculares crônicas. O programa formal de RC deve ser considerado como fase essencial para todos os pacientes elegíveis, com somente alguns casos sendo recomendado apenas a orientação para exercícios domiciliares (9,12–21).

A programação da RC é tradicionalmente subdividida em etapas que obedecem uma lógica temporal, espacial e de nível de suporte clínico que o paciente necessita, de forma que, a primeira oferta da RC acontece na fase hospitalar que visa a mobilização e reabilitação precoce com exercícios de baixa intensidade, orientações ao paciente sobre sua doença e os fatores de risco modificáveis além das estratégias para adoção de um estilo de vida saudável tendo por objetivo propiciar alta hospitalar com melhor condição física e psíquica possível. Após a alta hospitalar o paciente deverá seguir para continuidade nas fases extra-hospitalar ou ambulatorial que ocorre imediatamente após a alta e é uma etapa prolongada que continua o processo de incremento da

capacidade física e provém da manutenção do hábito ativo como estilo de vida.

Apesar da ampla recomendação científica-clínica, ainda há um baixo número de encaminhamento e participação de pacientes na RC formal após a alta hospitalar o que aponta para um tratamento não farmacológico ainda subutilizado considerando a importância da relação necessidade clínica x controle da doença cardiovascular x baixo custo de procedimento. Dados da atualização da American Heart Association (AHA) alertam que apenas 24,4% dos pacientes com elegíveis para participar da RC, com média de 47 dias para início da reabilitação. Além disso, dos que participaram da RC, apenas 26,9% dos pacientes concluem o programa. Desses, cerca de até 93% são os pacientes que fizeram a reabilitação cardíaca precoce ainda na fase hospitalar. Esses dados mostram que é preciso um investimento maior na estruturação e viabilidade de locais de reabilitação cardíaca para o melhor controle da doença cardiovascular. Além disso, mostra também a necessidade de continuidade entre as fases hospitalar e ambulatorial a fim de otimizar um contínuo entre controle de futuros eventos cardíacos.

Tendo em vista os achados anteriores, uma questão que pode ser bastante relevante é a ausência do início precoce do processo de reabilitação na fase hospitalar, que pode ser um ponto chave na mudança da cultura da subvalorização da RC tanto por parte da equipe de profissionais cardiovasculares quanto pelos próprios pacientes. A última revisão de escopo sobre o tema verificou ainda que a maior parte do corpo de evidências científicas atuais são baseadas em estudos experimentais da era pré revascularização por ICP o que não reflete o contexto clínico atual, além disso, as diretrizes clínicas das sociedades cardiovasculares não fornecem recomendações específicas para a fase hospitalar da RC (22). Por fim, há uma lacuna no conhecimento científico sobre o tempo seguro para iniciar a RC, seus benefícios, segurança e parâmetros da prescrição no contexto intra-hospitalar.

3.0 Aptidão física após RC

O estado de sedentarismo já está apontado na literatura científica como fator de risco crucial para eventos cardíacos primários e secundários (23). Em correlação com este achado,

outros estudos demonstram o nível de aptidão cardiorrespiratória (ACR), componente da aptidão física, como o maior preditor de mortalidade comparado a qualquer outro fator de risco cardiovascular. A ACR vem sendo, portanto, avaliada em diversos estudos que buscam verificar os benefícios da RC, por sua notória modificação durante o tratamento. Trabalhos anteriores mostraram um ganho médio de 11 a 36% na ACR após RC tradicional em fase ambulatorial, com maiores ganhos nos pacientes que apresentaram menores níveis de ACR no início do programa (24). Dessa maneira há evidência suficiente para comprovar que as melhorias na ACR podem explicar a maior parte dos benefícios físicos, psicológicos e funcionais, assim como, os desfechos clínicos de mortalidades e hospitalização após realização de um programa formal de RC (10,25–27).

Entre os efeitos fisiológicos promovidos pelo componente físico da RC que podem justificar os ganhos da ACR e suas repercussões para o estado geral de saúde após o programa estão: Atenuação da gravidade da isquemia induzida pelo esforço com melhora da angina em repouso por elevação dos limiares isquêmicos e melhora da disfunção endotelial, aumento do consumo de oxigênio de pico e da potência aeróbica máxima com melhora na capacidade oxidativa do músculo esquelético, redução da exacerbação neuro-humoral, ação favorável sobre o perfil lipídico, principalmente nos casos de hipertrigliceridemia, níveis diminuídos de HDL-colesterol e alterações nas subfrações do LDL-colesterol, efeito hipotensor do exercício com redução dos níveis pressóricos sistólicos e diastólicos (9).

Na fase intra-hospitalar da RC alguns ensaios clínicos também demonstraram melhora em relação a componentes da aptidão física através de medidas indiretas ou estimados da ACR, tais como, incremento significativo da distância percorrida no teste de caminhada de seis minutos (TC6min) (28,29). Apesar disso, muitos serviços hospitalares não dispõem de estrutura física adequada para a realização do TC6min que exige um corredor plano e contínuo de 30 metros e mais de um avaliador na execução do teste. Outros testes submáximos de exercícios têm sido utilizados para avaliação funcional e estimativa dos componentes da aptidão física. Uma possibilidade menos dispendiosa em relação ao número de avaliadores e espaço físico é teste de senta-levanta de 30 segundos. O teste de sentar-levantar de 30s já está validado para a população de idosos e pacientes com Doença Pulmonar Obstrutiva Crônica (DPOC), apresentando inclusive

diferença clinicamente relevante estabelecida para acompanhamento de efeitos com intervenção de exercício (30,31). Nenhum ensaio clínico, até o momento, avaliou a validade e segurança deste teste funcional com pacientes internados após IAM.

A avaliação dos efeitos da RC é tradicionalmente verificado por meio de medida direta da ACR através de um teste de esforço cardiopulmonar obtendo-se a mensuração direta do pico de consumo de oxigênio ($VO_{2\ pico}$), método recomendado por diversas diretrizes internacionais (32,33). Apenas um ensaio clínico de fase hospitalar da RC (29) realizou o teste cardiopulmonar para avaliar o $VO_{2\ pico}$, 30 dias após a RC no perfil de pacientes após cirurgia de revascularização do miocárdio (RVM). Há, entretanto, grande escassez de análise direta da ACR em pacientes clínicos pós IAM submetidos a ICP.

4.0 Morfologia Cardíaca após RC

A despeito dos efeitos bem documentados da reabilitação cardíaca em pacientes com IAM com e sem insuficiência cardíaca (IC), há ainda, uma incerteza desse efeito no remodelamento ventricular. O remodelamento ventricular é um termo amplo empregado para descrever alterações genéticas, moleculares, neurohumorais, celulares e intersticiais que pode culminar com mudanças na geometria, massa, e função do ventrículo. No IAM, a depender da magnitude, esse evento pode determinar o surgimento de disfunção cardíaca aguda ou crônica tais como a insuficiência cardíaca. O termo remodelamento cardíaco reverso (RCR) tem sido empregado para definir a reversão parcial dessa condição como resposta a tratamento medicamentoso, uso de ressincronizadores implatáveis, ou mais incertamente o exercício físico aeróbio, trouxeram melhora na sobrevida e qualidade de vida (34).

O treinamento físico aeróbio tem resultados ainda conflitantes na literatura científica e não está claro como ele pode interferir no remodelamento ventricular e contribuir para um remodelamento ventricular reverso. Alguns estudos relataram atenuação do remodelamento ventricular com o treinamento físico (35,36), outros piora da progressão do remodelamento

(37,38) e outros, nenhuma relação com o remodelamento (39,40). Apesar dos resultados divergentes é importante atentar para o tempo de início dos programas de reabilitação cardíaca que não foram uniformes nos estudos e a gravidade clínica pós IAM assim como o nível de disfunção que pode ser verificada pela fração de ejeção do ventrículo esquerdo (FEVE).

Observando estes possíveis fatores de interferência nesses resultados, uma revisão sistemática com meta-análise verificou a influência de diferentes tempos de início da RC em pacientes pós IAM com FEVE reduzida e observou que melhores mudanças no remodelamento ventricular e capacidade cardiorrespiratória com o início precoce da RC, entre 6h a 7 dias (fase aguda) sem aumento dos eventos adversos (41). Apesar disso, os estudos inclusos na revisão anterior abordavam a RC continuada com um fase ambulatorial ampla, dessa forma, em nosso conhecimento, não há nenhum estudo avaliando a morfologia cardíaca imediatamente após a fase intra-hospitalar como forma de avaliar a eficácia desta fase da reabilitação.

5.0 Autonomia cardíaca após RC

A variabilidade da frequência cardíaca (VFC) é um método fácil e não invasivo para avaliar a integridade e funcionamento do sistema nervoso autônomo sobre o ritmo cardíaco, além de determinar riscos relacionados a doenças cardíacas e não cardíacas (42).

A VFC tem sido descrita extensivamente após IAM na avaliação do prognóstico após o evento. Um dos estudos iniciais descrevendo a relação da VFC após o IAM, verificou que a redução da VFC em paciente internados na unidade de tratamento crítico admitidos após IAM, aumentou a mortalidade intra-hospitalar (43). Confirmado posteriormente seu valor prognóstico de forma independente para previsão de mortalidade (44). Apesar da grande descrição científica sobre seu valor prognóstico poucos estudos tem se direcionado a busca dos efeitos terapêuticas sobre a VFC. Uma recente revisão sistemática investigou o efeito do exercício físico sobre a VFC pós IAM apenas em fase ambulatorial da RC mostrou que os resultados ainda são divergentes, onde de onze ensaios clínicos, cinco não demonstraram nenhum resultado sobre a VFC, porém

seis ensaios demonstraram resultados favoráveis em relação ao melhor controle autonômico após a RC baseada em exercícios (45).

Por fim, a literatura científica ainda apresenta grande lacuna de conhecimento em relação ao efeito da fase intra-hospitalar de RC sobre os parâmetros da VFC.

6.0 Dose da RC e aspectos de segurança

Mesmo com o reconhecimento dos efeitos e desfechos clínicos dos exercícios na reabilitação cardíaca serem dose-dependente, há importantes aspectos a serem considerados em termos de dose versus segurança no procedimento. O estudo de Kanazawa, 2020, verificou uma tendência de relação dose-resposta da RC intra-hospitalar e desfechos clínicos como risco de revascularização e readmissão hospitalar por todas as causas, obtendo-se uma redução significativa desses desfechos no grupo de pacientes que realizaram a partir de 8 sessões de RC durante a internação hospitalar (46). Apesar disso, nenhum ensaio clínico estabeleceu a dose numérica prescrita aos pacientes, combinando os parâmetros da prescrição e fornecendo um direcionamento específico da dose de exercícios realizada durante a fase intra-hospitalar da RC e, portanto, necessária para garantir de forma segura os benefícios a saúde. A ausência de padronização de dose x segurança cardíaca no procedimento, fragiliza a adoção de estratégias precoces e seguras para manutenção ou mesmo adoção dos procedimentos de RC após o evento cardíaco numa fase hospitalar precoce.

Outro estudo recente de Keteyian et al, 2018, analisando retrospectivamente em 5 anos uma grande amostra de pacientes que fizeram RC mostraram importantes achados. Pacientes que aumentaram 1 MET na carga de trabalho de treinamento ao final da RC apresentaram um menor risco ajustado de 38% e 42% de chance de internação e mortalidade por todas as causas. Este fato aponta para a necessidade do monitoramento a cerca das variáveis de prescrição de exercício durante a RC como forma de garantir o maior ganho da ACR possível para cada paciente considerando seu impacto na sobrevida (47). Essa mensagem também deve ser incorporada durante a prescrição inicial da RC intra-hospitalar.

Inúmeras evidências demonstram, portanto, o potencial efeito da ACR sobre os desfechos clínicos. O incremento da ACR e seu efeito protetor está diretamente relacionado a dose de exercício aeróbico prescrito durante a RC. A dose de exercício aeróbico pode ser definida como o total de energia despendida no exercício durante uma semana de treinamento e matematicamente descrita através da análise dos parâmetros de prescrição do treinamento, tais como a frequência, intensidade e o tempo. O posicionamento sobre progressão da dose de exercício na RC da AACVPR (American Association of Cardiovascular and Pulmonary Rehabilitation) junto ao ACSM (American College of Sports Medicine) em 2018, demonstram que o valor de dose de exercício aeróbico entre 500-1000 METxmin/Semana geram redução de mortalidade prematura e doenças cardiovasculares de forma significativa (48).

Diante dos achados com relação à temática dos resultados da RC sobre a capacidade funcional e seu impacto na morbimortalidade dos pacientes, observou-se a carência por estudos que focassem em dosar o exercício aeróbico prescrito durante a RC intra-hospitalar e determinar a eficácia e segurança desta dose. Além disso, sabendo que quanto maior o nível de aptidão cardiorrespiratória ao final da RC melhor a taxa de sobrevida é notadamente importante estudar o impacto da dosagem do exercício na busca da otimização dos resultados da RC, visto que há ainda pacientes que são pouco ou não respondedores em relação ao ganho de ACR após o programa.

Este estudo se torna necessário, portanto, considerando a importância da determinação da dose ideal de treinamento aeróbico durante a RC intra-hospitalar na busca da otimização dos resultados na ACR e de seu impacto no prognóstico clínico dos pacientes comparado ao processo de RC convencional. Servindo de modelo para o cálculo da dose de treino aeróbico de fácil inclusão da rotina clínica dos setores intra-hospitalares de RC. Sendo também importante para verificar a repercussão morfológica e funcional cardíaca da RC precoce, assim como o impacto clínico dessa mudança no aspecto físico e funcional do paciente. Por fim estabelecerá o nível de segurança cardiohemodinâmica desta terapêutica em fase precoce.

Dessa forma, o objetivo geral deste estudo será descrever a dose de exercício aeróbico ofertada durante a RC precoce e seu efeito sobre a aptidão física, morfologia e função cardíaca e aspectos de segurança em pacientes após IAM.

OBJETIVOS

Objetivo geral

Avaliar a eficácia e segurança da RC precoce após IAM e seu efeito sobre a aptidão física, morfologia e função cardíaca.

Objetivos específicos

- Verificar a variação da capacidade funcional por meio de medida da aptidão cardiorrespiratória, após RC intra-hospitalar utilizando como medida de resultado o VO_2 máximo (consumo máximo de oxigênio);
- Analisar a dose de treino aeróbico fornecida aos pacientes em RC intra-hospitalar e sua repercussão no desfecho de capacidade funcional;
- Relacionar o método padrão de medida do VO_2, o teste cardiopulmonar, a um método submáximo de avaliação, o teste de senta-levanta de 30s avaliando sua validade e reprodutibilidade;
- Observar a influência da RC precoce sobre os parâmetros morfológicos de remodelamento ventricular através da ressonância nuclear magnética (RNM);
- Investigar a variabilidade da frequência cardíaca após a reabilitação cardíaca precoce.
- Identificar a taxa de eventos adversos e o nível de segurança do protocolo avaliado neste ensaio clínico;

MATERIAL E MÉTODO

Delineamento do estudo

Trata-se de um protocolo de ensaio clínico, controlado, randomizado, simples-cego com dois grupos, razão de alocação de 1:1 e hipótese de superioridade que será realizado com pacientes admitidos na Unidade de Terapia Intensiva (UTI) adulto do Hospital Universitário Onofre Lopes (HUOL) com o diagnóstico clínico de Infarto agudo do miocárdio. A **Figura 1** representa o fluxograma de acompanhamento do estudo de acordo com o CONSORT (Consolidated Standards of Reporting Trials) (49). A **Figura 2** apresenta o fluxograma com as etapas principais do estudo.

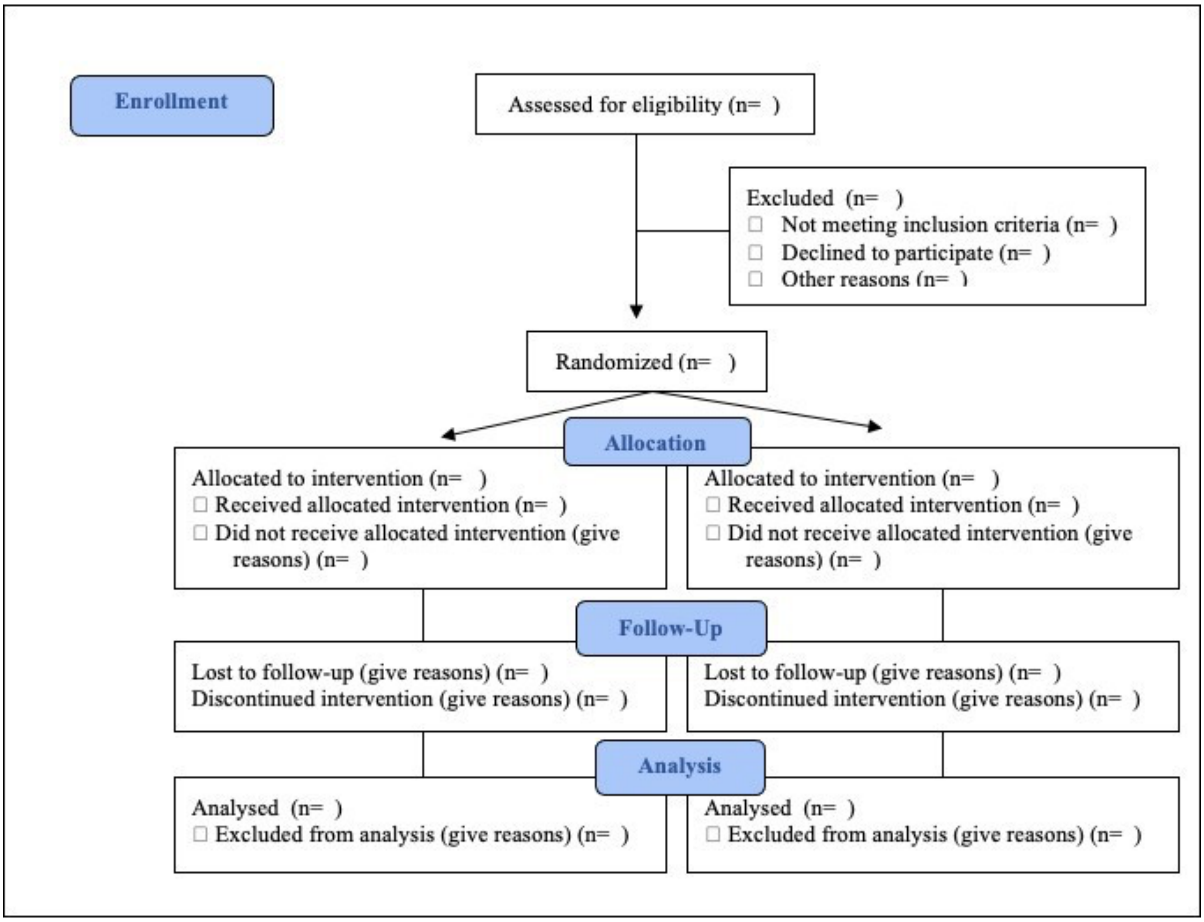

Fig. 1 – Fluxograma do estudo de acordo com o Consolidated Standards of Reporting Trials (CONSORT).

Delineamento do estudo

ELEGIBILIDADE
Pacientes com IAM não complicado (KILLIP I ou II/ >8h de estabilidade clínica) submetidos a ICP e admitidos na UTI adulto (HUOL)
Iniciam as orientações e mobilização precoce na UTI

↓

AVALIAÇÃO INICIAL (48h após admissão na UTI)
(1) TSL de 30s;
(2) RNM cardíaca;
(3) Variabilidade da FC;

↓

RANDOMIZAÇÃO (N=24)

↙ ↘

GRUPO CONTROLE (n=12)

Mobilização precoce + orientação de deambulação livre / Tratamento medicamentoso padrão

GRUPO INTERVENÇÃO (n=12)
Mobilização precoce + orientação de deambulação livre / Tratamento medicamentoso padrão

Protocolo de RC precoce com exercícios progressivos (Fase supervisionada e semi-sepervisionada)

↓ ↓

REAVALIAÇÃO - (Retorno ambulatorial - 30 dias)
(1) TSL de 30s;
(2) TECP;
(3) RNM cardíaca;
(4) Variabilidade da FC;

↓ ↓

SEGUIMENTO - (Retorno ambulatorial - 90 dias)

(5) RNM cardíaca;

Fig. 2 – Fluxograma com especificação das avaliações e intervenções realizadas.

Local da pesquisa

Este estudo será desenvolvido no Hospital Universitário Onofre Lopes (HUOL), na Unidade de Terapia Intensiva (UTI) adulto e nas enfermarias clínicas, localizado no município de Natal/Rio Grande do Norte.

Participantes da pesquisa

Os pacientes incluídos no estudo serão: Pacientes admitidos na UTI adulto do HUOL com diagnóstico clínico confirmado de IAM (ECG e enzimas cardíacas) com ou sem supra de ST e não complicados (Killip I ou II), submetidos a intervenção coronariana percutânea (ICP) com sucesso (Fluxo timi 2 ou 3), com faixa etária entre 18 anos e 70 anos e de ambos os sexos, classificados com baixo risco (FE>50%, ausências de disritmias ventriculares, ausência de depressão clínica e de sinais ou sintomas isquêmicos após o procedimento) segundo a classificação de risco do American Association of Cardiovascular and Pulmonary Reabilitation (AACPR) (50) e capacidade de fornecer consentimento livre e esclarecido. Os critérios de exclusão determinados incluem presença de alterações osteomioarticulares que impeçam a realização dos exercícios, sinais e sintomas de isquemia ou descompensação cardíaca, arritmias atriais ou ventriculares e doenças pulmonares diagnosticadas (ex: Hipertensão pulmonar, DPOC, entre outras).

Amostragem e recrutamento

Os participantes serão recrutados na unidade de terapia intensiva (UTI) adulto logo após admissão por procedimento de intervenção coronária percutânea (ICP).

O tamanho da amostra foi calculado através do software OpenEpi através da diferença média encontrada em estudo anterior (29) com RC intra-hospitalar para análise do consumo máximo de oxigênio (VO2). Um número de 7 participantes por grupo foi verificado com total de 14 para a amostra considerando um nível de significância de 5%, um poder estatístico de 80%. Acrescendo uma taxa de 30% para as possíveis perdas durante o estudo, optamos por recrutar 12 participantes por grupo para um tamanho total de amostra de 24 pacientes.

Randomização e cegamento

A sequência de randomização será gerada por um software disponível online através do site www.randomizer.org por um colaborador do estudo não envolvido nos processos de coleta ou avaliação dos resultados. O software vai gerar uma sequência de números uniformemente aleatórios para 2 grupos. A sequência gerada pelo programa será colocada em envelopes opacos e numerados sequencialmente.

Os envelopes opacos e selados ficarão sob domínio apenas do pesquisador responsável pela randomização. Apenas os colaboradores encarregados de administrar o protocolo de intervenção terão ciência do grupo de alocação de cada participante. Ademais todos os avaliadores de resultados permanecerão cegos durante o estudo para a alocação dos participantes.

Programa de reabilitação cardíaca precoce

O programa de reabilitação cardíaca (RC) precoce baseada em exercício físico será realizado em 3 etapas (Etapa 1 -Mobilização funcional; Etapa 2 – Reabilitação cardíaca precoce; Etapa 3 – Reabilitação cardíaca continuada).

Na etapa 1 de mobilização precoce, todos os pacientes do grupo controle e intervenção, a partir de 8 horas de estabilidade clínica (Ausência de sinais e sintomas isquêmicos) realizarão exercícios de incremento de mobilidade para garantir mobilidade funcional até alta da UTI, conforme etapa 1 do protocolo de RC intra-hospitalar em (**Apêndice B)**. Após garantir mobilidade funcional os pacientes serão randomizados para os grupos controle ou intervenção. Ambos os grupos realizarão a etapa 1 até o STEP 3 do protocolo de RC precoce, orientações sobre atividade física e deambulação de livre demanda além do tratamento medicamentoso padronizado, porém, apenas o grupo intervenção seguirá para o STEP 4 da etapa 1 e as etapas 2 e 3 da reabilitação cardíaca precoce e continuada.

Na etapa 2 os pacientes seguirão a reabilitação cardíaca precoce baseada em exercício realizada seguindo todos os parâmetros de prescrição (FITT-VP) e critérios de segurança recomendados pelo ACSM, 2018 (33). O protocolo institucional (**Apêndice B**) será realizado com **Frequência** de 2 sessões por dia, durante toda a internação com média de 8 a 10 sessões totais; **Intensidade** baixa para treino aeróbico com METs estimado pela percepção subjetiva de esforço de BORG entre 9 a 12 atingindo entre 2 a 3 METs e para o treino resistido com 2 a 3 séries de 60% das repetições máximas atingidas no teste de senta-levanta de 30s; **Tipo** de exercícios envolvidos serão o treino aeróbico utilizando o cicloergômetro associado a treino resistido usando o peso do próprio corpo com treino funcional de sentar-levantar; **Tempo** de treino aeróbico variando entre 3 a 20 minutos. O **Volume ou Dose** do treino aeróbico será mensurado com o cálculo da dose de treino por sessão e dose total aplicada durante a internação, fornecendo um valor em tempo x intensidade (METs) x frequência de sessões acumuladas e quantidade total de sessões x tempo. A **progressão** estruturada no protocolo tem enfoque no tempo de exercício aeróbico e na quantidade de séries do treino resistido. As sessões de exercício seguirão a orientação

do protocolo porém apresentam prescrições individualizadas com base tempo de tolerânciadurante o treino aeróbico com cicloergômetro e o treino resistido a partir da quantidade máxima de repetições atingidas no TSL de 30s avaliado individualmente antes da randomização.

Antes da alta hospitalar os pacientes do grupo intervenção receberão uma cartilha de orientação de exercícios (**Apêndice C**) para realizar a etapa 3 da reabilitação cardíaca continuada e semi- supervisionada com o tempo de exercício atingido ao final da reabilitação intra-hospitalar, frequência de 5 x por semana e progressão de 5 minutos a cada semana dentro do primeiro mês após a alta hospitalar, conforme tolerância e auto-monitoramento da intensidade através da percepção de esforço por meio da escala de Borg mantida entre 9 a 12, para garantir exercícios de leve a moderada intensidade. Nessa fase os pacientes do grupo intervenção serão acompanhados semanalmente através de contato telefônico e receberão um diário (**Apêndice C**) para anotação do tempo de atividade diária e semanal que deverá entregar ao investigar no retorno para reavaliação 30 dias após a alta hospitalar.

Todos os pacientes serão monitorados antes, durante e após cada sessão de atendimento supervisionado com frequencímetro e oxímetro de pulso (Modelo Nonin Onyx 9500, Nonin Medical, EUA e Polar H10), que permitirá a avaliação da frequência cardíaca e saturação de O_2. A pressão arterial será avaliada antes e de depois da sessão e durante a internação na UTI o eletrocardiograma será monitorado durante todo o exercício. Os pacientes serão orientados a interromper o exercício em caso de mal-estar (dispneia, sudorese excessiva, cianose, uso de musculatura acessória, vertigem, ânsia de vômito), dor no peito ou borg maior que 13 tanto para dispneia quanto para fadiga muscular.

Procedimentos de coleta de dados

Os participantes da pesquisa serão avaliados nos seguintes momentos: Primeira avaliação será realizada após 48 horas de internação na UTI. A segunda avaliação ocorrerá a partir do dia 30 após alta hospitalar durante a reavaliação ambulatorial e a terceira avaliação ocorrerá com 90

dias após a alta hospitalar para repetir a RNM. Os testes avaliativos realizados em cada uma das etapas são descritos na linha temporal descrita na **Figura 3**.

Anamnese e avalição clínico-física

Inicialmente será realizada uma entrevista com o voluntário, onde será preenchida a ficha de avaliação clínico-física (**Apêndice A**), contendo dados quanto à identificação dos participantes, antecedentes patológicos pessoais e familiares, queixas, hábitos de vida, medicações utilizadas, procedimento prévios, exames admissionais (Biomarcadores, ECG, ECO-TT e CATE); Além da avaliação física com coleta de dados dos sinais vitais e dados antropométricos.

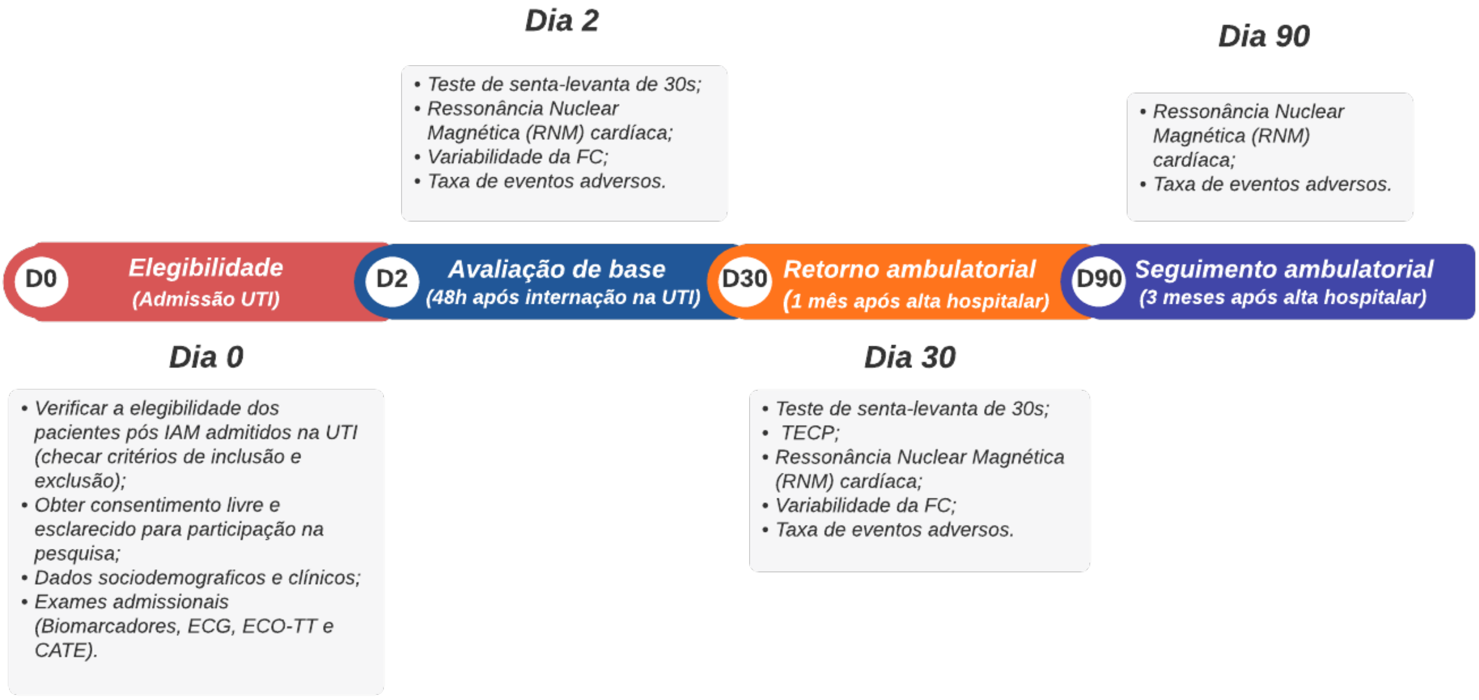

Fig. 3 – Linha temporal das avaliações.

Análise de Aptidão Física

Teste de Esforço Cardiopulmonar (TECP)

O teste incremental máximo do tipo Teste Esforço Cardiopulmonar (TECP) ou Teste Ergoespirométrico será utilizado para medir capacidade funcional através da ACR (Analisada pelo valor do VO2) após a RC.

Os pacientes realizarão o TECP em esteira rolante (modelo Centurion 300, Micromed, Brasil) conduzido sob o protocolo de carga incremental previamente elaborado por Weber[33] para que a exaustão ocorra entre 8 e 12 minutos através do sistema de teste ErgoPC Elite (Micromed, Brasil), e todos realizarão o mesmo protocolo (Rampa). Vertes do teste, os pacientes serão monitorados por eletrocardiograma de repouso em 12 derivações (ECG Digital, Micromed, Brasil), (SpO2) será monitorizada através do oxímetro Nonin 2500. Todos os pacientes terão instruções básicas relacionadas aos procedimentos e à utilização da escala de percepção subjetiva de esforço monitorada, utilizando-se como instrumento a escala de Borg (51). Todos os procedimentos serão realizados com supervisão médica e os pacientes serão autorizados a realizar o teste apoiando-se nas barras frontais de apoio da esteira ergométrica.

O analisador de gases metabólicos Cortex Metamax 3B (Alemanha), e o software Metalyzer 3B serão utilizados para captação, análise e interpretação dos gases expirados. O sistema consta de monitorização *breath-by-breath,* utilizando uma máscara de silicone com espaço morto de 45ml, onde é acoplada a turbina de volume para medir volume minuto (VE), e uma linha de gás conectada a uma célula de oxigênio e de gás carbônico para leitura de VO_2, e VCO_2, respectivamente. O sistema permite a transmissão de dados para o software, permitindo o monitoramento em tempo real das medidas de VO_2 relativo à massa corporal (VO_2/Kg), ventilação minuto (VE), razão de troca respiratóriverR), o equivalente ventilatório de gás carbônico (VE/VCO_2) e de oxigênio (VE/VO_2), e o pulso de oxigênio. O analisador de gases será calibrado diariamente.

A máscara de silicone com a turbina e linha de gás será acoplada no voluntário antes do início do teste, e respeitado um período de dois minutos permitindo a adaptação à máscara, evitando captação de medidas de hiperventilação. Então a participante se manterá em posição ortostática em repouso por dois minutos para medição do VO_2 basal, e respeitado VE, R e VO_2

adequados para início do teste. A VE de repouso ideal para início do exercício situa-se entre 8 e 15L/min, o R entre 0,75 e 0,85 e o $VO_{2REPOUSO}$ próximo a 3,5mL/kg/min, correspondente a 1 MET.

Os participantes serão instruídos a realizar o teste até o momento em que se sentirem incapazes de continuar. Durante todo o teste serão registradas as medidas cardiorrespiratórias e metabólicas. O eletrocardiograma em 12 derivações será monitorado continuamente e fornecerá os valores de frequência cardíaca. A pressão arterial será medida utilizando um esfigmomanômetro de coluna de mercúrio em intervalos de dois minutos, a saturação periférica de oxigênio (SpO2) será monitorizada continuamente através do oxímetro Nonin 2500, a percepção subjetiva de esforço será coletada em intervalos de dois minutos e na exaustão (2).

Os critérios para interrupção dos testes serão rigorosamente seguidos de acordo com a padronização da ATS 2003 (52). Os TECPs serão realizados na Unidade de Reabilitação Cardíaca do Hospital Universitário Onofre Lopes (CORE-HUOL) (**Figura 4**) que tem temperatura ambiente controlada e equipamentos e medicamentos necessários para prestar cuidados de urgência.

Após a finalização do teste, o paciente permanece monitorizado por cinco minutos para verificar o comportamento metabólico e cardiorrespiratório durante a recuperação, sendo três minutos de recuperação ativa e dois minutos de recuperação passiva. Após esse período, foram realizadas novas aferições de PA, SpO_2 e Borg.

Caso alguns dos testes necessite ser interrompido por desconforto do paciente (fadiga, dispneia, tontura), o mesmo receberá os cuidados necessários para a sua recuperação. Nos dias de realização dos testes de esforço máximo, os pacientes serão auxiliados por uma médica cardiologista, em um ambiente hospitalar e caso necessário será ofertado ao paciente o suporte adequado, como descanso de 5 a 10 minutos, monitorização dos sinais vitais e até encaminhamento para um setor do hospital com oxigenoterapia e maior suporte terapêutico, de acordo com a avaliação clínica realizada pela médica presente.

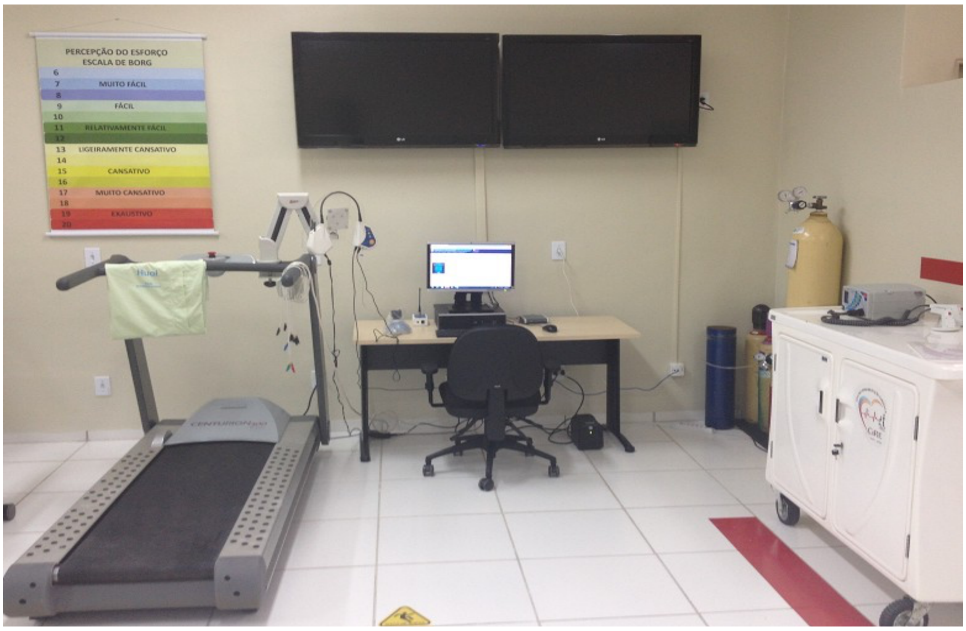

Fig. 4 - Setor de reabilitação cardíaca- Ala de avaliação funcional (CORE-HUOL).

Teste de senta-levanta de 30 segundos

O teste de senta-levanta de 30 segundos é um teste submáximo indicado e validado para indivíduos mais debilitados como idosos ou pacientes internados em ambiente hospitalar (30,31). Para a realização do teste utilizaremos conforme estudos anteriores e recomendação padronizada, uma cadeira rígida, com altura padrão de 45cm e sem apoio para os braços. O paciente será orientado a realizar o mais rápido possível num tempo de 30 segundos o movimento de sentar e levantar da cadeira que será contabilizado pelo avaliador. O TSL será realizado em 2 momentos: Na alta da UTI e com 30 dias após a alta hospitalar em ambos os grupos do estudo.

A avaliação da confiabilidade intra e inter-examinadores do TSL de 30s em pacientes pós IAM será realizada a partir da filmagem dos participantes realizando o TSL30s e avaliadores previamente selecionados para pontuarem os testes filmados. A amostra total de 24 participantes será avaliada. O teste será administrado pelo investigador principal que estará cego com relação a alocação dos participantes nos grupos do estudo. Dez avaliadores serão recrutados da equipe de fisioterapeutas do hospital das unidades de UTI e enfermarias. Todos os avaliadores serão

previamente treinados pelo investigador principal antes das avaliações. Após a realização e filmagem dos testes no momento da alta da UTI (Primeira avaliação), os testes filmados serão apresentados aos avaliadores que farão a pontuação de cada teste. Após 30 dias os mesmos testes filmados serão novamente apresentados aos mesmos avaliadores para reavaliação.

Análise de Morfologia e Função Cardíaca

Morfologia cardíaca

A ressonância nuclear magnética (RNM) cardíaca pode ser utilizada para diversas avaliações importantes após o infarto agudo do miocárdico. A RNM permite a realização do dignóstico para o infarto do miocárdio em sua condição aguda ou crônica, fornece vários indicadores prognósticos que podem impactar na estratificação de risco dos pacientes, além de permitir a identificação de complicações pós IAM, tais como, aneurisma, pericardites, trombos entre outros (53).

A avaliação da extensão cicatricial após o IAM é uma valiosa medida preditora do sucesso terapêutico, sendo utilizada como *endpoint* comum em ensaios clínicos. A extensão do infarto está inversamente relacionada ao prognóstico clínico e é um preditor mais sensível que a massa e a fração de ejeção ventricular da taquicardia ventricular, por fim, prevê mortalidade por todas as causas e sobrevida independente as fração de ejeção (53).

Neste estudo serão realizadas os protocolos padrão para avaliação da morfologia e função cardíaca, tais como, volumes sistólicos e diastólicos finais e fração de ejeção ventricular avaliadas através da sequência CINE SSFP além da sequência de realce tardio com gadolíneo (53). O exame será realizado no *baseline,* ainda durante a internação hospitalar, em torno do quarto ao sexto dia após o IAM e em dois momentos de *follow-up,* o priemiro a partir de 4 semanas após o evento, com tolerância de até 2 semanas para realização da imagem e o segundo a partir de 3 meses após o evento com tolerância de duas semanas.

A técnica utilizada para avaliação da extensão cicatricial será a de realce tardio com administração do quelato de gadolíneo. O paciente receberá o quelato de gadolíneo entre 10 a 20 minutos antes do exame. Na presença de tecido cicatricial há aumento do espaço extracelular em relação ao meio intracelular devido as rupturas de membranas dos cardiomiócitos havendo maior impregnação do gadolíneo nesta região gerando hiperealce na imagem (53). A avaliação do tamanho da cicatriz utilizará o seguinte método semi-quantitativo: Pontuação visual de 17 áreas padronizadas, numa escala de 5 pontos, sendo 0= sem hiperealce, 1= 1-25%, 2= 26 – 50%, 3=51 – 75% e 4= 76 – 100% de hiperealce. A pontuação total da cicatriz é dada somando as pontuações de cada segmento e dividindo por 17 (53).

Variabilidade da frequência cardíaca

A variabilidade da frequência cardíaca (VFC) é um método fácil e não invasivo para avaliar a integridade e funcionamento do sistema nervoso autônomo sobre o ritmo cardíaco. A VFC pode ser observada pelos intervalos R-R e sua redução está normalmente relacionada ao envelhecimento e piores condições de saúde (42,44,45).

Nesta pesquisa a VFC será mensurada antes da randomização dos grupos e no retorno ambulatorial com 30 dias após alta hospitalar através do cardiofrequencímetro tipo cinta torácica de marca Polar, modelo H10. Os dados fornecidos pelo cardiofrequencímetro serão analisados através de software Kubios HRV.

As variáveis de interesse incluem as do domínio do tempo: SDNN - desvio padrão de todos os intervalos RR normais registrados em um intervalo de tempo, expresso em ms; pNN50 - representa a porcentagem de intervalos RR adjacentes com diferença de duração superior a 50 ms. Além dessas variáveis serão analisadas as do domínio da frequência: Componente de alta frequência (HF) com variação de 0,15 a 0,4 Hz; Componente de baixa frequência (LF), variando entre 0,04 e 0,15 Hz e a relação entre LF/HF (45).

Análise da dose de exercício aeróbico na RC

Apesar da meta padronizada para dose de exercício aeróbico, cada paciente apresentará um limite de tolerância individual para a progressão da dose de exercício, por causa disso cada paciente terá sua dose de exercício monitorizada a cada semana de RC.

O método de cálculo para dose de exercício aeróbico será realizado através da multiplicação entre o valor da intensidade estimada do treino em (MET-Taxa metabólica da tarefa) estimado pelo borg, o tempo da sessão e a frequência das sessões durante cada semana, determinando a dose em METxmin-Semana (48).

Segurança Cardiohemodinâmica da RC precoce

A segurança do protocolo institucional testado neste ensaio ocorrerá por meio de notificações dos eventos adversos durante e após as sessões de RC fornecidas aos pacientes do grupo intervenção. Faremos a notificação dos eventos adversos sessão por sessão elencando como principais: As arritmias, precordialgia, borg>13, queda da pressão arterial sistólica (PAS) acima de 10 mmHg e parada cardiorrespiratória. Outros eventos não elencados anteriormente também serão registrados dentro da ficha de acompanhamento e avaliação dos pacientes conforme **Apêndice A**.

Estratégia Estatística

As variáveis de interesse serão analisadas utilizando o software (Graph Pad Prism versão 7.0) e avaliadas inicialmente quanto à normalidade de distribuição dos dados pelo teste de Shapiro Wilk considerando n<30. Se tratando de dados com distribuição normal serão analisados quanto à média e desvio padrão. Os dados sem distribuição normal serão analisados quanto à mediana e percentis. As análises de comparação das variáveis funcionais físicas do

TSL, variáveis morfológicas da RNM e variáveis funcionais cardíacas da VFC dos tempos PRÉ-RC e PÓS-RC de cada grupo será realizada utilizando o teste de Wilcoxon se os dados foram não paramétricos e o teste T pareado se os dados foram paramétricos. A comparação entre os grupos controle e intervenção para o TECP será realizada por meio de teste T independente ou ManWhitney a depender da normalidade dos dados. A confiabilidade e validade da TSL será analisada a partir da correlação de Spearman e coeficiente de confiabilidade intraexaminador. Para todas as testagens de hipóteses será atribuída uma significância de 5% de probabilidade ($P<0,05$) de erro.

Aspectos Éticos

Este estudo será submetido ao Comitê de Ética e Pesquisa do Hospital Universitário Onofre Lopes, conforme determinações da Resolução nº 466/12 do Conselho Nacional de Saúde. Além disso, estará garantido a todos os participantes o fornecimento do termo de consentimento livre e esclarecido (**Apêndice D**), o termo de autorização institucional para uso de documentos dos pacientes (**Apêndice E**) e assegurado, durante todo o estudo, a manutenção do sigilo e o anonimato dos participantes.

RISCOS E BENEFÍCIOS

Os riscos que os pacientes envolvidos na pesquisa podem apresentar são aqueles inerentes à atividade física como dispneia, tontura, fadiga, exaustão, hipotensão, distensões musculares ou dores articulares. Contudo os riscos serão minimizados através das seguintes providências: o exercício proposto ocorrerá dentro dos critérios de segurança previstos pelo Colégio Americano de Medicina do Esporte; Para os casos de intercorrências mais graves como arritmias atriais e ventriculares, descompensação cardíaca ou mesmo parada cardiorrespiratória, o setor dispõe de material para ressuscitação cardiorrespiratória, bem como treinamento de todos profissionais da equipe dos setores de UTI e enfermaria cardiológica para manejo de possíveis intercorrências.

Com relação a fase semi-supervisionada os pacientes realizarão apenas intensidade baixa de exercícios com os quais já estarão adaptados durante a internação hospitalar, além do grande filtro de avaliação ao qual serão submetidos para serem elegidos através da estratificação de risco do American Association of Cardiovascular and Pulmonary Reabilitation (AACPR) (50). Por fim, apenas os pacientes de baixo risco serão elegíveis e farão acompanhamento semi-supervisionado após alta hospitalar conforme recomendado pela última Diretriz Brasileira de Reabilitação Cardiovascular de 2020 (54).

Dentre os benefícios desta pesquisa para os participantes, teremos a disponibilização de informações sobre a doença, avaliação e aconselhamento profissional sobre a capacidade funcional e aptidão cardiorrespiratória, com o fornecimento de uma cartilha de prescrição de exercício funcional individualizado após o TECP ao final do estudo (**Apêndice F**) com as instruções quanto à realização e dose do exercício, o que levará a um estímulo maior ao auto- cuidado eficiente, possibilitando melhor controle de sua doença, reduzindo risco de internações hospitalar e morbimortalidade.

RESULTADOS E IMPACTOS ESPERADOS

Espera-se observar por meio desta pesquisa um novo modelo de prescrição de exercício aeróbico em reabilitação cardíaca precoce baseado na meta de dose de exercício. Além disso, espera-se verificar a magnitude do impacto clínico da realização deste modelo de prescrição através da análise da aptidão cardiorrespiratória, medidas de capacidade funcional submáxima através do teste de senta-levanta e sobre os parâmetros morfológicos e funcionais do coração.

Esta pesquisa impacta no processo de reabilitação cardíaca intra-hospitalar e de seguimento precoce, visto que, traça uma nova estratégia terapêutica que poderá tornar o processo de RC mais eficiente na alteração da capacidade funcional dos pacientes e dessa maneira no prognóstico clínico desses pacientes. Por fim, fornece um modelo para cálculo da intensidade e dose do exercício aeróbico favorecendo a sua aplicabilidade prática nas unidades de internação hospitalar.

Este trabalho poderá ainda estimular novas linhas de pesquisas, visto que a análise da dose de exercício aeróbico ideal dentro da RC ainda precisa ser extensivamente estudada para que se obtenha um consenso firme a cerca da janela terapêutica a ser utilizada com os pacientes cardiopatas. Também se espera com este projeto apresentar todos os resultados em nível nacional e internacional, assim como publicar artigos em revistas de bom fator de impacto (>1), Qualis A1-A2. Por último, compreende-se que o estudo contribuirá para uma maior importância da aplicação deste tipo de reabilitação dentro da Fisioterapia Cardiovascular.

CRONOGRAMA

Atividade	Meses (2022)											
	Jan	Fev	Mar	Abr	Mai	Jun	Jul	Ago	Set	Out	Nov	Dez
Revisão de literatura	X	X	X	X	X	X	X	X	X	X	X	X
Submissão ao CEP-HUOL					X							
PILOTO							X					
Coleta de dados							X	X	X	X	X	X
Qualificação do projeto												X

Atividade	Meses (2023)											
	Jan	Fev	Mar	Abr	Mai	Jun	Jul	Ago	Set	Out	Nov	Dez
Revisão de literatura	X	X	X	X	X	X	X	X	X	X	X	X
Discussão dos resultados encontrados	X	X	X	X								
Submissão do artigo					X	X	X					
Defesa do doutorado								X				

ORÇAMENTO

**Custeio**<br>**Material permanente**			
Item	Preço Unitário	Quantidade	Total **(R$)**
Esteira ergométrica (Centurion 300)	995,00	1	995,00
Estetoscópio (Littmam) + Esfignomanometro	500,00	1	500,00
Oxímetro de pulso (Nonin Onyx- 9500)	300,00	1	300,00
Frequêncímetro tipo cinta torácica (H10 – POLAR)	500,00	2	1.000,00
Notebo"k 14" DELL inspiron 14-2630 preto core I3	2.400,00	1	2.400,00
**Subtotal**			**5.195,00**

**Custeio**<br>**Material de consumo**			
Item	Preço Unitário	Quantidade	Total **(R$)**
Toner para impressora HP	45,00	1	90,00
Resma de papel A4	15,00	3	45,00
Caneta esferográfica	10,00 1cx	1cx	10,00
Pasta plástica	5,00	3	15,00
Eletrodos para ECG (Meditrace)	40,00 cx	10	400,00
Envelopes de papel	73,00 cx	1	73,00
**Subtotal**			**633,00**

Os recursos gastos com materiais de consumo e permanentes totalizam um valor de **R$ 5.828,00**. Parte destes gastos, referentes aos materiais permanentes são patrimônios institucionais e se encontram a disposição para a pesquisa. O restante do valor será inteiramente de responsabilidade do pesquisador. Além dos materiais permanentes e de consumo o estudo necessitará do uso do telefone do setor (CORE-HUOL) para a realização das ligações para os pacientes na fase de reabilitação semi-supervisionada e convocação para reavaliação.

REFERÊNCIAS

1. 2020 ESC Guidelines for the management of acute coronary syndromes in patients presenting without persistent ST-segment elevation | European Heart Journal | Oxford Academic [Internet]. [citado 29 de abril de 2021]. Disponível em: https://academic.oup.com/eurheartj/article/42/14/1289/5898842
2. Meneghelo RS, Araújo CGS, Stein R, Mastrocolla LE, Albuquerque PF, Serra SM. III Diretrizes da Sociedade Brasileira de Cardiologia sobre teste ergométrico. Arq Bras Cardiol. 2010;95(5):1–26.
3. Ibanez B, James S, Agewall S, Antunes MJ, Bucciarelli-Ducci C, Bueno H, et al. 2017 ESC Guidelines for the management of acute myocardial infarction in patients presenting with ST-segment elevation. Eur Heart J. 7 de janeiro de 2018;39(2):119–77.
4. Townsend N, Wilson L, Bhatnagar P, Wickramasinghe K, Rayner M, Nichols M. Cardiovascular disease in Europe: epidemiological update 2016. Eur Heart J. 7 de novembro de 2016;37(42):3232–45.
5. Virani Salim S., Alonso Alvaro, Aparicio Hugo J., Benjamin Emelia J., Bittencourt Marcio S., Callaway Clifton W., et al. Heart Disease and Stroke Statistics—2021 Update. Circulation. 23 de fevereiro de 2021;143(8):e254–743.
6. Anderson L, Taylor RS. Cardiac rehabilitation for people with heart disease: an overview of Cochrane systematic reviews. Cochrane Database Syst Rev. 12 de dezembro de 2014;(12):CD011273.
7. L A, Dr T, N O, Ad Z, K R, N M, et al. Exercise-based cardiac rehabilitation for coronary heart disease [Internet]. Vol. 2016, The Cochrane database of systematic reviews. Cochrane Database Syst Rev; 2016 [citado 14 de outubro de 2020]. Disponível em: https://pubmed.ncbi.nlm.nih.gov/26730878/
8. Amsterdam EA, Wenger NK, Brindis RG, Casey DE, Ganiats TG, Holmes DR, et al. 2014 AHA/ACC guideline for the management of patients with non-ST-elevation acute coronary syndromes: executive summary: a report of the American College of Cardiology/American Heart Association Task Force on Practice Guidelines. Circulation. 23 de dezembro de 2014;130(25):2354–94.
9. Anderson L, Oldridge N, Thompson DR, Zwisler A-D, Rees K, Martin N, et al. Exercise-Based Cardiac Rehabilitation for Coronary Heart Disease: Cochrane Systematic Review and Meta-Analysis. J Am Coll Cardiol. 5 de janeiro de 2016;67(1):1–12.
10. De Schutter A, Kachur S, Lavie CJ, Menezes A, Shum KK, Bangalore S, et al. Cardiac rehabilitation fitness changes and subsequent survival. Eur Heart J Qual Care Clin Outcomes. 1º de julho de 2018;4(3):173–9.
11. Borghi-Silva A, Mendes RG, Trimer R, Cipriano G. Current trends in reducing cardiovascular disease risk factors from around the world: focus on cardiac rehabilitation in Brazil. Prog Cardiovasc Dis. abril de 2014;56(5):536–42.
12. Rauch B, Davos CH, Doherty P, Saure D, Metzendorf M-I, Salzwedel A, et al. The prognostic effect of cardiac rehabilitation in the era of acute revascularisation and statin therapy: A systematic review and meta-analysis of randomized and non-randomized studies - The Cardiac Rehabilitation Outcome Study (CROS). Eur J Prev Cardiol. 2016;23(18):1914–39.
13. van Halewijn G, Deckers J, Tay HY, van Domburg R, Kotseva K, Wood D. Lessons

from contemporary trials of cardiovascular prevention and rehabilitation: A systematic review and meta-analysis. Int J Cardiol. 1º de abril de 2017;232:294–303.
14. Sandesara PB, Lambert CT, Gordon NF, Fletcher GF, Franklin BA, Wenger NK, et al. Cardiac rehabilitation and risk reduction: time to “rebrand and reinvigorate”. J Am Coll Cardiol. 3 de fevereiro de 2015;65(4):389–95.
15. Lavie CJ, Arena R, Franklin BA. Cardiac Rehabilitation and Healthy Life-Style Interventions: Rectifying Program Deficiencies to Improve Patient Outcomes. J Am Coll Cardiol. 5 de janeiro de 2016;67(1):13–5.
16. Menezes AR, Lavie CJ, Milani RV, Forman DE, King M, Williams MA. Cardiac rehabilitation in the United States. Prog Cardiovasc Dis. abril de 2014;56(5):522–9.
17. Menezes AR, Lavie CJ, Forman DE, Arena R, Milani RV, Franklin BA. Cardiac rehabilitation in the elderly. Prog Cardiovasc Dis. outubro de 2014;57(2):152–9.
18. Grace SL, Bennett S, Ardern CI, Clark AM. Cardiac rehabilitation series: Canada. Prog Cardiovasc Dis. abril de 2014;56(5):530–5.
19. Humphrey R, Guazzi M, Niebauer J. Cardiac rehabilitation in Europe. Prog Cardiovasc Dis. abril de 2014;56(5):551–6.
20. Madan K, Babu AS, Contractor A, Sawhney JPS, Prabhakaran D, Gupta R. Cardiac rehabilitation in India. Prog Cardiovasc Dis. abril de 2014;56(5):543–50.
21. O’Connor GT, Buring JE, Yusuf S, Goldhaber SZ, Olmstead EM, Paffenbarger RS, et al. An overview of randomized trials of rehabilitation with exercise after myocardial infarction. Circulation. agosto de 1989;80(2):234–44.
22. Munir H, Fromowitz J, Goldfarb M. Early mobilization post-myocardial infarction: A scoping review. PLoS ONE [Internet]. 17 de agosto de 2020 [citado 30 de abril de 2021];15(8). Disponível em: https://www.ncbi.nlm.nih.gov/pmc/articles/PMC7430744/
23. Lavie CJ, Thomas RJ, Squires RW, Allison TG, Milani RV. Exercise training and cardiac rehabilitation in primary and secondary prevention of coronary heart disease. Mayo Clin Proc. abril de 2009;84(4):373–83.
24. Leon AS, Franklin BA, Costa F, Balady GJ, Berra KA, Stewart KJ, et al. Cardiac rehabilitation and secondary prevention of coronary heart disease: an American Heart Association scientific statement from the Council on Clinical Cardiology (Subcommittee on Exercise, Cardiac Rehabilitation, and Prevention) and the Council on Nutrition, Physical Activity, and Metabolism (Subcommittee on Physical Activity), in collaboration with the American association of Cardiovascular and Pulmonary Rehabilitation. Circulation. 25 de janeiro de 2005;111(3):369–76.
25. Martin B-J, Arena R, Haykowsky M, Hauer T, Austford LD, Knudtson M, et al. Cardiovascular fitness and mortality after contemporary cardiac rehabilitation. Mayo Clin Proc. maio de 2013;88(5):455–63.
26. Nogueira IDB, Servantes DM, Nogueira PA de MS, Pelcerman A, Salvetti XM, Salles F, et al. Correlação entre qualidade de vida e capacidade funcional na insuficiência cardíaca. Arq Bras Cardiol. agosto de 2010;95(2):238–43.
27. Smith PJ, Sherwood A, Mabe S, Watkins L, Hinderliter A, Blumenthal JA. Physical activity and psychosocial function following cardiac rehabilitation: One-year follow-up of the ENHANCED study. Gen Hosp Psychiatry. 2017;49:32–6.
28. Peixoto TCA, Begot I, Bolzan DW, Machado L, Reis MS, Papa V, et al. Early exercise-based rehabilitation improves health-related quality of life and functional capacity after acute myocardial infarction: a randomized controlled trial. Can J Cardiol. março de 2015;31(3):308–13.
29. Zanini M, Nery RM, de Lima JB, Buhler RP, da Silveira AD, Stein R. Effects of Different Rehabilitation Protocols in Inpatient Cardiac Rehabilitation After Coronary Artery

Bypass Graft Surgery: A RANDOMIZED CLINICAL TRIAL. J Cardiopulm Rehabil Prev. novembro de 2019;39(6):E19–25.
30. McAllister LS, Palombaro KM. Modified 30-Second Sit-to-Stand Test: Reliability and Validity in Older Adults Unable to Complete Traditional Sit-to-Stand Testing. J Geriatr Phys Ther 2001. setembro de 2020;43(3):153–8.
31. Zanini A, Crisafulli E, D'Andria M, Gregorini C, Cherubino F, Zampogna E, et al. Minimum Clinically Important Difference in 30-s Sit-to-Stand Test After Pulmonary Rehabilitation in Subjects With COPD. Respir Care. 1º de outubro de 2019;64(10):1261–9.
32. Arena Ross, Sietsema Kathy E. Cardiopulmonary Exercise Testing in the Clinical Evaluation of Patients With Heart and Lung Disease. Circulation. 15 de fevereiro de 2011;123(6):668–80.
33. American College of Sports Medicine. ACSM's Guidelines for Exercise Testing and Prescription. Vol. 10º. 2018.
34. Bellenger NG, Rajappan K, Rahman SL, Lahiri A, Raval U, Webster J, et al. Effects of carvedilol on left ventricular remodelling in chronic stable heart failure: a cardiovascular magnetic resonance study. Heart Br Card Soc. julho de 2004;90(7):760–4.
35. Giallauria F, Cirillo P, Lucci R, Pacileo M, De Lorenzo A, D'Agostino M, et al. Left ventricular remodelling in patients with moderate systolic dysfunction after myocardial infarction: favourable effects of exercise training and predictive role of N-terminal pro-brain natriuretic peptide. Eur J Cardiovasc Prev Rehabil Off J Eur Soc Cardiol Work Groups Epidemiol Prev Card Rehabil Exerc Physiol. fevereiro de 2008;15(1):113–8.
36. Wisløff U, Støylen A, Loennechen JP, Bruvold M, Rognmo Ø, Haram PM, et al. Superior cardiovascular effect of aerobic interval training versus moderate continuous training in heart failure patients: a randomized study. Circulation. 19 de junho de 2007;115(24):3086–94.
37. Jugdutt BI, Michorowski BL, Kappagoda CT. Exercise training after anterior Q wave myocardial infarction: importance of regional left ventricular function and topography. J Am Coll Cardiol. agosto de 1988;12(2):362–72.
38. Kubo N, Ohmura N, Nakada I, Yasu T, Katsuki T, Fujii M, et al. Exercise at ventilatory threshold aggravates left ventricular remodeling in patients with extensive anterior acute myocardial infarction. Am Heart J. janeiro de 2004;147(1):113–20.
39. Adachi H, Koike A, Obayashi T, Umezawa S, Aonuma K, Inada M, et al. Does appropriate endurance exercise training improve cardiac function in patients with prior myocardial infarction? Eur Heart J. outubro de 1996;17(10):1511–21.
40. Otsuka Y, Takaki H, Okano Y, Satoh T, Aihara N, Matsumoto T, et al. Exercise training without ventricular remodeling in patients with moderate to severe left ventricular dysfunction early after acute myocardial infarction. Int J Cardiol. fevereiro de 2003;87(2–3):237–44.
41. Zhang Y-M, Lu Y, Tang Y, Yang D, Wu H-F, Bian Z-P, et al. The effects of different initiation time of exercise training on left ventricular remodeling and cardiopulmonary rehabilitation in patients with left ventricular dysfunction after myocardial infarction. Disabil Rehabil. 2016;38(3):268–76.
42. Brateanu A. Heart rate variability after myocardial infarction: what we know and what we still need to find out. Curr Med Res Opin. 2015;31(10):1855–60.
43. Wolf MM, Varigos GA, Hunt D, Sloman JG. Sinus arrhythmia in acute myocardial infarction. Med J Aust. 15 de julho de 1978;2(2):52–3.
44. Heart rate variability: standards of measurement, physiological interpretation and clinical use. Task Force of the European Society of Cardiology and the North American Society of Pacing and Electrophysiology. Circulation. 1º de março de 1996;93(5):1043–65.

45. Figueiredo T de G, de Souza HCM, Neves VR, do Rêgo Barros AEV, Dornelas de Andrade A de F, Brandão DC. Effects of physical exercise on the autonomic nervous system in patients with coronary artery disease: a systematic review. Expert Rev Cardiovasc Ther. novembro de 2020;18(11):749–59.
46. Kanazawa N, Iijima H, Fushimi K. In-hospital cardiac rehabilitation and clinical outcomes in patients with acute myocardial infarction after percutaneous coronary intervention: a retrospective cohort study. BMJ Open. 1º de setembro de 2020;10(9):e039096.
47. Keteyian SJ, Kerrigan DJ, Lewis B, Ehrman JK, Brawner CA. Exercise training workloads in cardiac rehabilitation are associated with clinical outcomes in patients with heart failure. Am Heart J. 10 de julho de 2018;204:76–82.
48. Squires RW, Kaminsky LA, Porcari JP, Ruff JE, Savage PD, Williams MA. Progression of Exercise Training in Early Outpatient Cardiac Rehabilitation: AN OFFICIAL STATEMENT FROM THE AMERICAN ASSOCIATION OF CARDIOVASCULAR AND PULMONARY REHABILITATION. J Cardiopulm Rehabil Prev. 2018;38(3):139–46.
49. Schulz KF, Altman DG, Moher D, the CONSORT Group. CONSORT 2010 Statement: updated guidelines for reporting parallel group randomised trials. BMC Med. 24 de março de 2010;8(1):18.
50. Williams MA. Exercise testing in cardiac rehabilitation. Exercise prescription and beyond. Cardiol Clin. agosto de 2001;19(3):415–31.
51. Borg GA. Psychophysical bases of perceived exertion. Med Sci Sports Exerc. 1982;14(5):377–81.
52. American Thoracic Society, American College of Chest Physicians. ATS/ACCP Statement on cardiopulmonary exercise testing. Am J Respir Crit Care Med. 15 de janeiro de 2003;167(2):211–77.
53. Rajiah P, Desai MY, Kwon D, Flamm SD. MR imaging of myocardial infarction. Radiogr Rev Publ Radiol Soc N Am Inc. outubro de 2013;33(5):1383–412.
54. Carvalho T de, Milani M, Ferraz AS, Silveira AD da, Herdy AH, Hossri CAC, et al. Diretriz Brasileira de Reabilitação Cardiovascular – 2020. Arq Bras Cardiol. 22 de maio de 2020;114(5):943–87.

APÊNDICES

APÊNDICE A – FICHA DE AVALIAÇÃO CLÍNICA

UNIVERSIDADE FEDERAL DO RIO GRANDE DO NORTE – UFRN
HOSPITAL UNIVERSITÁRIO ONOFRE LOPES – HUOL
Avaliação Fisioterapêutica

Data Avaliação:_____/____/_____ Reavaliação:_____/____/_____

**IDENTIFICAÇÃO E DADOS SOCIODEMOGRÁFICOS**
**Nome:**________________________________**Número do Prontuário:** _______________________________________<br>**Data de nascimento:**___/___/____ **Idade:**________anos **Telefone:**______________________________________<br>**Endereço:**____<br>**Sexo:** □ M □ F **Peso:**___**Altura**:_____**Estado Civil:** □ Casado □Solteiro □Divorciado □Viúvo **Escolaridade:**<br>□ Analfabeto □ 1°grau □ 2°grau □ 3°grau □ Completo □ Incompleto<br>**Profissão:**____

**DADOS CLÍNICOS**
**Fatotes de risco cardiovascular:** □ HAS □ DM □ Dislipidemia □ Sedentário □ Fumante □ Histórico familiar<br>□ Sobrepeso<br><br>**Medicação cardiovascular POS IAM:** □ Beta-bloqueador<br>□ Inibidores da ECA<br>□ Diuréticos<br>□ Estatinas<br>□ Antiplaquetários<br>□ Hipoglicemiantes<br>□Outras____<br><br>**IAMCSST:** □ Sim □ Não Localização: □ Anterior □ Inferior □ Lateral<br><br>**Angioplastia:** □ Stent Artéria culpada:___________________________Killip:________Timi:____________________<br>Tempo para reperfusão:_______________________CKMB (pico):_____Troponina (pico):________

EXAME FÍSICO POR SESSÃO

Sessão 1: Início da sessão: FC:_______bpm FR:_______irpm SpO_2:______% PA:_______mmHg
Final da sessão: FC:_______bpm FR:_______irpm SpO_2:______% PA:_______mmHg
Segurança: □ Precordialgia □ Arritmia__________ □ Borg>=13 □ Queda da PAS>=10mmHg
□ PCR □ Outros:______________DVA (Dose) □________Dispositivos:________________
Resumo da RC:____

Sessão 2: Início da sessão: FC:_______bpm FR:_______irpm SpO_2:______% PA:_______mmHg
Final da sessão: FC:_______bpm FR:_______irpm SpO_2:______% PA:_______mmHg
Segurança: □ Precordialgia □ Arritmia__________ □ Borg>=13 □ Queda da PAS>=10mmHg
□ PCR □ Outros:______________DVA (Dose) □________Dispositivos:________________
Resumo da RC:____

Sessão 3: Início da sessão: FC:_______bpm FR:_______irpm SpO_2:______% PA:_______mmHg
Final da sessão: FC:_______bpm FR:_______irpm SpO_2:______% PA:_______mmHg
Segurança: □ Precordialgia □ Arritmia__________ □ Borg>=13 □ Queda da PAS>=10mmHg
□ PCR □ Outros:______________DVA (Dose) □________Dispositivos:________________
Resumo da RC:____

Sessão 4: Início da sessão: FC:_______bpm FR:_______irpm SpO_2:______% PA:_______mmHg
Final da sessão: FC:_______bpm FR:_______irpm SpO_2:______% PA:_______mmHg
Segurança: □ Precordialgia □ Arritmia__________ □ Borg>=13 □ Queda da PAS>=10mmHg
□ PCR □ Outros:______________DVA (Dose) □________Dispositivos:________________
Resumo da RC:____

Sessão 5: Início da sessão: FC:_______bpm FR:_______irpm SpO_2:______% PA:_______mmHg
Final da sessão: FC:_______bpm FR:_______irpm SpO_2:______% PA:_______mmHg
Segurança: □ Precordialgia □ Arritmia__________ □ Borg>=13 □ Queda da PAS>=10mmHg
□ PCR □ Outros:______________DVA (Dose) □________Dispositivos:________________
Resumo da RC:____

Sessão 6: Início da sessão: FC:_______bpm FR:_______irpm SpO_2:______% PA:_______mmHg
Final da sessão: FC:_______bpm FR:_______irpm SpO_2:______% PA:_______mmHg
Segurança: □ Precordialgia □ Arritmia__________ □ Borg>=13 □ Queda da PAS>=10mmHg
□ PCR □ Outros:______________DVA (Dose) □________Dispositivos:________________
Resumo da RC:____

Sessão 7: Início da sessão: FC:_______bpm FR:_______irpm SpO_2:______% PA:_______mmHg
Final da sessão: FC:_______bpm FR:_______irpm SpO_2:______% PA:_______mmHg
Segurança: □ Precordialgia □ Arritmia__________ □ Borg>=13 □ Queda da PAS>=10mmHg
□ PCR □ Outros:______________DVA (Dose) □________Dispositivos:________________
Resumo da RC:____

Sessão 8: Início da sessão: FC:_______bpm FR:_______irpm SpO_2:______% PA:_______mmHg
Final da sessão: FC:_______bpm FR:_______irpm SpO_2:______% PA:_______mmHg
Segurança: □ Precordialgia □ Arritmia__________ □ Borg>=13 □ Queda da PAS>=10mmHg
□ PCR □ Outros:______________DVA (Dose) □________Dispositivos:________________
Resumo da RC:____

Sessões excedentes: Algum evento adverso? Qual?____

EXAMES COMPLEMENTARES	
**Eletrocardiograma** (1): ____<br>□ Ritmo regular □Fibrilação atrial □Marcapasso □BRD □BRE □Outros _____________	Data:___/ /___
**Ecocardiograma** (1): FE_______PAP_________Outros:______________________	Data:___/ /___
**Raio X** (1):____	Data:___/ /___
**Cateterismo cardíaco** (1):____<br>____	Data:___/ /___
**Ressonância cardíaca** (1):____	Data:___/ /___
**Ressonância cardíaca** (2):____	Data:___/ /___
**Variabilidade da FC cardíaca** (1):______________________________________	Data:___/ /___
**Variabilidade da FC cardíaca** (2):______________________________________	Data:___/ /___

TESTES FÍSICOS	
**TSL 30s** (1): Resultado:____Evento adverso:___________________________<br>Antes do teste: FC:_____bpm FR:_____irpm SpO_2:____% PA:_______mmHg<br>Final do teste: FC:____bpm FR:_____irpm SpO_2:____% PA:_______mmHg	Data:___/___/___
**TSL 30s** (1 + 30min): Resultado:____Evento adverso:____________________<br>Antes do teste: FC:_____bpm FR:_____irpm SpO_2:____% PA:_______mmHg<br>Final do teste: FC:____bpm FR:_____irpm SpO_2:____% PA:_______mmHg	
**TSL 30s** (2): Resultado:____Evento adverso:___________________________<br>Antes do teste: FC:_____bpm FR:_____irpm SpO_2:____% PA:_______mmHg<br>Final do teste: FC:____bpm FR:_____irpm SpO_2:____% PA:_______mmHg	Data:___/___/___
**TSL 30s** (2 + 30min): Resultado:____Evento adverso:____________________<br>Antes do teste: FC:_____bpm FR:_____irpm SpO_2:____% PA:_______mmHg<br>Final do teste: FC:____bpm FR:_____irpm SpO_2:____% PA:_______mmHg	
**TECP (2)**: $VO_{2\ PICO}$:_____Tempo de teste: _____________________________<br>Variáveis derivadas:____	Data:___/___/___

APÊNDICE B – PROTOCOLO DE REABILITAÇÃO CARDÍACA BASEADA EM EXERCÍCIO INTRA-HOSPITALAR.

Protocolo de RC precoce – ETAPA 1

**STEPS**	**Avaliação Clínica**	**Tipo de exercício**	**Tempo**	**Intensidade**	**Dose**
**0**	KILIP IV/III<br>OU TIMI 0-1	Sedestação passiva no leito	2 horas	---	---
**1**	Ausência de sinais e sintomas de isquemia cardíaca >8 horas (ECG/Marcadores isquêmicos/Dor precordial) ou descompensação cardíaca.	Aeróbio: Cicloergômetro de MMII no leito<br><br>Resistência Muscular de MMSS e MMII (Flexão e abdução de ombro, quadríceps em bloco e tríplice flexão)	3-5 min<br><br>1 x 10	Borg 9-10 (2 METs)	10MET-min
**2**	Ausência ou baixa dose de inotrópicos, vasodilatadores e anticoagulação endovenosa.	Aeróbio: Cicloergômetro de MMII na poltrona<br><br>Resistência Muscular de MMSS e MMII (Flexão e abdução de ombro, quadríceps em bloco e tríplice flexão)	6-10min<br><br>2 x 10	Borg 9-10 (2 METs)	20MET-min
**3**	Manutenção do quadro clínico e condições especificadas anteriormente.	Aeróbico: Cicloergômetro de MMII na poltrona + Deambulação (10 metros)<br>Resistência Muscular: Teste de sentar e levantar (30s)	9-15min	Borg 9-10 (2 METs)	30MET.min

**4**	Manutenção do quadro clínico e condições especificadas anteriormente.	Aeróbico: Cicloergômetro de MMII na poltrona + Deambulação (75-100metros)<br><br>Resistência Muscular: Exercício de sentar e levantar	9-15min<br><br>2 x 60% repetições do teste	Borg 9-10 (2 METs)	30MET.min

Legenda: A frequência dos STEPS é de 2 vezes ao dia seguindo a ordem dos STEPS. A progressão dos exercícios é feita com foco na varivél tempo de exercício e segue a ordem dos STEPS. O objetivo do protocolo é garantir um acumulado de **90 MET.min** até a alta da UTI.

Protocolo de RC precoce – ETAPA 2

STEPS	Avaliação Clínica	Tipo de exercício	Tempo	Intensidade	Dose
**5**	Manutenção do quadro clínico e condições especificadas anteriormente.	Aeróbico: Deambulação + Treino de escadas<br><br>Resistência Muscular: Exercício de sentar e levantar	9-15min<br><br>2 x 60% repetições do teste	Borg 11-12 (3 METs)	45MET.min
**6**	Manutenção do quadro clínico e condições especificadas anteriormente.	Aeróbico: Deambulação + Treino de escadas<br><br>Resistência Muscular: Exercício de sentar e levantar	9-15min<br><br>2 x 60% repetições do teste	Borg 11-12 (3 METs)	45MET.min
**7**	Manutenção do quadro clínico e condições especificadas anteriormente.	Aeróbico: Deambulação + Treino de escadas<br><br>Resistência Muscular:	12-20min<br><br>3 x 60% repetiçõ	Borg 11-12 (3 METs)	60MET.min

		Exercício de sentar e levantar	es do teste		
**8**	Manutenção do quadro clínico e condições especificadas anteriormente.	Aeróbico: Deambulação + Treino de escadas<br><br>Resistência Muscular: Exercício de sentar e levantar	12-20min<br><br>3 x 60% repetições do teste	Borg 11-12 (3 METs)	60MET.min

Legenda: A frequência dos STEPS é de 2 vezes ao dia seguindo a ordem dos STEPS. A progressão dos exercícios é feita com foco na varivél tempo e intensidade do exercício e segue a ordem dos STEPS. O objetivo do protocolo é garantir um acumulado de **210 MET.min** até a alta da enfermaria e **300 MET.min/sem** até a alta hospitalar.

APÊNDICE C – FICHA DE PRESCRIÇÃO E ACOMPANHAMENTO DA ETAPA 3 DA RC PRECOCE

Protocolo de RC precoce – ETAPA 3

**STEPS**	**Tipo de exercício**	**Frequência**	**Tempo**	**Intensidade**	**Dose**	**Diário semanal**
**1**	Aeróbico: Caminhada	5 x semana	20-25min	Borg 11-12 (3 METs)	300 MET.min-sem	F:___ T:________ BORG:____ Dose:______
**2**	Aeróbico: Caminhada	5 x semana	25-30min	Borg 11-12 (3 METs)	375 MET.min-sem	F:___ T:________ BORG:____ Dose:______
**3**	Aeróbico: Caminhada	5 x semana	30-35min	Borg 11-12 (3 METs)	450 MET.min-sem	F:___ T:________ BORG:____ Dose:_____
**4**	Aeróbico: Caminhada	5 x semana	35-40min	Borg 11-12 (3 METs)	525 MET.min-sem	F:___ T:________ BORG:____ Dose:_____

Legenda: Na etapa 3 os STEPS equivalem a semana de treinamento.

APÊNDICE D – TERMO DE CONSENTIMENTO LIVRE E ESCLARECIDO

TERMO DE CONSENTIMENTO LIVRE E ESCLARECIDO

O Sr. (a) está sendo convidado (a) como voluntário (a) a participar da pesquisa **"EFICÁCIA DA REABILITAÇÃO CARDÍACA PRECOCE APÓS INFARTO AGUDO DO MIOCÁRDIO: ENSAIO CLÍNICO RANDOMIZADO"**. Este estudo irá analisar os resultados da reabilitação cardíaca, utilizando-se da revisão do seu prontuário e de dois momentos de avaliações com exames físicos e de imagem. Esta pesquisa é importante para se entender o efeito da reabilitação cardíaca precoce sobre sua capacidade física e a função do seu coração de forma que os profissionais possam estabelecer formas mais eficazes de abordagem durante este processo.

Para este estudo adotaremos os seguintes procedimentos: Inicialmente serão analisados seus dados através de seu prontuário; Na primeira fase, o Sr.(a) participante será convidado a realizar um protocolo de reabilitação intra-hospitalar com sessões diárias de cerca 3 a 20 minutos e avaliação por meio de teste físico através do movimento de sentar e levantar de uma cadeira padronizada por 30 segundos e fará um exame de imagem do tipo ressonância magnética do coração, exame de imagem que dura cerca de 20 minutos, durante sua estadia no hospital. Na segunda etapa, após alta do hospital, você receberá um cartilha com orientações de exercícios para realizar em seu domicílio com sessões diárias de cerca de 20 a 40 minutos e após 30 dias do seu procedimento, você será novamente convidado a uma consulta ambulatorial no hospital para repetição do teste de sentar e levantar e realizar um teste na esteira ergométrica com duração média de 8 a 12 minutos e por fim realizará conforme marcação prévia um novo exame de imagem do seu coração através da ressonância cardíaca com a mesma duração do primeiro exame, cerca de 20 minutos.

Esta pesquisa poderá trazer benefícios a sua saúde física, emocional e capacidade de realizar suas atividades da vida diária através de uma prescrição individualizada dos exercícios físicos mais indicados e seguros a seremrealizados durante sua rotina, podendo inclusive diminuir o tempo da sua internação hospitalar e lhe dando mais segurança para sua alta hospitalar e qualidade de vida. Além disso, você receberá os laudos médicos a cerca do teste de esteira e ressonância magnética do seu coração, que poderá contribuir para um tratamento clínico mais eficaz junto ao seu cardiologista. Por fim, a sua participação nesta pesquisa ajudará no entendimento da melhor estratégia de reabilitação com exercícios após o infarto, facilitando o tratamento de diversos outros pacientes com este mesmo caso e favorecendo a publicação destes resultados para profissionais no mundo inteiro.

Rubrica do Participante/Responsável legal:	Rubrica do Pesquisador:

1/3

Av. Nilo Peçanha, 620, Petrópolis – Gerência de Ensino e Pesquisa - Prédio Administrativo - 3º andar
CEP 59.012-300 Natal/RN - Fone: (84) 3342-5027 - E-mail: gep_huol@outlook.com

Esta pesquisa pode apresentar como possíveis riscos: A perda ou divulgação de seus dados pessoais; Cansaço, mudança na pressão arterial, aumento da frequência de batimento do seu coração e sinais de tontura após o exercício ou teste físico. Os possíveis riscos serão evitados ao máximo, pois estaremos monitorando continuamente os seus sinais vitais e interromperemos o teste ou o treinamento para que não haja nenhum risco ao participante, além de fornecer suporte clínico nas unidades de internação do hospital. Além disso, os seus dados pessoais serão protegidos através de um bom manuseio dos dados com uso de computador exclusivo para a pesquisa e seguiremos as normas da Lei Geral de Proteção dos Dados (LGPD), lei nº 13.709.

Para participar deste estudo você não terá nenhum custo, nem receberá qualquer vantagem financeira. Você será esclarecido (a) sobre o estudo em qualquer aspecto que desejar e estará livre para participar ou recusar-se a participar, podendo deixar de participar ou retirar seu consentimento em qualquer fase ou momento da pesquisa, sem nenhum prejuízo para você. Em caso de algum problema que você possa ter relacionado com a pesquisa, ou mesmo alguma dúvida, você terá direito a assistência gratuita que será prestada pela pesquisadora M.a. Caroline Ferreira Schon, telefone (84) 99900-0359. Se você sofrer algum dano comprovadamente decorrente dessa pesquisa, você receberá a assistência necessária para seu reparo. Seus dados serão confidenciais e divulgados apenas em congressos ou publicações científicas, não havendo divulgação de nenhuma informação que possa lhe identificar. Esses dados serão guardados pelo pesquisador por um período de 5 anos.

Qualquer dúvida sobre a ética dessa pesquisa você deverá entrar em contato com o comitê de ética em pesquisa do Hospital Universitário Onofre Lopes, telefono: 3342-5003, endereço: Av. Nilo Peçanha, 620- Petrópolis-Espaço João Machado-1ºandar-Prédio Administrativo-CEP 59.012-300-Natal/RN, e-mail. Este documento foi impresso em duas vias. Uma ficará com você e a outra com o pesquisador responsável M.a. Caroline Ferreira Schon.

Consentimento Livre e Esclarecido

Após ter sido esclarecido sobre os objetivos, importância e o modo como os dados serão coletados nessa pesquisa, além de conhecer os riscos, desconfortos e benefícios que ela trará para mim e ter ficado ciente de todos os meus direitos, concordo em participar da pesquisa (**EFICÁCIA DA REABILITAÇÃO CARDÍACA PRECOCE APÓS INFARTO AGUDO DO MIOCÁRDIO: ENSAIO CLÍNICO RANDOMIZADO)** e autorizo a divulgação das informações por mim fornecidas em congressos e/ou publicações científicas desde que nenhum dado possa me identificar.

Rubrica do Participante/Responsável legal:	Rubrica do Pesquisador:

Av. Nilo Peçanha, 620, Petrópolis – Gerência de Ensino e Pesquisa - Prédio Administrativo - 3º andar
CEP 59.012-300 Natal/RN - Fone: (84) 3342-5027 - E-mail: gep_huol@outlook.com

Natal,________________de__________________de 2022.

____

Assinatura do participante da pesquisa

Impressão datiloscópica do participante

Como pesquisador responsável pelo estudo **(EFICÁCIA DA REABILITAÇÃO CARDÍACA PRECOCE APÓS INFARTO AGUDO DO MIOCÁRDIO: ENSAIO CLÍNICO RANDOMIZADO)**, declaro que assumo a inteira responsabilidade de cumprir fielmente os procedimentos metodologicamente e direitos que foram esclarecidos e assegurados ao participante desse estudo, assim como manter sigilo e confidencialidade sobre a identidade do mesmo.

Declaro ainda estar ciente que na inobservância do compromisso ora assumido estarei infringindo as normas e diretrizes propostas pela Resolução 466/12 do Conselho Nacional de Saúde – CNS, que regulamenta as pesquisas envolvendo o ser humano.

Natal,__________de__________________de 2022.

____

M.a. Caroline Ferreira Schon

Pesquisadora Responsável

Rubrica do Participante/Responsável legal:	Rubrica do Pesquisador:

Av. Nilo Peçanha, 620, Petrópolis – Gerência de Ensino e Pesquisa - Prédio Administrativo - 3º andar
CEP 59.012-300 Natal/RN - Fone: (84) 3342-5027 - E-mail: gep_huol@outlook.com

APÊNDICE E – TERMO DE AUTORIZAÇÃO INSTITUCIONAL PARA USO DE DOCUMENTOS DOS PACIENTES

TERMO DE AUTORIZAÇÃO INSTITUCIONAL PARA USO DE DOCUMENTOS DOS PACIENTES

Ilmo. Prof. Dr. Carlos Alberto Almeida de Araújo
Gerente de Ensino e Pesquisa do HUOL/EBSERH.

O Departamento de Fisioterapia da Universidade Federal do Rio Grande do Norte realizará uma pesquisa sob a orientação do Prof(a). Dr(a). Selma Sousa Bruno intitulada "**EFICÁCIA DA REABILITAÇÃO CARDÍACA PRECOCE APÓS INFARTO AGUDO DO MIOCÁRDIO: ENSAIO CLÍNICO RANDOMIZADO**". Este estudo aborda uma análise dos resultados da reabilitação cardíaca (RC) precoce, utilizando-se da revisão dos prontuários e avaliações durante o início da RC e em até 90 dias após a alta hospitalar do paciente. Esta pesquisa é importante para se entender o efeito da RC precoce de forma que os profissionais possam estabelecer formas mais eficazes de abordagem durante a RC.

Assim sendo, solicitamos de V.Sª. a valiosa colaboração, no sentido de autorizar o acesso e utilização dos prontuários e resultados de exames pela pesquisadora responsável M.a. Caroline Ferreira Schon e pela orientadora Dra. Selma Sousa Bruno. Salientamos que os dados coletados serão mantidos em sigilo e utilizados tão somente para realização deste estudo, minimizando o risco de exposição dos pacientes. Serão tomadas as devidas precauções para que não haja danos aos documentos; ou seja, os pesquisadores se comprometem a manusear os documentos em ambiente reservado e destinado para isso e não retirá-los do local de origem, não fotografar ou fazer cópia de qualquer natureza. Mesmo com os cuidados tomados pelos pesquisadores com os pacientes, donos dos documentos cedidos, caso ocorra danos proveniente da pesquisa, os mesmos serão devidamente indenizados pelos pesquisadores. Esta pesquisa apresenta como benefícios: o fornecimento do laudo médico a cerca do teste ergométrico que poderá auxiliar no melhor controle clínico com seu cardiologista e receberá uma cartilha personalizada com orientações a cerca dos exercícios melhor indicado e seguro a ser realizado durante sua rotina conforme o resultado do teste.

Os dados serão guardados em local seguro na UFRN sob a responsabilidade da Coordenadora da pesquisa Dra. Selma Sousa Bruno e a divulgação dos resultados será feita de forma a não identificar os voluntários. A pesquisa não acarretará despesas nem para esta Instituição – Hospital Universitário Onofre Lopes/UFRN, nem para os sujeitos de pesquisa.

Av. Nilo Peçanha, 620, Petrópolis – Gerência de Ensino e Pesquisa - Prédio Administrativo - 3º andar
CEP 59.012-300 Natal/RN - Fone: (84) 3342-5027 - E-mail: gep_huol@outlook.com

No entanto, se surgir alguma despesa não prevista, referente à participação do HUOL ou dos sujeitos, nos comprometemos em ressarcir tais despesas, mediante solicitação. A participação do Centro é voluntária, o que significa que V.Sª. poderá desistir a qualquer momento, retirando seu consentimento, sem que isso traga nenhum prejuízo ou penalidade para a Instituição/HUOL/UFRN ou pacientes donos dos documentos. V.Sa. ficará com uma via deste Termo, elaborado em duas laudas, e toda dúvida que tiveres a respeito desta pesquisa, poderá perguntar diretamente a M.a. Caroline Ferreira Schon, pesquisadora responsável desta pesquisa, telefone: (84) 999000359, e-mail: caroline.schon@ebserh.gov.br ou a Prof. Dra. Selma Sousa Bruno, orientadora deste projeto no Departamento de Fisioterapia; telefone: (84) (999817854), ou e –mail (sbruno@ufrnet.br).

Dúvidas a respeito da ética dessa pesquisa poderão ser questionadas ao Comitê de Ética em Pesquisa do HUOL, localizado na Av. Nilo Peçanha, 620, Petrópolis - Prédio Administrativo - 1º andar – Espaço João Machado, Telefone (84) 3342-5003.

Declaro ainda estar ciente que na inobservância do compromisso ora assumido estarei infringindo as normas e diretrizes propostas pela Resolução 466/12 do Conselho Nacional de Saúde – CNS, que regulamenta as pesquisas envolvendo o ser humano.

Contando com a compreensão desta Direção, agradecemos antecipadamente.

Natal,_____de___________2022.

M.a. Caroline Ferreira Schon

Pesquisadora Responsável

( ) Concordamos com a solicitação ( ) Não concordamos com a solicitação

Prof. Dr. Carlos Alberto Almeida de Araújo

Gerente de Ensino e Pesquisa - HUOL/EBSERH

APÊNDICE F – CARTILHA DE ORIENTAÇÕES DE EXERCÍCIOS FÍSICOS

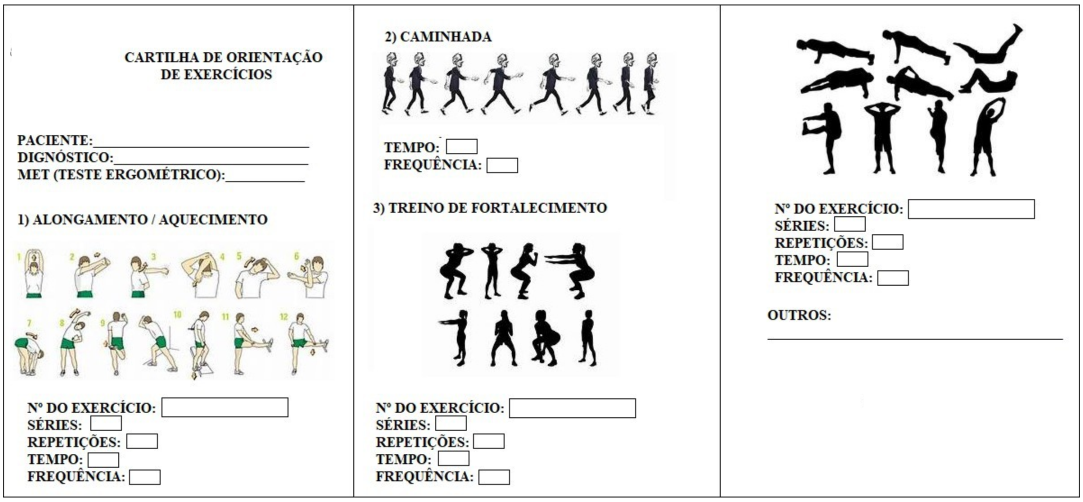

CARTILHA DE ORIENTAÇÃO
DE EXERCÍCIOS

PACIENTE:____________________________
DIGNÓSTICO:__________________________
MET (TESTE ERGOMÉTRICO):___________

1) ALONGAMENTO / AQUECIMENTO

Nº DO EXERCÍCIO:
SÉRIES:
REPETIÇÕES:
TEMPO:
FREQUÊNCIA:

2) CAMINHADA

TEMPO:
FREQUÊNCIA:

3) TREINO DE FORTALECIMENTO

Nº DO EXERCÍCIO:
SÉRIES:
REPETIÇÕES:
TEMPO:
FREQUÊNCIA:

Nº DO EXERCÍCIO:
SÉRIES:
REPETIÇÕES:
TEMPO:
FREQUÊNCIA:

OUTROS:
____

ANEXOS

ANEXO A – ESCALA DE PERCEPÇÃO DE ESFORÇO (BORG)

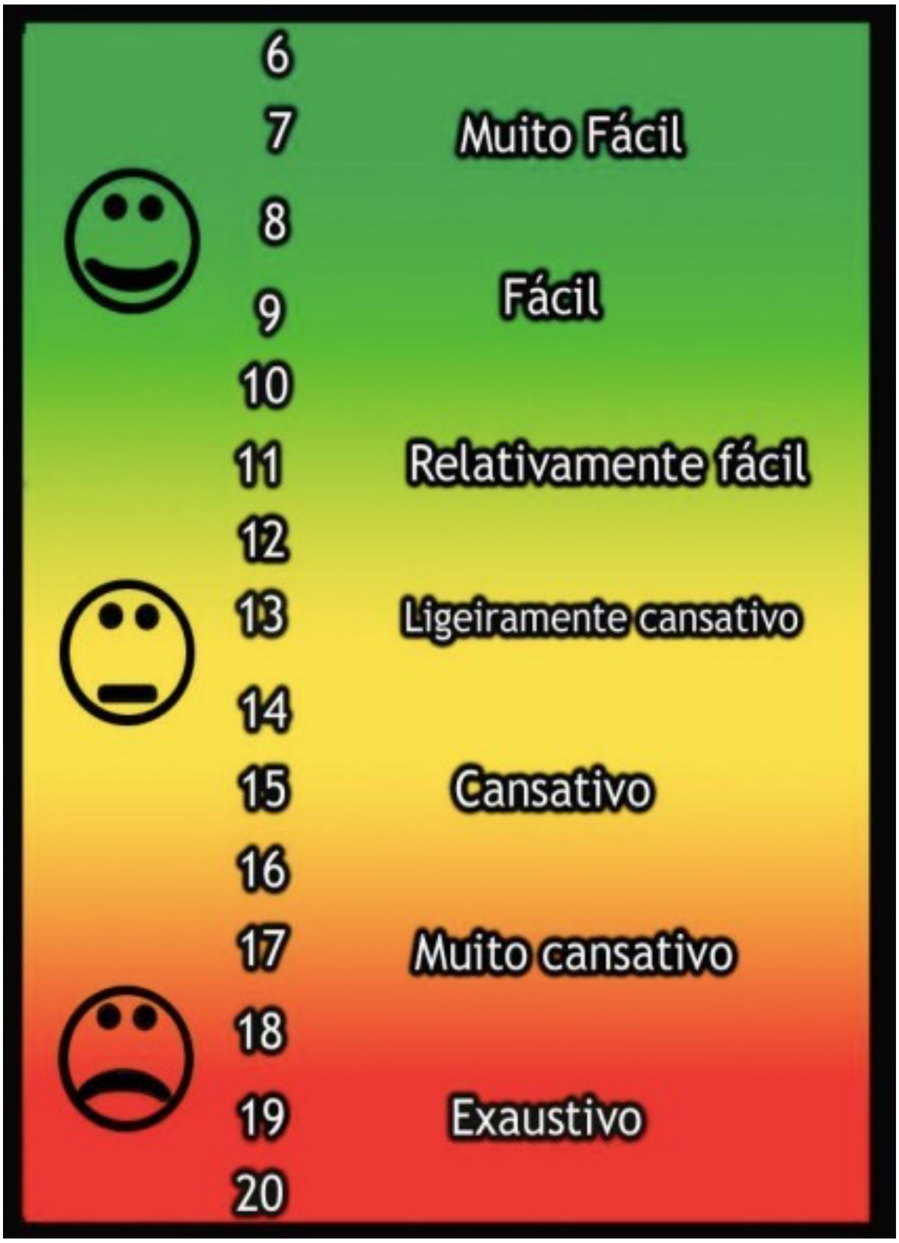

Supplement: S2 Protocol — (PDF) [file pone.0296345.s004.pdf]
