# Supplementary material for: Efficacy of early cardiac rehabilitation after acute myocardial infarction: Randomized clinical trial protocol
Source: PLoS One. 2024 Jan 10;19(1):e0296345. doi: 10.1371/journal.pone.0296345 (PMC10781044; doi:10.1371/journal.pone.0296345)
Supplement: Attachment — Submitted filename: Response to Reviewers.pdf [file pone.0296345.s005.pdf]

PONE-D-23-26645
Efficacy of Early Cardiac Rehabilitation after Acute Myocardial Infarction: Randomized Clinical Trial Protocol

First, we would like to thank you for each suggestion, doubt, and comment. Your contributions give us the chance to improve the possibility of this manuscript being more readable to PLOS One readers.

Our response will be placed below each concern.

Reviewer #1: I have reviewed the protocol paper entitled "Efficacy of Early Cardiac Rehabilitation after Acute Myocardial Infarction: Randomized Clinical Trial Protocol".

In this protocol, three co-primary outcomes will be assessed.
But the sample size is relatively small, which was calculated using the mean difference of maximum oxygen consumption.
Does this have enough power to prove differences of the other co-primary outcomes?

We revised carefully the HRV (heart rate variability) and RNM (Remodeling based on Magnetic Resonance) of our co-primary outcomes, including adding a new sample size calculated for these 2 variables. We performed 3 sample size calculations to the VO_2max, HRV, and remodeling with β=20 and α=0.05. We basedour thinking on *Izeli N. L, et al. Aerobic Training after Myocardial Infarction: Remodeling Evaluated by Cardiac Magnetic Resonance. 2016, and Hiss M.D.B, et al. Effects of progressive exercise during phase I cardiac rehabilitation on the heart rate variability of patients with acute myocardial infarction. 2011.* Our data were similar to those in terms of procedure, clinical assessment, and clinical state of disease. Thefirst study we reached a sample size of 10 participants/group considering remodeling (left ventricle mass as variable chosen) as a primary outcome. In the second study, it was reached 14 patients/group (time domain variables) and less than 10 participants per group (frequency domain variables). Our sample size was based on VO_2 max, due to this variable being more representative of functional capacity. As the sample size calculated for these 3 variables is similar, and considering all the issues together, we believe we can keep the VO_2max as suitable to reach our sample size.

Reviewer #2: This is an interesting study protocol about early rehabilitation in patients with AMI. I have a few comments to the authors.

1. If I understand correctly, there are many studies about early rehabilitation in patients with AMI: See Cochrane Database Syst Rev (1) (2016), Article CD001800. A meta-analysis of the effects of exercise training on left ventricular remodeling following myocardial infarction: start early and go longer for greatest exercise benefits on remodeling Trials, 12 (2011), p. 92. What is the difference of this trial from previous studies?

Thank you for the raised issues. In fact, we revised all these papers, and we have considered the evidence already documented for ventricular remodeling however we pointed out 3 main differences:

1. **Time of onset of cardiac rehabilitation. 8 hours after clinical stabilization. It will never after 3 days due to residual pain be an exclusion criterion.**

In both systematic review (Anderson et al., 2016) review and meta-analysis (Mark Haykowsky et al. 2011) had a high heterogeneity in terms of the time of initiation of cardiac rehabilitation. Regarding ventricular remodeling, the data meta analyzed were from 1 week after myocardial infarction.

2. **The main scientific interest in the very early initiation of exercise is focused on hemodynamic safety versus dose of exercise. That is a primary aim. We have no pieces of evidence until now from isolated studies or systematic reviews.**
3. **A very well-documented exercise prescription (to this clinical condition x time prescription) in terms of dose (intensity of FITT will be modified day by day considering increasing of METs). Also, we are still without a body of evidence from isolated studies or systematic reviews.**

2. Why the authors set the inclusion criteria: age 20 to 70 years? Rehabilitation would be more beneficial in elder patients (>70 years).

Yes, we completely agree with this evidence. However, we also were worried about the combined effects of aging among older patients, especially the possibility of the association with cardiovascular dysfunction, and skeletal-muscle dysfunction could have a combined effect on physical function and also delay the in-patient period.

Dalane, et al. Physical Rehabilitation for Older Patients Hospitalized for Heart Failure. N Engl J Med 2021;385:203-16. DOI: 10.1056/NEJMoa2026141
