# Supplementary material for: Efficacy of early cardiac rehabilitation after acute myocardial infarction: Randomized clinical trial protocol
Source: PLoS One. 2024 Jan 10;19(1):e0296345. doi: 10.1371/journal.pone.0296345 (PMC10781044; doi:10.1371/journal.pone.0296345)
Supplement: Peer review history

# Peer review: Decision Letter 0

**DOI:** 10.1371/journal.pone.0296345.r001

**Authors:** Yoshihiro Fukumoto

31 Oct 2023

PONE-D-23-26645Efficacy of Early Cardiac Rehabilitation after Acute Myocardial Infarction: Randomized Clinical Trial ProtocolPLOS ONE

Dear Dr. Bruno,

Thank you for submitting your manuscript to PLOS ONE. After careful consideration, we feel that it has merit but does not fully meet PLOS ONE’s publication criteria as it currently stands. Therefore, we invite you to submit a revised version of the manuscript that addresses the points raised during the review process.

Please submit your revised manuscript by Dec 15 2023 11:59PM. If you will need more time than this to complete your revisions, please reply to this message or contact the journal office at plosone@plos.org. When you're ready to submit your revision, log on to https://www.editorialmanager.com/pone/ and select the 'Submissions Needing Revision' folder to locate your manuscript file.

Please include the following items when submitting your revised manuscript:

- A rebuttal letter that responds to each point raised by the academic editor and reviewer(s). You should upload this letter as a separate file labeled 'Response to Reviewers'.
- A marked-up copy of your manuscript that highlights changes made to the original version. You should upload this as a separate file labeled 'Revised Manuscript with Track Changes'.
- An unmarked version of your revised paper without tracked changes. You should upload this as a separate file labeled 'Manuscript'.

If you would like to make changes to your financial disclosure, please include your updated statement in your cover letter. Guidelines for resubmitting your figure files are available below the reviewer comments at the end of this letter.

If applicable, we recommend that you deposit your laboratory protocols in protocols.io to enhance the reproducibility of your results. Protocols.io assigns your protocol its own identifier (DOI) so that it can be cited independently in the future. For instructions see: https://journals.plos.org/plosone/s/submission-guidelines#loc-laboratory-protocols. Additionally, PLOS ONE offers an option for publishing peer-reviewed Lab Protocol articles, which describe protocols hosted on protocols.io. Read more information on sharing protocols at https://plos.org/protocols?utm_medium=editorial-email&utm_source=authorletters&utm_campaign=protocols.

We look forward to receiving your revised manuscript.

Kind regards,

Yoshihiro Fukumoto

Academic Editor

PLOS ONE

Journal Requirements:

When submitting your revision, we need you to address these additional requirements.

1. Please ensure that your manuscript meets PLOS ONE's style requirements, including those for file naming. The PLOS ONE style templates can be found at

https://journals.plos.org/plosone/s/file?id=wjVg/PLOSOne_formatting_sample_main_body.pdf and

https://journals.plos.org/plosone/s/file?id=ba62/PLOSOne_formatting_sample_title_authors_affiliations.pdf

2. We note that the grant information you provided in the ‘Funding Information’ and ‘Financial Disclosure’ sections do not match. 

When you resubmit, please ensure that you provide the correct grant numbers for the awards you received for your study in the ‘Funding Information’ section.

[Note: HTML markup is below. Please do not edit.]

Reviewers' comments:

Reviewer's Responses to Questions

**Comments to the Author**

1. Does the manuscript provide a valid rationale for the proposed study, with clearly identified and justified research questions?

The research question outlined is expected to address a valid academic problem or topic and contribute to the base of knowledge in the field.

Reviewer #1: Yes

Reviewer #2: Yes

**********

2. Is the protocol technically sound and planned in a manner that will lead to a meaningful outcome and allow testing the stated hypotheses?

The manuscript should describe the methods in sufficient detail to prevent undisclosed flexibility in the experimental procedure or analysis pipeline, including sufficient outcome-neutral conditions (e.g. necessary controls, absence of floor or ceiling effects) to test the proposed hypotheses and a statistical power analysis where applicable. As there may be aspects of the methodology and analysis which can only be refined once the work is undertaken, authors should outline potential assumptions and explicitly describe what aspects of the proposed analyses, if any, are exploratory.

Reviewer #1: Yes

Reviewer #2: Yes

**********

3. Is the methodology feasible and described in sufficient detail to allow the work to be replicable?

Descriptions of methods and materials in the protocol should be reported in sufficient detail for another researcher to reproduce all experiments and analyses. The protocol should describe the appropriate controls, sample size calculations, and replication needed to ensure that the data are robust and reproducible.

Reviewer #1: No

Reviewer #2: Yes

**********

4. Have the authors described where all data underlying the findings will be made available when the study is complete?

The PLOS Data policy requires authors to make all data underlying the findings described in their manuscript fully available without restriction, with rare exception, at the time of publication. The data should be provided as part of the manuscript or its supporting information, or deposited to a public repository. For example, in addition to summary statistics, the data points behind means, medians and variance measures should be available. If there are restrictions on publicly sharing data—e.g. participant privacy or use of data from a third party—those must be specified.

Reviewer #1: No

Reviewer #2: No

**********

5. Is the manuscript presented in an intelligible fashion and written in standard English?

PLOS ONE does not copyedit accepted manuscripts, so the language in submitted articles must be clear, correct, and unambiguous. Any typographical or grammatical errors should be corrected at revision, so please note any specific errors here.

Reviewer #1: Yes

Reviewer #2: Yes

**********

6. Review Comments to the Author

Please use the space provided to explain your answers to the questions above and, if applicable, provide comments about issues authors must address before this protocol can be accepted for publication. You may also include additional comments for the author, including concerns about research or publication ethics.

You may also provide optional suggestions and comments to authors that they might find helpful in planning their study.

(Please upload your review as an attachment if it exceeds 20,000 characters)

Reviewer #1: I have reviewed the protocol paper entitled “Efficacy of Early Cardiac Rehabilitation after Acute Myocardial Infarction: Randomized Clinical Trial Protocol”.

I feel that this report is well-summarized and well-written.

I have one comment.

In this protocol, three co-primary outcomes will be assessed.

But the sample size is relatively small, which was calculated using the mean difference of maximum oxygen consumption.

Does this have enough power to prove differences of the other co-primary outcomes?

Reviewer #2: This is an interesting study protocol about early rehabilitation in patients with AMI. I have a few comments to the authors.

1. If I understand correctly, there are many studies about early rehabilitation in patients with AMI: See Cochrane Database Syst Rev (1) (2016), Article CD001800. A meta-analysis of the effects of exercise training on left ventricular remodeling following myocardial infarction: start early and go longer for greatest exercise benefits on remodeling Trials, 12 (2011), p. 92. What is the difference of this trial from previous studies?

2. Why the authors set the inclusion criteria: age 20 to 70 years? Rehabilitation would be more beneficial in elder patients (>70 years).

**********

7. PLOS authors have the option to publish the peer review history of their article (what does this mean?). If published, this will include your full peer review and any attached files.

If you choose “no”, your identity will remain anonymous but your review may still be made public.

**Do you want your identity to be public for this peer review?** For information about this choice, including consent withdrawal, please see our Privacy Policy.

Reviewer #1: No

Reviewer #2: No

**********

[NOTE: If reviewer comments were submitted as an attachment file, they will be attached to this email and accessible via the submission site. Please log into your account, locate the manuscript record, and check for the action link "View Attachments". If this link does not appear, there are no attachment files.]

While revising your submission, please upload your figure files to the Preflight Analysis and Conversion Engine (PACE) digital diagnostic tool, https://pacev2.apexcovantage.com/. PACE helps ensure that figures meet PLOS requirements. To use PACE, you must first register as a user. Registration is free. Then, login and navigate to the UPLOAD tab, where you will find detailed instructions on how to use the tool. If you encounter any issues or have any questions when using PACE, please email PLOS at figures@plos.org. Please note that Supporting Information files do not need this step.

# Author comment: Author response to Decision Letter 0

**DOI:** 10.1371/journal.pone.0296345.r002

30 Nov 2023

First, we would like to thank you for each suggestion, doubt, and comment. Your contributions give us the chance to improve the possibility of this manuscript being more readable to PLOS One readers.

Our response will be placed below each concern.

Reviewer #1: I have reviewed the protocol paper entitled “Efficacy of Early Cardiac Rehabilitation after Acute Myocardial Infarction: Randomized Clinical Trial Protocol”.

In this protocol, three co-primary outcomes will be assessed.

But the sample size is relatively small, which was calculated using the mean difference of maximum oxygen consumption.

Does this have enough power to prove differences of the other co-primary outcomes?

We revised carefully the HRV (heart rate variability) and RNM (Remodeling based on Magnetic Resonance) of our co-primary outcomes, including adding a new sample size calculated for these 2 variables. We performed 3 sample size calculations to the VO2max, HRV, and remodeling with b=20 and a=0.05. We basedour thinking on Izeli N. L, et al. Aerobic Training after Myocardial Infarction: Remodeling Evaluated by Cardiac Magnetic Resonance. 2016, and Hiss M.D.B, et al. Effects of progressive exercise during phase I cardiac rehabilitation on the heart rate variability of patients with acute myocardial infarction. 2011. Our data were similar to those in terms of procedure, clinical assessment, and clinical state of disease. Thefirst study we reached a sample size of 10 participants/group considering remodeling (left ventricle mass as variable chosen) as a primary outcome. In the second study, it was reached 14 patients/group (time domain variables) and less than 10 participants per group (frequency domain variables). Our sample size was based on VO2 max, due to this variable being more representative of functional capacity. As the sample size calculated for these 3 variables is similar, and considering all the issues together, we believe we can keep the VO2max as suitable to reach our sample size.

Reviewer #2: This is an interesting study protocol about early rehabilitation in patients with AMI. I have a few comments to the authors.

1. If I understand correctly, there are many studies about early rehabilitation in patients with AMI: See Cochrane Database Syst Rev (1) (2016), Article CD001800. A meta- analysis of the effects of exercise training on left ventricular remodeling following myocardial infarction: start early and go longer for greatest exercise benefits on remodeling Trials, 12 (2011), p. 92. What is the difference of this trial from previous studies?

Thank you for the raised issues. In fact, we revised all these papers, and we have considered the evidence already documented for ventricular remodeling however we pointed out 3 main differences:

1. Time of onset of cardiac rehabilitation. 8 hours after clinical stabilization. It will never after 3 days due to residual pain be an exclusion criterion.

In both systematic review (Anderson et al., 2016) review and meta-analysis (Mark Haykowsky et al. 2011) had a high heterogeneity in terms of the time of initiation of cardiac rehabilitation. Regarding ventricular remodeling, the data metaanalyzed were from 1 week after myocardial infarction.

2. The main scientific interest in the very early initiation of exercise is focused on hemodynamic safety versus dose of exercise. That is a primary aim. We have no pieces of evidence until now from isolated studies or systematic reviews.

3. A very well-documented exercise prescription (to this clinical condition x time prescription) in terms of dose (intensity of FITT will be modified day by day considering increasing of METs). Also, we are still without a body of evidence from isolated studies or systematic reviews.

2. Why the authors set the inclusion criteria: age 20 to 70 years? Rehabilitation would be more beneficial in elder patients (>70 years).

Yes, we completely agree with this evidence. However, we also were worried about the combined effects of aging among older patients, especially the possibility of the association with cardiovascular dysfunction, and skeletal-muscle dysfunction could have a combined effect on physical function and also delay the in-patient period.

Dalane, et al. Physical Rehabilitation for Older Patients Hospitalized

for Heart Failure. N Engl J Med 2021;385:203-16. DOI: 10.1056/NEJMoa2026141

Attachment Submitted filename: Response to Reviewers.pdf Click here for additional data file.

# Peer review: Decision Letter 1

**DOI:** 10.1371/journal.pone.0296345.r003

**Authors:** Yoshihiro Fukumoto

11 Dec 2023

Efficacy of Early Cardiac Rehabilitation after Acute Myocardial Infarction: Randomized Clinical Trial Protocol

PONE-D-23-26645R1

Dear Dr. Bruno,

We’re pleased to inform you that your manuscript has been judged scientifically suitable for publication and will be formally accepted for publication once it meets all outstanding technical requirements.

Within one week, you’ll receive an e-mail detailing the required amendments. When these have been addressed, you’ll receive a formal acceptance letter and your manuscript will be scheduled for publication.

An invoice for payment will follow shortly after the formal acceptance. To ensure an efficient process, please log into Editorial Manager at http://www.editorialmanager.com/pone/, click the 'Update My Information' link at the top of the page, and double check that your user information is up-to-date. If you have any billing related questions, please contact our Author Billing department directly at authorbilling@plos.org.

If your institution or institutions have a press office, please notify them about your upcoming paper to help maximize its impact. If they’ll be preparing press materials, please inform our press team as soon as possible -- no later than 48 hours after receiving the formal acceptance. Your manuscript will remain under strict press embargo until 2 pm Eastern Time on the date of publication. For more information, please contact onepress@plos.org.

Kind regards,

Yoshihiro Fukumoto

Academic Editor

PLOS ONE

Additional Editor Comments (optional):

Reviewers' comments:

Reviewer's Responses to Questions

**Comments to the Author**

1. Does the manuscript provide a valid rationale for the proposed study, with clearly identified and justified research questions?

The research question outlined is expected to address a valid academic problem or topic and contribute to the base of knowledge in the field.

Reviewer #1: Yes

Reviewer #2: Yes

**********

2. Is the protocol technically sound and planned in a manner that will lead to a meaningful outcome and allow testing the stated hypotheses?

The manuscript should describe the methods in sufficient detail to prevent undisclosed flexibility in the experimental procedure or analysis pipeline, including sufficient outcome-neutral conditions (e.g. necessary controls, absence of floor or ceiling effects) to test the proposed hypotheses and a statistical power analysis where applicable. As there may be aspects of the methodology and analysis which can only be refined once the work is undertaken, authors should outline potential assumptions and explicitly describe what aspects of the proposed analyses, if any, are exploratory.

Reviewer #1: Yes

Reviewer #2: Yes

**********

3. Is the methodology feasible and described in sufficient detail to allow the work to be replicable?

Descriptions of methods and materials in the protocol should be reported in sufficient detail for another researcher to reproduce all experiments and analyses. The protocol should describe the appropriate controls, sample size calculations, and replication needed to ensure that the data are robust and reproducible.

Reviewer #1: Yes

Reviewer #2: Yes

**********

4. Have the authors described where all data underlying the findings will be made available when the study is complete?

The PLOS Data policy requires authors to make all data underlying the findings described in their manuscript fully available without restriction, with rare exception, at the time of publication. The data should be provided as part of the manuscript or its supporting information, or deposited to a public repository. For example, in addition to summary statistics, the data points behind means, medians and variance measures should be available. If there are restrictions on publicly sharing data—e.g. participant privacy or use of data from a third party—those must be specified.

Reviewer #1: No

Reviewer #2: Yes

**********

5. Is the manuscript presented in an intelligible fashion and written in standard English?

PLOS ONE does not copyedit accepted manuscripts, so the language in submitted articles must be clear, correct, and unambiguous. Any typographical or grammatical errors should be corrected at revision, so please note any specific errors here.

Reviewer #1: Yes

Reviewer #2: Yes

**********

6. Review Comments to the Author

Please use the space provided to explain your answers to the questions above and, if applicable, provide comments about issues authors must address before this protocol can be accepted for publication. You may also include additional comments for the author, including concerns about research or publication ethics.

You may also provide optional suggestions and comments to authors that they might find helpful in planning their study.

(Please upload your review as an attachment if it exceeds 20,000 characters)

Reviewer #1: I have reviewed the author's response to my comment.

The author has responded to my question nicely.　　

Now I have no further comments.

Reviewer #2: The authors addressed my comments adequately. Now I understand the difference of this trial from previous studies. They also provide the reason for the inclusion criteria about not including the elderly. I do not have further comments.

**********

7. PLOS authors have the option to publish the peer review history of their article (what does this mean?). If published, this will include your full peer review and any attached files.

If you choose “no”, your identity will remain anonymous but your review may still be made public.

**Do you want your identity to be public for this peer review?** For information about this choice, including consent withdrawal, please see our Privacy Policy.

Reviewer #1: No

Reviewer #2: No

**********

# Editor report: Acceptance letter

**DOI:** 10.1371/journal.pone.0296345.r004

**Authors:** Yoshihiro Fukumoto

2 Jan 2024

PONE-D-23-26645R1 

PLOS ONE

Dear Dr. Bruno, 

I'm pleased to inform you that your manuscript has been deemed suitable for publication in PLOS ONE. Congratulations! Your manuscript is now being handed over to our production team.

At this stage, our production department will prepare your paper for publication. This includes ensuring the following:

* All references, tables, and figures are properly cited

* All relevant supporting information is included in the manuscript submission,

* There are no issues that prevent the paper from being properly typeset

If revisions are needed, the production department will contact you directly to resolve them. If no revisions are needed, you will receive an email when the publication date has been set. At this time, we do not offer pre-publication proofs to authors during production of the accepted work. Please keep in mind that we are working through a large volume of accepted articles, so please give us a few weeks to review your paper and let you know the next and final steps. 

Lastly, if your institution or institutions have a press office, please let them know about your upcoming paper now to help maximize its impact. If they'll be preparing press materials, please inform our press team within the next 48 hours. Your manuscript will remain under strict press embargo until 2 pm Eastern Time on the date of publication. For more information, please contact onepress@plos.org.

If we can help with anything else, please email us at customercare@plos.org.

Thank you for submitting your work to PLOS ONE and supporting open access. 

Kind regards, 

PLOS ONE Editorial Office Staff

on behalf of

Dr. Yoshihiro Fukumoto 

Academic Editor

PLOS ONE
